# Effects of dazomet combined with *Rhodopsesudomonas palustris* PSB-06 on root-knot nematode, *Meloidogyne incognita* infecting ginger and soil microorganisms diversity

**DOI:** 10.3389/fmicb.2022.1021445

**Published:** 2022-09-29

**Authors:** Dongwei Wang, Jian Wang, Pin Su, Jianping Dai, Xinqiu Tan, Deyong Zhang, Yong Liu, Feixue Cheng

**Affiliations:** ^1^Key Laboratory of Integrated Management of the Pests and Diseases on Horticultural Crops in Hunan Province, Institute of Plant Protection, Hunan Academy of Agriculture Sciences, Changsha, China; ^2^Agricultural Economy and Regional Planning Research Institute, Hunan Academy of Agricultural Sciences, Changsha, China

**Keywords:** *Rhodopseudomonas palustris* PSB-06, ginger, root-knot nematode, fumigant-dazomet, rhizosphere microorganisms

## Abstract

Root-knot nematode, *Meloidogyne incognita* is one of the most important nematodes affecting ginger crop. *Rhodopseudomonas palustris* PSB-06, as effective microbial fertilizer in increasing plant growth and suppressing soil-borne disease of many crops has been reported. The combination of *R. palustris* PSB-06 and dazomet treatments had been proved to inhibit root-knot nematode on ginger and increase ginger yield in our preliminary study. The field experiments were conducted to elucidate the reasons behind this finding, and followed by next-generation sequencing to determine the microbial population structures in ginger root rhizosphere. The results showed that combination of *R. palustris* PSB-06 and dazomet treatment had a synergetic effect by achieving of 80.00% reduction in root-knot nematode numbers less than soil without treatment, and also could increase 37.37% of ginger yield through increasing the contents of chlorophyll and total protein in ginger leaves. Microbiota composition and alpha diversity varied with treatments and growth stages, soil bacterial diversity rapidly increased after planting ginger. In addition, the combined treatment could increase diversity and community composition of probiotic bacteria, and decrease those of soil-borne pathogenic fungi comparing to the soil treated with dazomet alone. Meanwhile, it could also effectively increase soil organic matter, available phosphorus and available potassium. Analysis of correlation between soil microorganisms and physicochemical properties indicated that the soil pH value and available phosphorus content were important factors that could affect soil microorganisms structure at the harvest stage. The bacterial family was more closely correlated with the soil physicochemical properties than the fungal family. Therefore, the combination of *R. palustris* PSB-06 and dazomet was considered as an effective method to control root-knot nematode disease and improve ginger soil conditions.

## Introduction

Ginger (*Zingiber officinale* Roscoe) is a spiced vegetable and a dietary supplement traded worldwide (An et al., 2016), and could also be used as an herbal medicine to improve human health, including alleviation of nausea, appetite loss, motion sickness, and pain (An et al., 2016; Jaiswal et al., 2016; Zhao et al., 2020b). Root-knot nematodes (*Meloidogyne* spp.) are one of the most economically important plant-parasitic nematodes genera infecting more than 3,000 plant species, especially vegetables (Blok et al., 2008; Jones et al., 2013; Shahid et al., 2022). Root-knot nematode, *Meloidogyne incognita* is one of the most important nematodes affecting ginger crop in the Laiwu district in the Shandong Province, which is known to have over 2000 years ginger cultivation history (Wang et al., 2020), it could cause ginger bark crack, plant stunting, and reduction of main stem perimeter and tiller number, leading to 20–50% yield losses (Guo et al., 2005; Zhao et al., 2020a). Root-knot nematodes were extremely difficult to be managed because of having very wide host range and high rate of multiplication. The commonly control methods are using chemical nematicides and crop varieties resistant to nematodes (Zhao et al., 2020a; Ahmed et al., 2021; Azeem et al., 2021; Haq et al., 2022). However, the large scale applications of synthetic nematicides are associated with health and environmental concerns, many effective nematicides had been restricted from the market in recent years.

Dazomet (3,5-Dimethyl-1,3,5-thiadiazinane-2-thione) is a soil fumigant for many soil-borne pathogens and nematodes, it had been widely used to control soil-borne diseases in China, which had not only excellent control effect on neither nor soil-borne bacterial and fungal pathogens, but also plant parasitic nematodes (Jeffries et al., 2017; Nicola et al., 2017; Zhang et al., 2019b; Fang et al., 2021). The dazomet fumigation could rapidly decease plant parasitic nematodes in soil and maintain this control effect till 12 weeks (Eo and Park, 2014). However, because of their high rates of multiplication, they would be recovered to harm the ginger later. Furthermore, the effects on soil-borne beneficial bacteria, fungi, and non-plant-infecting nematodes are detrimental (Nicola et al., 2017; Hu et al., 2020), the population of soil fungi and bacteria were significantly decreased after the application of dazomet (Gao et al., 2018; Li et al., 2019; Ni et al., 2021). The microbial community of rhizosphere had specific plant habitat and was closely related to plant growth. Several reports had indicated that certain rhizosphere microorganisms could have beneficial, neutral or harmful effects on plant growth through interfering with root surface defense responses (Mendes et al., 2013; Shi et al., 2022). Therefore, soil dazomet fumigation could not be used alone in field to maintain sustainable development of soil microorganisms. To maintain soil microbial diversity, soil fumigation together with applications of biological control agents (BCAs) or plant growth promoting rhizobacteria (PGPRs) was considered as an effective control method during continuous crop cultivation. PGPRs or BCAs could promote plant growth and increase crop yields (Majeed et al., 2015; Etesami and Maheshwari, 2018; Koua et al., 2020; Valente et al., 2020) through increasing nutrient uptake, induction of plant growth regulators production, activation of plant defense responses, and production of antibiotics (e.g., fengycin, iturin, surfactin). It also could protect plants from soil-borne pathogens infections by changing soil physicochemical properties, and soil microbial structures and compositions (Lugtenberg and Kamilova, 2009; Lupwayi et al., 2009; You et al., 2016; Koua et al., 2020; Lopez et al., 2021). For example, application of biological fertilizer and dazomet in field showed that their combination could increase the number of soil microorganisms, the fertility and enzyme activities of soil, higher relative abundances of beneficial microbial groups and species, eventually leading to higher crop quality and yield (Gao et al., 2018; Zhang et al., 2021; Li et al., 2022).

It was well documented that the incidence of root-knot nematodes had significant correlation with microorganisms in soil, and the interactions between root-knot nematodes and microorganisms could be synergistic and antagonistic (Shahid et al., 2022). Therefore, optimization of soil microbial composition could affect plant growth and defense response. PGPRs or BCAs, such as *Bacillus* spp., *Pseudomonas* spp., photosynthetic bacteria, *Trichoderma* spp., had strong antagonistic effects to plant pathogens, and had been used to control plant pathogenic microorganisms (Luo et al., 2017, 2018; Su et al., 2017; Yendyo et al., 2017; Zhang et al., 2020). Dazomet and BCAs had been used together to control root-knot nematode on cucumber and bacterial wilt disease of ginger, it was found to increase soil bacterial and fungal diversities, leading to a better control effect on plant disease (Chen et al., 2019; Zhang et al., 2019b; Hu et al., 2020).

Photosynthetic bacteria are a group of common PGPRs or BCAs in natural environments, which were also known to have positive impacts on plant growth (Du et al., 2006; Hirose et al., 2012; Ferrera et al., 2017). Currently, the most common photosynthetic bacteria used in agriculture were *Rhodopseudomonas* spp., *Methylobacterium* spp., and *Sphingomonas* spp. (Nasopoulou et al., 2014; Su et al., 2017; Akter et al., 2021). *R. palustris* PSB-06 is a PGPR and/or BCAs, which had been used as microbial fertilizer, it could promote plant growth and suppress crop diseases in our earlier study (Luo et al., 2017, 2019; Zhang et al., 2020; Zhai et al., 2021) and also could increase rice plant height and increase the activities of peroxidase and superoxide dismutase to enhance rice resistance (Luo et al., 2019). In addition, the fermentation liquid of *Rhodoblastus acidophilus* PSB-01 had been shown to have strong control effect on *M. incognita*, *Pratylenchus coffeae*, *Bursaphelenchus xylophilus* through 5-Aminolevulinic acid, a secondary metabolite produced by *R. palustris* PSB-01 (Cheng et al., 2008, 2017). Though, 5-Aminolevulinic acid is also one of the secondary metabolites of *R. palustris* PSB-06, there is no report to control root-knot nematode on ginger by using microbial fertilizer of *R. palustris* PSB-06 at the present time. The preliminary results of field experiment showed that combination of dazomet and *R. palustris* PSB-06 could significantly inhibit root-knot nematode on ginger and increase ginger yield.

In this study, the field experiments were carried out to further investigate the reasons behind these beneficial functions, followed by next-generation sequencing to determine the microbial population structures in ginger root rhizosphere. Based on the results described here, the combination of *R. palustris* PSB-06 and dazomet treatment could be used to control root-knot nematode on ginger by changing soil microbial composition, physicochemical properties, and nutrient contents to benefit ginger growth and development, eventually leading to higher ginger yield.

## Materials and methods

### Test material

Ginger used in this study was a local crop that had been widely grown in this area. Photosynthetic bacterial agent *R. palustris* PSB-06, (CCTCC NO: M2012518) was from in the Key Laboratory of Integrated Management of the Pests and Diseases on Horticultural Crops in Hunan Province, Hunan Academy of Agricultural Science (HAAS). *R. palustris* PSB-06 was used at the concentration of 10^7^ cfu/g. Dazomet (CAS No. 533-74-4) was purchased from the Taian Jiangzhou Biotechnology Co., Ltd. (Taian, China) and used to fumigate soil.

### Field experiments design

Field experiment was performed at the Laiwu distract, Jinan City, Shandong Province, China (36°36′82.43″N, 117°46′80.47″E), according to the field experiment results obtained in 2019. In this distract, continuous cropping of ginger was a common phenomenon and root-knot nematode (*M. incognita*) seriously affects the quality and yield of ginger. The size of the experimental field was about 1,334 m^2^. A randomized block design with five replicates was used and each replicate contained six plots (16 m × 0.7 m each). Four treatments were: (1) *R. palustris* PSB-06 (5 kg/667 m^2^) treatment after soil fumigation with dazomet (20 kg/667 m^2^) (T1), the *R. palustris* PSB06 was applied to ginger root with irrigation on the April 18th (Germination stage), July 11th (Seedling stage) and September 8th (Vigorous growth stage), 2020, (2) soil fumigation with dazomet (20 kg/667 m^2^) (T2), (3) *R. palustris* PSB-06 (5 kg/667 m^2^) treatment (T3), the used method and times were the same as T1, and (4) soil without treatment (blank control, T4). Soil fumigation with dazomet was conducted from November 25th, 2019 to March 3rd, 2020, because these fields were only planted to ginger which had high economic benefits, and the growing period of ginger was from April 18th to October 14th, 2020. Experimental field soil was prepared using a rotary cultivator and the ginger planting spacing is 18–20 cm. Three protection lines were used for each treatment to keep consistent environmental conditions for individual treatments.

### Soil samples collection

The soil samples (about 600 g each) were collected, using a five-spot sampling method, from the four different treatments, and each sampling spot contained three locations or three plants. The samples were collected on April 18th (Germination stage), July 11th (Seedling stage), September 8th (Vigorous growth stage) and October 14th (Harvest stage), 2020. The soil samples collected from the same replicate plot were mixed as one composite sample and each mixed soil sample was divided into two parts and placed in two plastic bags (100 g each). The plastic bags were transported in a 4°C cooler to the laboratory for further analysis. Then one kind of soil samples was used to extract root-knot nematodes, the other soil samples were used to analyze soil microbial diversity and physicochemical property.

### Effect on root-knot nematode

Nematodes were extracted from collected soil samples (100 g each) using the Baermann funnel method (Whitehead and Hemming, 1965), soil samples were added to the tissue paper and placed on the funnel, filtered as water was added over the soil, incubated for 48 h and the nematodes in the flat-bottomed glass test tube were collected. The number of *M. incognita* second-stage juveniles (J2s) in each sample was determined under an inverted microscope. The root-knot nematode index and the control effect were analyzed after gingers were harvested on the October 14th, 2020. Root gall severity was assessed using a 0–5 grades accorfing to the percentage of galled roots (Grade 0 = 0%; Grade 1 = 1–20%; Grade 2 = 21–40%; Grade 3 = 41–60%; Grade 4 = 61–80%; and Grade 5 = 81–100%) as described previously (Garabedian and Van Grundy, 1983). Gall index = ∑(grade of symptom severity × plant numbers)/(total number of plants × the highest grade) × 100. The control effect is presented as (gall index of the control treatment − gall index of the treatment)/gall index of the control × 100.

### Effects on ginger growth index and leaf physiological activity index

Ginger plant height and tiller number, and leaf physiological activity index, including total chlorophyll, a content, b content, total protein, catalase (CAT) activity, malondialdehyde (MDA) content, peroxidase (POD) activity, and total superoxide dismutase (T-SOD) activity were analyzed using the samples collected at the seedling, vigorous growth, and harvest stages, respectively. For leaf physiological activity index analysis, the samples were prepared using commercial kits as instructed by the manufacturer (Nanjing Jiancheng Bioengineering Institute, China). The main stem perimeter, main stem leaf number, the width of ginger, ginger number per plant, ginger yield per plant, and shoot weight per plant were analyzed after the gingers were harvested on the October 14th, 2020.

### Soil microbial diversity and physicochemical property analyzes

A 2 g soil was taken from each collected soil subsamples and placed in a 2 ml sterile eppendorf tube. The tubes were frozen in liquid nitrogen and then, stored at −80°C prior to DNA extraction. Total DNA was extracted from individual frozen soil samples using a Fast DNA SPIN Kit as instructed (MP Biomedicals, Solon, Ohio, United States). The quality and quantity of each total DNA sample were assessed using a NanoDrop2000c spectrophotometer (Thermo Fisher Scientific, United States). The integrity of each total DNA sample was checked in 1% agarose gels through electrophoresis. The primers used to amplify the V3–V4 region in the bacterial 16S rRNA gene or the fungal ITS1 region, and other PCR amplifications were prepared as described previously (Cui et al., 2019). The bacterial and fungal libraries were sequenced on an Illumina HiSeq 2,500 platform (Illumina, San Diego, CA, United States) by the Biomarker Technologies Co, LTD (Beijing, China). The remaining soil samples collected from April 18th and October 14th, 2020, were air-dried and analyzed for total nitrogen with elemental analyzer (Vario MACRO cube, Elementar Analysensysteme GmbH, Langenselbold, Germany), hydrolytic nitrogen with burette (3.0 ml, Bomei Glass Instrument Co., Ltd., Beijing, China), total phosphorus and available phosphorus with spectrophotometer (723 N, Xianke spectrometer Co., Ltd., Shanghai, China), total potassium and available potassium with flame photometer (M410, Sherwood Scientific Ltd., Cambridge, United Kingdom), pH values with pH meter (S220-K, Mettler-Toledo GmbH, Greifensee, Switzerland), organic matters with soil organic matter analyzer (TS-8200, Yile Intelligent Instrument Co., Ltd., Shanghai, China) by the Agricultural Chemical Testing Center at the HAAS.

### Statistical and bioinformatics analysis

All experiments were performed with five biological replicates. Statistical analyzes of ginger growth indices and disease severities were performed using the Analysis of Variance (ANOVA) and the SPSS 13.0 statistical software package (SPSS, Inc., Chicago, IL, United States). The statistical significance among the treatments was determined using the Duncan’s Multiple Range Test (DMRT) at the 5% level. The bioinformatics analysis of this study was performed with the aid of the BMK Cloud,^1^ the raw data were primarily filtered and processed for quality control to generate high-quality reads. The high-quality reads with similarity ≥ 97% were clustered into the same operational taxonomic unit (OTU). Taxonomic classification was processed following feature analysis, generating species abundance at levels of phylum, class, order, family, genus and species, as well as taxonomic tree and phylogenetic tree at genus level. The microbial alpha diversity of each sample were calculated at 97% similarity level and generated dilution curve and rank abundance curve, including Chao1, Ace, Shannon, Simpson, Coverage, PD_whole_tree indices, varied widely across locations, growth stage, and cultivation type for both bacteria and fungi. Study on differences of species diversity between samples was conducted with beta diversity analysis, nonmetric multidimensional scaling (NMDS), principal coordinate analysis (PCoA) and boxplot plot based on multiple distances were obtained. In addition, differential biomarkers with statistical significance were identified between different treatments and growth stages and interaction between microbial community and environmental factors were studied based on correlation analysis. All sequencing data were deposited in NCBI and can be accessed in the Sequence Read Archive (SRA) database^2^ under the accession numbers SRR21473349 to SRR21473428, BioSample accession numbers SAMN30697183 to SAMN30697262, and the BioProject accession number PRJNA877365.

## Results

### Effect of *Rhodopseudomonas palustris* PSB-06 and dazomet on root-knot nematode on ginger

**Figure 1:**
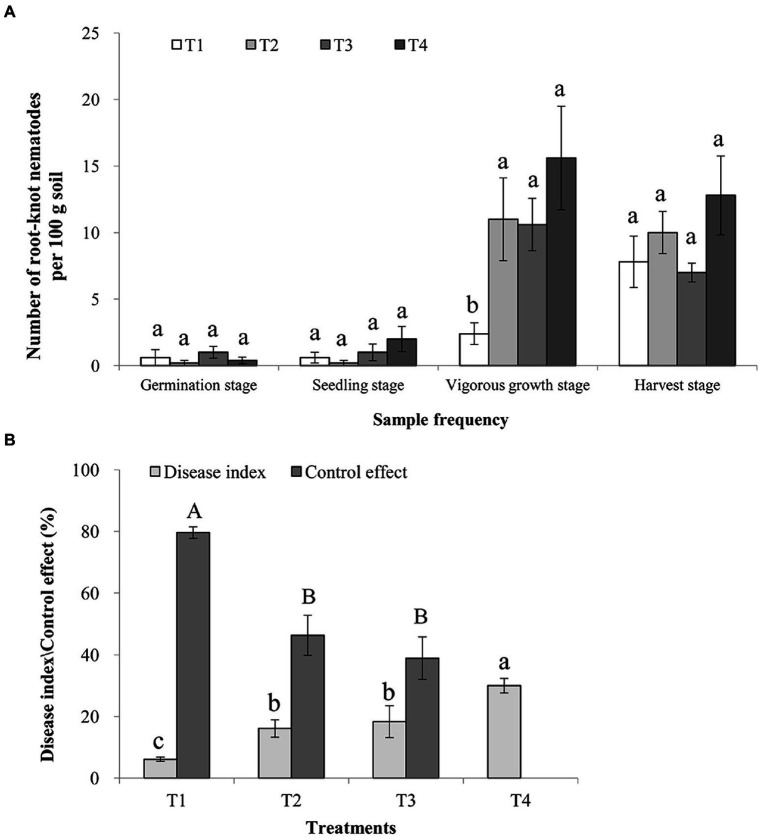
Control effects of *Rhodopseudomonas palustris* PSB-06 and dazomet on root-knot nematode. **(A)** Numbers of root-knot nematodes in various soil samples. Each soil sample was 100 g. **(B)** Disease indices caused by root-knot nematode and the control effects of the three treatments. T1, soil was treated with *R. palustris* PSB-06 and dazomet; T2, soil was treated with dazomet alone; T3, soil was treated with *R. palustris* PSB-06 alone; T4, soil without treatment (blank control). Same low or up case letters indicate no significant statistical difference (*p* > 0.05) among the treatments, determined using the Duncan’s multiple range test.

Root-knot nematode J2s population in all treated soil samples collected at the germination and seedling stages was low and similar (*p* > 0.05). The sizes of J2s populations in these four treatments soil samples were then, significantly increased at the vigorous growth and harvest stages. However, the sizes of J2s population in the T1 treatment soil were significantly lower than those in the T2, T3, and T4 treatments soils at the vigorous growth stage (*p* < 0.05; Figure 1A). Consistently, the symptoms index caused by root-knot nematode in the T1 treatment was significantly lower (*p* < 0.05) than those in the T2 and T3 treatments soils at the harvest stage (*p* < 0.05). The control effect of the T1 treatment was 80.00 ± 10.18%, significantly higher than those of the T2 (46.67 ± 13.33%) and T3 (40.00 ± 15.56%) treatments (*p* < 0.05; Figure 1B).

### Effect of combination of *Rhodopseudomonas palustris* PSB-06 and dazomet on ginger growth

The plant height, tiller number, main stem perimeter, yield of ginger and the ginger ball width collected from the T1 treatment at the harvest stage were significantly greater (*p* < 0.05) than those of the T4 treatment (Table 1). It was shown that T1 treatment could improve ginger growth and yields by 9.68 and 37.37% compared to T2 and T4 treatments, respectively. But compared to the T2 and T3 treatments, there were no significant statistical difference except for the plant height (*p* > 0.05). The number of main stem leaves and the ginger number per plant were similar among the four treatments at the harvest stage. Whereas, the fresh shoot weight and main stem perimeter of the plants collected from the T3 treatment were significantly greater (*p* < 0.05) than that of the plants collected from the T4 treatment, but were similar to that of the plants collected from the T1 and T2 treatments.

**Table 1:** The growth indices of ginger at harvest stage.

Treatments	Plant height (cm)	Tiller number	Main stem perimeter (cm)	Main stem leaf number	Ginger number per plant	Ginger ball width (cm)	Shoot fresh weight per plant (kg)	Ginger yield per plant (kg)
T1	101.90 ± 2.06a	10.00 ± 0.0.58a	5.58 ± 0.14a	23.30 ± 0.82a	13.30 ± 1.27a	31.73 ± 1.51a	0.79 ± 0.08ab	1.36 ± 0.11a
T2	92.40 ± 3.06b	9.20 ± 0.65ab	5.20 ± 0.14a	22.80 ± 0.81a	12.10 ± 1.05a	29.33 ± 1.32ab	0.78 ± 0.08ab	1.24 ± 0.09ab
T3	91.60 ± 2.78b	9.20 ± 0.53ab	5.30 ± 0.16a	23.20 ± 1.04a	12.10 ± 1.38a	30.20 ± 1.15ab	0.82 ± 0.08a	1.26 ± 0.10ab
T4	87.40 ± 3.80b	8.20 ± 0.39b	4.73 ± 0.13b	21.50 ± 1.00a	10.00 ± 1.05a	26.48 ± 1.13b	0.57 ± 0.05b	0.99 ± 0.10b

T1, soil treated with *R. palustris* PSB-06 and dazomet; T2, soil treated with dazomet alone; T3, soil treated with *R. palustris* PSB-06 alone; T4, soil without treatment (blank control). Same low case letters indicate no significant statistical difference (*p* > 0.05) among the treatments, determined using the Duncan’s multiple range test.

### Treatment using *Rhodopseudomonas palustris* PSB-06 and dazomet increased chlorophyll and total protein contents in ginger leaves

**Figure 2:**
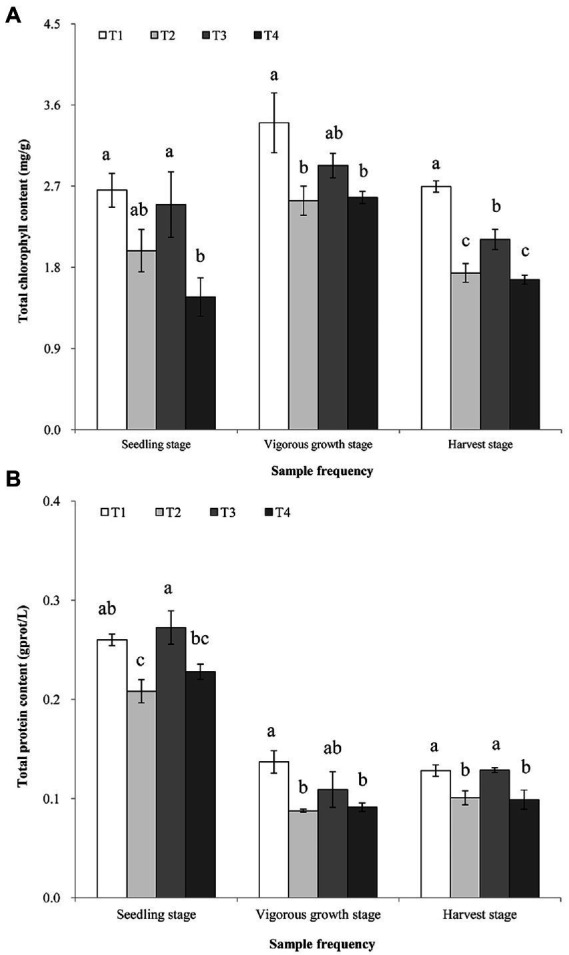
Analyzes of total chlorophyll **(A)**, and total protein **(B)**, contents in the ginger plants harvested at the three stages. T1, soil treated with *R. palustris* PSB-06 and dazomet; T2, soil treated with dazomet alone; T3, soil treated with *R. palustris* PSB-06 alone; T4, soil without treatment (blank control). Same low case letters indicate no significant statistical difference (*p* > 0.05) between the treatments, determined using the Duncan’s multiple range test.

The results showed that the total chlorophyll contents in the ginger leaves collected from the T1 treatment at the three sampling stages were all greater than those in the leaf samples collected from the T2-T4 treatments at the three sampling stages (Figure 2). At the vigorous growth and harvest stages, the total chlorophyll contents of T1 and T3 treatments were significantly greater than that in T4 treatment (Figure 2A). The total protein contents in the leaf samples collected from the T1 treatment at the vigorous growth and harvest stages were all greater than that in leaf samples collected from the T4 treatment, but the total protein content was similar between T1 and T3 treatments samples at three stages (Figure 2B). No significant statistical differences (*p* > 0.05) were observed on the total chlorophyll and protein contents in the leaf samples collected from the T2 and T4 treatments at the three growth stages. The chlorophyll a and b in the leaf samples showed that their changes in them in the leaf samples were similar to that observed for the total chlorophyll contents (Supplementary Figures S1A, B in Supplementary File S1). The full details of analysis of chlorophyll a and b were shown in the Supplementary Figures S1A, B and the Result S1 in Supplementary File S1.

Analyzes of CAT, POD and T-SOD activities and MDA content (Supplementary Figure S1 in Supplementary File S1) in the leaf samples collected from the four treatments and at the three growth stages showed that no significant statistical differences (*p* > 0.05) were observed of the CAT activities in the leaf samples collected from the four treatments at the three growth stages (Supplementary Figure S1C in Supplementary File S1). The MDA content increased with the growth of ginger, it indicated the active oxygen in ginger leaves increased, but no significant statistical differences (*p* > 0.05) in four treatments (Supplementary Figure S1D in Supplementary File S1). At the harvest stage, the POD activity in the leaf sample collected from the T1 treatment was significantly lower (*p* < 0.05) than T4 treatment, and the T-SOD activities in the leaf samples collected from the T2 and T4 treatments were significantly lower (*p* < 0.05) than T1 and T3 treatments (Supplementary Figures S1E, F in Supplementary File S1).

### Effect of different treatments and growth stages on microbiota composition

**Figure 3:**
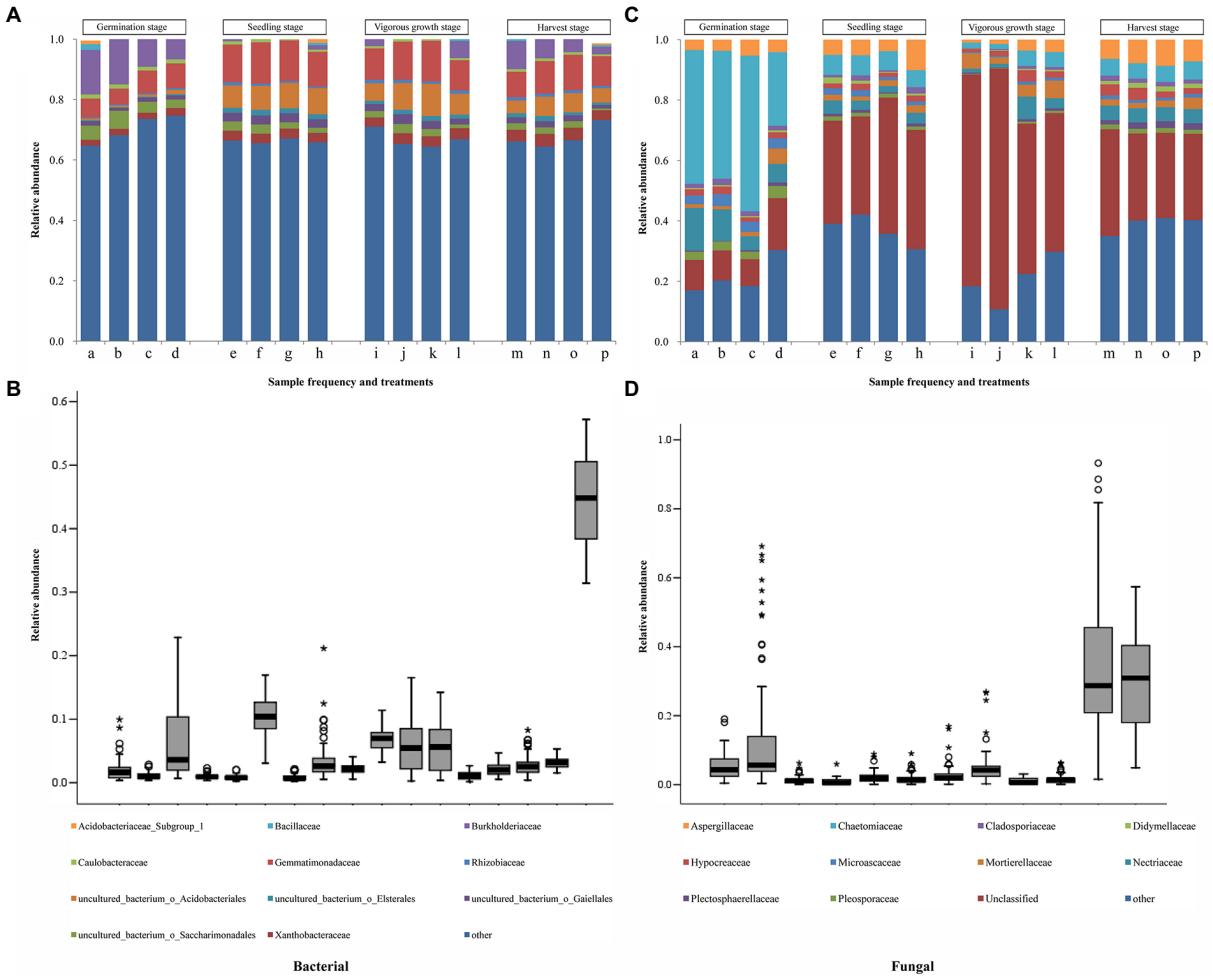
Taxonomic structures of soil bacterial and fungal populations at the family levels. **(A)** and **(C)** Taxonomic structures of soil bacterial and fungal populations. a, e, i, and m represent the soil samples treated with *R. palustris* PSB-06 and dazomet. b, f, j, and n represent the soil samples treated with dazomet alone. c, g, k, and o represent the soil samples treated with *R. palustris* PSB-06 alone. d, h, l, and p represent the soil samples without treatment (blank control). Only the 16 bacterial families **(A)**, and the 11 fungal families **(C)**, with the highest mean relative abundance were shown. **(B)** Boxes showing the 16 bacterial families with the largest mean relative abundance. **(D)** Boxes showing the 11 fungal families with the largest mean relative abundance. The other bacterial and fungal families were all classified as “other.” The median value of a family was represented as a line in the box. The interquartile ranges covered the 75th to 25th percentiles. The whiskers were the inclusive of the maximal and minimal values without the outliers. The cycles and stars indicate the discrete and extreme values, respectively.

The full details of sequences assembly and species annotation were shown in the Results S3, S4 in Supplementary File S1 (Supplementary Figures S2, S3; Supplementary Tables S1–S9 in Supplementary File S1). The dominant bacterial and fungal phyla found in the four treatments samples were Proteobacteria and Ascomycota, respectively (Supplementary Tables S10, S11 in Supplementary File S1). At the germination stage, Sphingobacteriaceae and Chaetomiaceae were the dominant bacterial and fungal family found in the four treatments samples, respectively; whereas, at the other three growth stages, Gemmatimonadaceae and Unclassified family were the dominant bacterial and fungal family found in the four treatments samples, respectively (Figure 3; Supplementary Tables S12, S13 in Supplementary File S1). A total of 16 bacterial families out of the top 50 families, and 11 fungal families out of the top 30 families, were found to be common in the samples collected from different growth stages (Figure 3). Significant difference analysis (*p* < 0.05) of the top 16 common bacterial and 11 common fungal families from different developmental stages were shown in Supplementary Tables S16, S17 in Supplementary File S1. There were 12 bacterial families showing significant differences (*p* < 0.05) among the four treatments at the harvest stage, but only 5 fungal families showing significant differences (*p* < 0.05) among the four treatments. In results in Table 3 showed that a total of 8 bacterial and 6 fungal families shared among both them, these common species had great differences at the different treatments and growth stages (Supplementary Tables S14, S15 in Supplementary File S1). The quantity and variety of microorganisms changed as ginger growth progressed. At the bacterial family level, the relative abundances of Rhodanobacteraceae and uncultured bacterium Saccharimonadales from the T1 treatments were significantly decreased (*p* < 0.05) than T4 treatments at the four growth stages. At the germination and harvest stages, the relative abundances of Burkholderiaceae and Xanthobacteraceae had significant differences (*p* < 0.05) among the four treatments. At the vigorous growth and harvest stages, the relative abundances of uncultured bacterium Gaiellales, Gemmatimonadaceae and Sphingomonadaceae had significant differences (*p* < 0.05) among the four treatments. For the fungal families, the relative abundances of Chaetomiaceae, Mortierellaceae and Nectriaceae was significant difference (*p* < 0.05) among the four treatments at the germination stage, no significant statistical differences (*p* > 0.05) at the harvest stage, but the Mortierellaceae showed significant difference (*p* < 0.05) at the harvest stage. The results of top 20 bacteria and fungi at the genus level showed that the dominant genera were different in the different treatments (Supplementary Tables S16, S17 in Supplementary File S1). At the harvest stage, 4, 12 and 15 different dominant genera were in the T2, T3 and T4 treatments comparing to the T1 treatment, and the Chujaibacter and Chloroplast were the unique genera in the T1 treatment. The full details of these sections were shown in the Results S3, S4 in Supplementary File S1.

**Table 2:** The Shannon-Wiener and Simpson’s diversity indices of soil microorganisms at different developmental stages.

Sample frequency	Treatments	Bacteria		Fungi	
		Shannon	Simpson	Shannon	Simpson
Germination stage	A	7.2540cd	0.9768d	4.3875f	0.8570bc
	B	7.1270d	0.9768d	4.6084ef	0.8791bc
	C	7.5909c	0.9817cd	4.1635f	0.8172c
	D	8.1003b	0.9861bc	4.9761def	0.9355ab
Seedling stage	E	8.6465a	0.9937a	6.3234abc	0.9572ab
	F	8.6028a	0.9934a	6.4544abc	0.9501ab
	G	8.6282a	0.9932a	5.8379 cd	0.9430ab
	H	8.6488a	0.9930a	5.8926 cd	0.9079abc
Vigorous growth stage	I	8.4741ab	0.9917ab	4.2840f	0.8589bc
	J	8.5009ab	0.9922a	4.5483ef	0.8879abc
	K	8.4697ab	0.9915ab	6.1347bcd	0.9260ab
	L	8.4271ab	0.9894ab	5.5939cde	0.8871abc
Harvest stage	M	8.2520ab	0.9892ab	7.1943ab	0.9824a
	N	8.3672ab	0.9912ab	7.2627ab	0.9827a
	O	8.6466a	0.9923a	7.3967a	0.9847a
	P	8.6724a	0.9916ab	7.3498a	0.9838a

A, E, I, and M are soils treated with *R. palustris* PSB-06 and dazomet (T1). B, F, J, and N are soils treated with dazomet alone (T2). C, G, K, and O are soils treated with *R. palustris* PSB-06 alone (T3). D, H, L, and P are soils without treatment (blank control, T4). Same low case letters indicate no significant statistical difference (p>0.05) among the treatments, determined using the Duncan’s multiple range test.” after “D, H, L, and P are soils without treatment (blank control, T4).

**Table 3:** Significant difference analysis (*p* < 0.05) of the top 16 common bacterial and 11 common fungal families from different developmental stages and four developmental stages.

	Family	Germination stage				Seedling stage				Vigorous growth stage				Harvest stage			
		T1	T2	T3	T4	T1	T2	T3	T4	T1	T2	T3	T4	T1	T2	T3	T4
Bacteria	Burkholderiaceae	0.15ab	0.17a	0.13ab	0.10b	0.02a	0.02ab	0.01b	0.01ab	0.05a	0.04a	0.03a	0.06a	0.09a	0.07ab	0.05ab	0.03b
	Gaiellales	0.02a	0.01a	0.01a	0.01a	0.03a	0.03a	0.03a	0.03a	0.02b	0.03a	0.03ab	0.02c	0.02ab	0.02a	0.02ab	0.01b
	Gemmatimonadaceae	0.06a	0.05a	0.07a	0.08a	0.13a	0.14a	0.13a	0.12a	0.10bc	0.13ab	0.13a	0.10c	0.08b	0.11ab	0.12a	0.10ab
	Rhodanobacteraceae	0.05a	0.04a	0.02b	0.01b	0.03a	0.03a	0.02ab	0.02b	0.09a	0.03ab	0.02b	0.02b	0.07a	0.04ab	0.04ab	0.01b
	Saccharimonadales	0.05ab	0.06a	0.04ab	0.03b	0.03a	0.03a	0.02ab	0.02b	0.02b	0.03a	0.02ab	0.01c	0.02a	0.02a	0.02a	0.01b
	Solibacteraceae	0.01b	0.01b	0.01b	0.02a	0.02a	0.02a	0.02a	0.02a	0.02a	0.02a	0.03a	0.03a	0.02a	0.03a	0.03a	0.03a
	Sphingomonadaceae	0.07a	0.05a	0.08a	0.09a	0.07a	0.08a	0.07a	0.06a	0.08a	0.07ab	0.05b	0.06b	0.06ab	0.05b	0.07a	0.08a
	Xanthobacteraceae	0.02b	0.02b	0.02b	0.03a	0.03a	0.03a	0.03a	0.03a	0.03a	0.04a	0.03a	0.04a	0.04ab	0.04a	0.04a	0.03b
Fungi	Aspergillaceae	0.03a	0.04a	0.05a	0.04a	0.05a	0.05a	0.04a	0.10a	0.01c	0.01bc	0.04ab	0.04a	0.06b	0.08ab	0.09a	0.07ab
	Chaetomiaceae	0.44ab	0.42ab	0.52a	0.24b	0.07a	0.07a	0.06a	0.06a	0.02a	0.02a	0.05a	0.05a	0.06a	0.05a	0.05a	0.06a
	Hypocreaceae	0.02a	0.02a	0.01a	0.02a	0.02a	0.02a	0.01a	0.02a	0.01a	0.02a	0.04a	0.02a	0.04a	0.04a	0.02a	0.02a
	Mortierellaceae	0.01b	0.01b	0.01b	0.05a	0.02a	0.01a	0.02a	0.03a	0.05a	0.02a	0.04a	0.06a	0.02b	0.02b	0.02b	0.04a
	Nectriaceae	0.14a	0.10ab	0.05b	0.06ab	0.04a	0.03a	0.02a	0.04a	0.01a	0.01a	0.07a	0.03a	0.05a	0.05a	0.05a	0.05a
	Unclassified	0.10a	0.10a	0.09a	0.17a	0.34a	0.32a	0.45a	0.40a	0.70ab	0.80a	0.50b	0.46b	0.35a	0.29a	0.28a	0.29a

Same low case letters indicate no significant statistical difference (*p* > 0.05) between the treatments, determined using the Duncan’s multiple range test.

### Effect of different treatments and growth stages on bacterial and fungal alpha diversity

Microbial alpha diversity varied widely among different treatments and growth stages. For microbial-community structure, the Shannon and Simpson indices indicated the bacterial and fungal communities significantly differed among different treatments (Table 2; Supplementary Figures S4, S5 in Supplementary File S1). The bacterial and fungal diversities in the soils treated with dazomet (T1 and T2) at the germination stage were lower than that the soils without dazomet (T3 and T4). The bacterial diversity increased significantly (*p* < 0.05) as the growth stage progressed, especially in soils without dazomet treatment. Application of *R. palustris* PSB-06 (T1 and T3) had a considerable rise in bacterial diversity than the other treatments, no significant statistical differences (*p* > 0.05) were observed among the samples collected at the seedling, vigorous growth, and harvest stages. The fungal diversities significantly increased (*p* < 0.05) at the seedling stage, but it has a considerable decline at vigorous growth stage and considerable rise at the harvest stage again. The fungal diversity at the harvest stage was greater than those found in the samples collected at the other three growth stages, the highest Shannon and Simpson indices were all found in the T3 treatment sample collected at the harvest stage, and no significant statistical differences (*p* > 0.05) were observed among the samples. The full details of Shannon and Simpson indices in different growth stages or treatments were shown in the Result S5 in Supplementary File S1.

### Effect of *Rhodopseudomonas palustris* PSB-06 on probiotic bacteria and soil-borne pathogenic fungi

**Figure 4:**
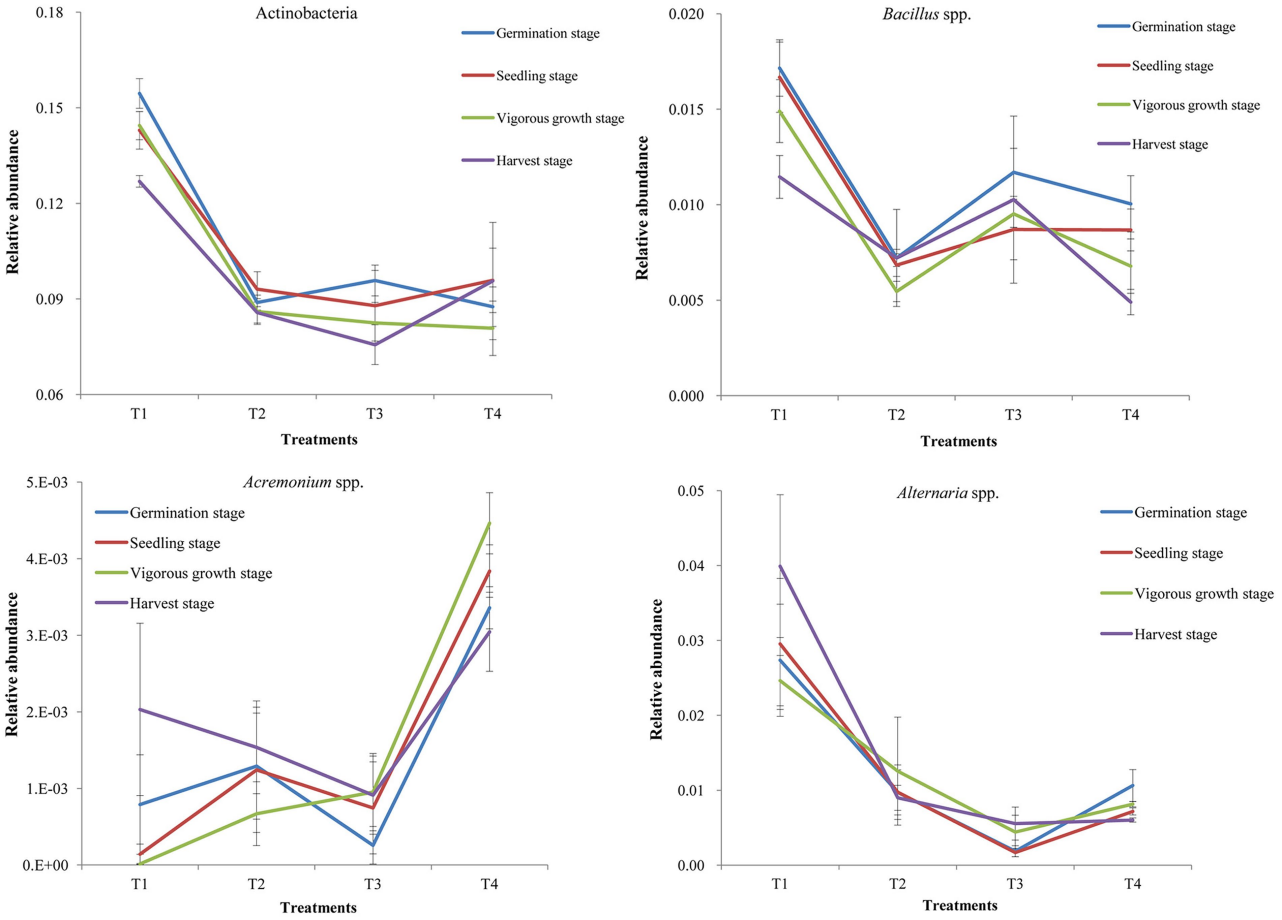
Relative abundance of probiotic bacterial Actinobacteria and *Bacillus* spp., and pathogenic fungal *Acremonium* spp. and *Alternaria* spp. in the samples collected at different growth stages. T1, soil treated with *R. palustris* PSB-06 and dazomet; T2, soil treated with dazomet alone; T3, soil treated with *R. palustris* PSB-06 alone; T4, soil without treatment (blank control). The statistical differences between the treatments were determined using the Duncan’s Multiple Range Test (DMRT) at the 5% level.

The diversities and community compositions of several soil probiotic bacteria and soil-borne pathogenic fungi were analyzed in this study (Figure 4). The results showed that the diversities and community compositions of probiotic bacterial Actinobacteria, *Bacillus* spp., and *Paenibacillus* spp. in the T1 and T3 treatment samples were greater than that of the T2 and T4 treatment samples at different growth stages (Figure 4; Supplementary Figure S6 in Supplementary File S1). The diversities and community compositions of soil pathogenic bacterial *Stenotrophomonas* spp., and *Ralstonia* spp. were greater in the T4 treatment samples than those of other treatment samples, while the diversity and community composition of soil pathogenic bacteria in the T1 treatment sample were smaller than those of the T2 and T3 treatment samples (Supplementary Figure S6 in Supplementary File S1). Among the assayed fungi, the diversity and community composition of *Fusarium* spp. were greater in the T1 and T3 treatment samples. The diversity and community composition of *Alternaria* spp. in the T1 treatment samples were the greatest, while that of the T3 treatment samples were the smallest (Figure 4; Supplementary Figure S6 in Supplementary File S1). The diversity and community composition of *Acremonium* spp. in the T1 and T3 treatment samples were smaller than that of the other two treatment samples.

### Effect of application of *Rhodopseudomonas palustris* PSB-06 on nutrients in soil

Compared to the samples collected from the T1 and T2 treatments at the harvest stage, the pH values of the T3 and T4 samples were remarkably increased (Supplementary Figure S7A in Supplementary File S1), suggesting that using dazomet would reduce the number of alkali producing microorganisms in the soil. The contents of organic matter, total nitrogen, hydrolytic nitrogen, phosphorus, and available phosphorus besides potassium content in the soil samples collected from the T1 and T2 treatments were lower at the germination stage. Whereas at the harvest stage, the contents of organic matter, hydrolytic nitrogen, available phosphorus and available potassium were higher in T1 and T3 than in T2 and T4 treatments. The full details of analysis of these sections were shown in the Result S2 in Supplementary File S1.

### Relationship between soil microbial community composition and physicochemical properties

**Figure 5:**
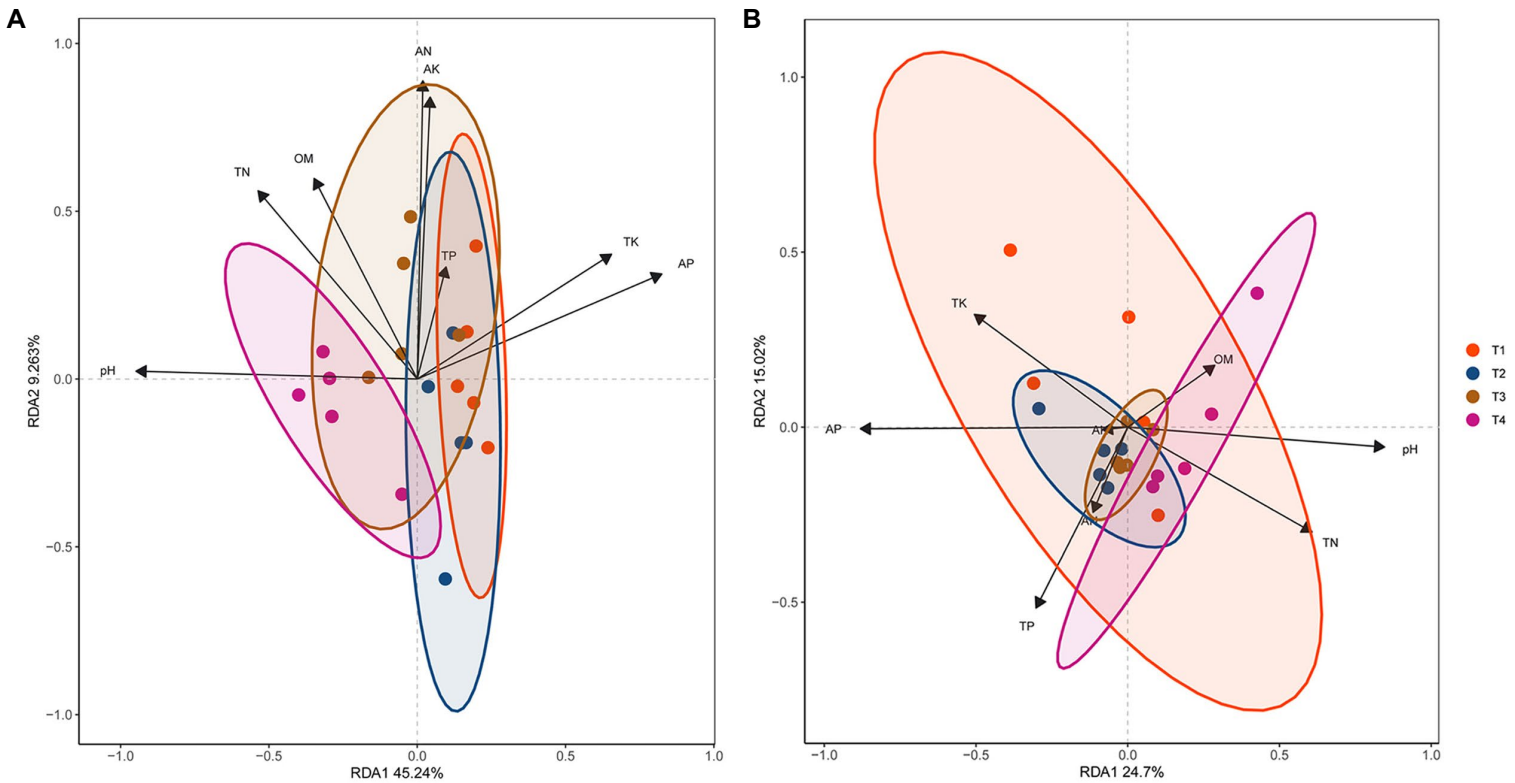
Redundancy analysis (RDA) of relative abundances of dominant bacterial and fungal phyla and the physicochemical properties in soil collected at the harvest stage. RDA analysis was performed using the Tutools platform (https://www.cloudtutu.com) at 95% confidence interval. The arrow length represents the strength of the correlation between the soil microorganisms and physicochemical properties. Longer arrows indicate stronger correlations. The perpendicular distance between the soil microorganisms and physicochemical properties axes reflects their correlations, and the smaller distance indicates a stronger correlation. T1, soil treated with *R. palustris* PSB-06 and dazomet. T2, soil treated with dazomet alone. T3, soil treated with *R. palustris* PSB-06 alone. T4, soil without treatment (blank control). pH, potential of hydrogen; OM, organic matter; TN, total nitrogen; AN, hydrolytic nitrogen; TP, total phosphorus; AP, available phosphorus; TK, total potassium; and AK, available potassium.

The principal coordinates analysis (PCoA) and non-MetricMulti-Dimensional Scaling (NMDS) results indicated that the bacterial and fungal populations in the T4 treatment sample were all clearly different from those of the other three treatment samples (Supplementary Figure S8 in Supplementary File S1). The distributions of bacterial and fungal population points were reversed compared to the points obtained through PCoA analysis. The redundancy analysis (RDA) results showed that the bacterial and fungal communities were mostly clustered (Figure 5; Supplementary Table S18 in Supplementary File S1). The RDA1 and RDA2 values accounted for 45.24 and 9.26% of the total variations of the bacterial communities, respectively (Figure 5A). The correlation between the available phosphorus and Dependentiae, Patescibacteria or Rokubacteria, and the correlation between the pH value and Dependentiae Firmicutes, Patescibacteria or Rokubacteria were all significantly positive (*p* < 0.01), indicating that Dependentiae, Patescibacteria and Rokubacteria were closely correlated with the levels of soil pH value and available phosphorus (Supplementary Table S19 in Supplementary File S1). The RDA1 and RDA2 values accounted for 24.70 and 15.02% of the total fungal variations, respectively (Figure 5B). Significant positive correlations (*p* < 0.01) were observed between the available phosphorus and Basidiomycota, Mucoromycota or Ascomycota, and the pH value and Mucoromycota (Supplementary Table S20 in Supplementary File S1), indicating that Mucoromycota was also closely correlated to the levels of pH value and available phosphorus. The full details of these sections were shown in the Result S6 in Supplementary File S1.

**Figure 6:**
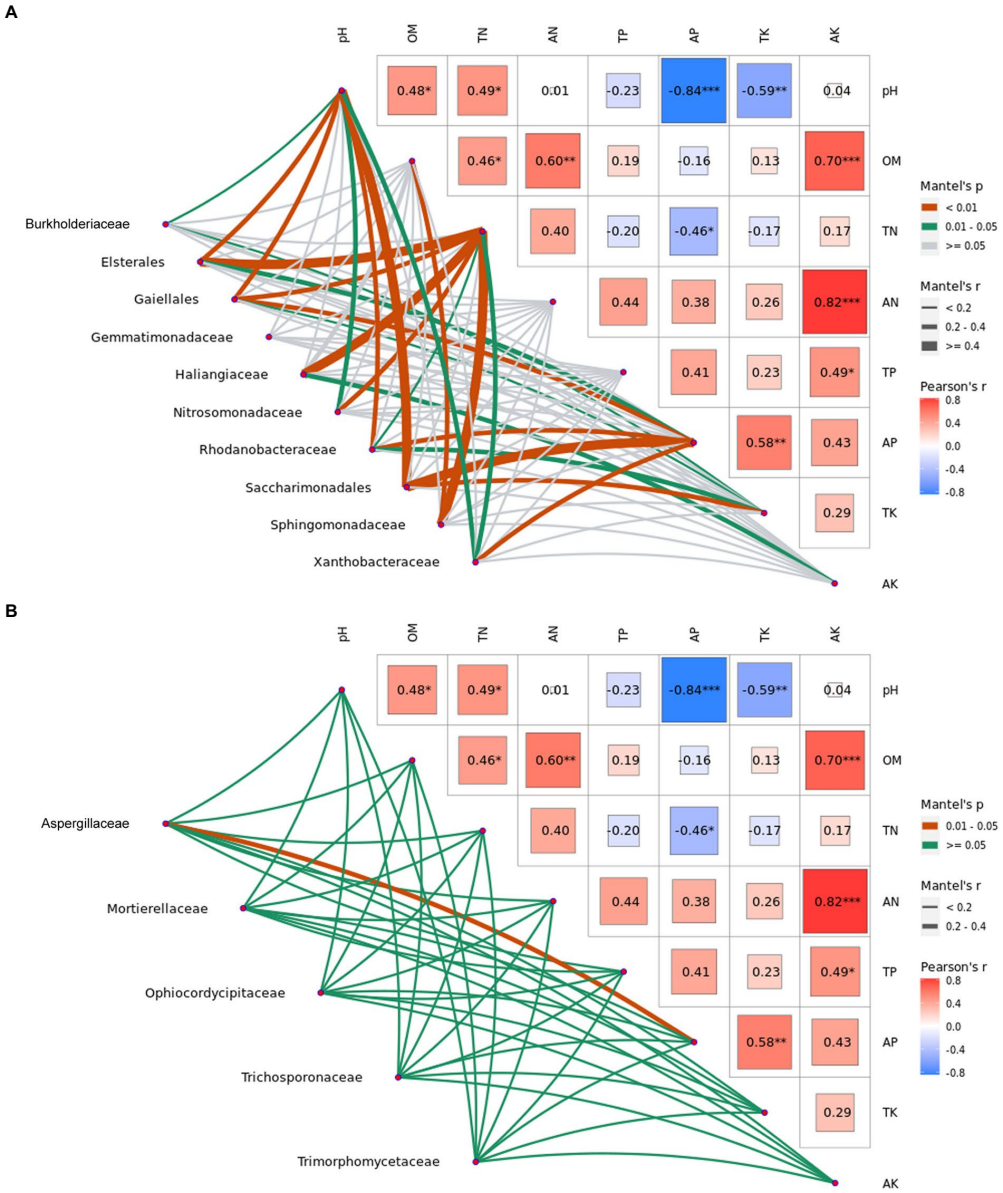
Mantel test of abundances of dominant bacterial and fungal family with significant difference analysis (*p* < 0.05) at the harvest stages and the physicochemical properties in ginger soil. Elsterales, Gaiellales and Saccharimonadales are the uncultured bacterium. pH, potential of hydrogen; OM, organic matter; TN, total nitrogen; AN, hydrolytic nitrogen; TP, total phosphorus; AP, available phosphorus; TK, total potassium; and AK, available potassium.

In addition, the top 10 common bacterial and 5 common fungal families with significant differences analysis (*p* < 0.05) at the harvest stages were also used to analyze the correlation with physicochemical properties of ginger soil (Figure 6). It was indicated that significant positive correlations (*p* < 0.01) were found between the bacterial family and the soil physicochemical properties (Figure 6A; Supplementary Table S21 in Supplementary File S1). The correlation between the pH value and uncultured bacterium, Elsterales, uncultured bacterium, Gaiellales, Rhodanobacteraceae or uncultured bacterium Saccharimonadales, and the correlation between the total nitrogen and uncultured bacterium, Elsterales, uncultured bacterium, Gaiellales, Haliangiaceae or Sphingomonadaceae, and the correlation between the available phosphorus and uncultured bacterium Gaiellales, Rhodanobacteraceae, uncultured bacterium Saccharimonadales or Xanthobacteraceae, and the correlation between the total potassium and uncultured bacterium Saccharimonadales, and the correlation between the organic matter and Sphingomonadaceae were all significantly positive (*p* < 0.01), indicating that the bacterial family and the soil physicochemical properties were closely correlated. The correlation between the fungal family and the soil physicochemical properties showed that significant negative correlations (*p* < 0.05) were only found between Aspergillaceae and the available phosphorus, indicating that the fungal family and the soil physicochemical properties were not closely correlated compared with the bacterial family (Figure 6A; Supplementary Table S22 in Supplementary File S1).

## Discussion

In this study, the results showed that the combination of *R. palustris* PSB-06 and dazomet was an effective method to control root-knot nematode disease and improve ginger soil conditions. The root-knot nematode J2s population was lower during the germination and seedling stages, then increased significantly at the vigorous growth and harvest stages. It might because Laiwu distract is located in northern China and the environmental temperature was still lower at the early stage of ginger, the reproduction of J2s population was slow and it increased slowly. As environmental temperature raises for summer, the sizes of J2s population increased rapidly. On the other hand, soil treated with dazomet also could reduce the initial J2s population, but with the rapid degradation of dazomet, recovery of J2s population increased significantly. In the previous report, the control effect of dazomet fumigation on nematode populations till 12 weeks (Eo and Park, 2014), the results in this study showed that the control effect of *R. palustris* PSB-06 and dazomet treatments on root-knot nematode infecting ginger contiued 25 weeks and the sizes of J2s populations remained significantly lower compared to that in the soil without treatment or dazomet alone. It was suggested that the combination of *R. palustris* PSB-06 and dazomet treatments could effectively control root-knot nematode, and also reflected that the potential of *R. palustris* PSB-06 for controlling root-knot nematode in field.

On the other hand, after *R. palustri* PSB-06 treatment, the soil bacterial diversity rapidly increased and then, maintained at the similar levels throughout the ginger growth season. In the *R. palustri*s PSB-06-treated soils, the most dominant bacterial Phylum was Proteobacteria, the second dominant Phylum was Acidobacteria and the dominant bacterial family was Gemmatimonadaceae. The phylum Acidobacteria could cause a wide range of breakdowns, utilization and biosynthesis of different carbohydrates, and decomposing of various biopolymers (Dedysh and Yilmaz, 2018). Bacteria in Gemmatimonadaceae are crucial for phosphate dissolution, microbial nitrogen metabolism and soil respiration (Takaichi et al., 2010; Lu et al., 2020). In addition, it showed that compared to the non-treated soil samples, the soil samples treated with *R. palustris* PSB-06 had higher levels of Actinobacteria, *Bacillus* spp., and *Paenibacillus* spp., and lower levels of *Stenotrophomonas* spp., and *Ralstonia* spp. It indicated that *R. palustris* PSB-06 could increase diversity and community composition of probiotic bacteria while decrease that of soil-borne pathogenic fungi. Moreover, it was noteworthy that a large portion of sequences in our microbiome data remained uncharacterized. Consequently, further studies are necessary to better understand the diversity of plant rhizosphere microorganisms.

Soil physicochemical properties were closely related to microbial community structure and diversity, and affect the crop growth and yield (Paul and Lade, 2014; Zhou et al., 2017). The results of analyzed soil physicochemical properties showed that combination of *R. palustris* PSB-06 and dazomet treatment could prevent soil pH value changing during ginger growth, and *R. palustris* PSB-06 treatment could increase the content of soil organic matters. After the vigorous growth stage, the microbial community structure significantly changed, especially at the harvest stage. Analysis of RDA showed that the structures of bacterial and fungal communities were all significantly positively correlated with soil physicochemical properties at the harvest stage. Dependentiae, Patescibacteria, and Rokubacteria in the dominant bacterial phyla and Mucoromycota in the dominant fungal phylum were closely correlated with the soil pH value and the content of available phosphorus. It is well known that soil pH is an important factor that could affect microbial community structure and diversity in plant rhizosphere, it was found to be negatively correlated with the relative abundance of Gemmatimonadetes after treatment with *R. palustris* PSB-06 (Zhou et al., 2017; Luo et al., 2019). Microbial species richness and phylogenetic diversity of the archaeal communities were significantly and negatively correlated with the content of available phosphorus (Zhang et al., 2019a). However, the results in this study showed that dominant bacterial and fungal phyla were positivity correlated with available phosphorus, and did not support soil pH was negatively correlated with the relative abundance of Gemmatimonadetes (Luo et al., 2019), it might because the microorganisms of concern and the crops used in this study were different, and the molecular mechanism of their correlation would need further studies.

The correlation of common bacterial and fungal families with physicochemical properties of ginger soil showed that the dominant families in Proteobacteria, including uncultured bacterium, Elsterales, Haliangiaceae, Rhodanobacteraceae, Sphingomonadaceae and Xanthobacteraceae were all not positive correlated with the total potassium, but were significantly positively correlated with the other soil physicochemical properties. However, in the dominant bacterial phylum, Proteobacteria was only significantly positive correlated with the total potassium. Elsterales was positively correlated with soil pH, but negatively correlated with soil NH_4_^+^-N and NO_3_^−^-N contents by studying the effects of long-term N deposition on soil bacterial and fungal abundance (Wang et al., 2021), uncultured bacterium, Elsterales and soil pH were higher in soil treated with dazomet than the soil without dazomet, indicating that soil pH and dazomet had a synergetic effect on the diversity of uncultured bacterium, Elsterales. A few of dominant microbes, Sphingomonadaceae in core community was still tightly correlated with physiochemical properties according to correlation analysis (Zhu et al., 2021), Sphingomonadaceae was also significantly positive correlated with physiochemical properties except for total potassium in this study. In the dominant fungal family, only Aspergillaceae was significantly negative correlated with the available phosphorus, but the Ascomycota was significantly positive correlated with the pH value and available phosphorus.

The results of ginger yield showed that combination of *R. palustris* PSB-06 and dazomet treatments could improve ginger growth and increased yields by 9.68 and 37.37% compared to dazomet treatment only (T2) and blank control (T4), respectively. The total of chlorophyll and protein contents in the ginger leaves collected from the *R. palustris* PSB-06 treatments (T1 and T3) were all greater than the other treatments at the different growth stages. They indicated that *R. palustris* PSB-06 had positive effect on the content of chlorophyll and total protein in the ginger leaves and there appeared a strong possibility that increasing ginger yields were caused by increasing chlorophyll and total protein contents to enhance photosynthetic activities. The previous studies also showed that *R. palustris* PSB-06 could promote plant growth (Luo et al., 2017, 2019; Zhang et al., 2020; Zhai et al., 2021). Although the POD content was not changed significantly at the earlier growth stages, the POD content in combination of *R. palustris* PSB-06 and dazomet treatment was much lower than the other treatments at the vigorous growth and the harvest stages. The activities of T-SOD and POD contents in the leaves were increased under stress conditions or late stage of plant growth (Myouga et al., 2008). It was indicated that *R. palustris* PSB-06 treatment could delay the senescence of ginger, which was consistent with the growth trend of ginger in the field. In addition, the *R. palustris* PSB-06 could change the structures and diversities of bacterial and fungal communities to increase probiotic bacteria while decrease those of soil-borne pathogenic fungi, it also could increase soil nutrient contents. All these factors were integrated into together to promote ginger growth, and led to a higher ginger yield. There results were further proved that *R. palustris* PSB-06 had great potential in the field application after soil dazomet fumigation especially ginger.

## Conclusion

In our study, the combination of *R. palustris* PSB-06 and dazomet, which was an eco-friendly and efficient control method against root-knot nematodes on ginger, was recommended in field. Firstly, the combination of *R. palustris* PSB-06 and dazomet treatments could effectively control root-knot nematodes. Secondly, *R. palustris* PSB-06 could effectively improve soil physicochemical properties, and change the structures and diversities of bacterial and fungal communities. It could enhance the ginger resistance to pathogens (including root-knot nematodes), or might had antagonistic effect on root-knot nematode. Therefore, it was necessary to apply *R. palustris* PSB-06 after soil dazomet fumigation in field.

## Data availability statement

The datasets presented in this study can be found in online repositories. The names of the repository/repositories and accession number(s) can be found in the article/Supplementary material.

## Author contributions

FC, YL, and DW: designed the experiments. DW, JW, PS, and JD: performed the experiments. DW, XT, and DZ: analyzed the data. DW, FC, and YL: wrote the manuscript. All authors read and approved the final version of the manuscript.

## Funding

This work was supported by National Natural Science Foundation of China (no. 31871941), Natural Science Foundation of Hunan Province (no. 2022JJ40235), Changsha Municipal Natural Science Foundation (no. Kq2014177), and Shaanxi Key Laboratory of Plant Nematology (no. 2021-SKL-02).

## Conflict of interest

The authors declare that the research was conducted in the absence of any commercial or financial relationships that could be construed as a potential conflict of interest.

## Publisher’s note

All claims expressed in this article are solely those of the authors and do not necessarily represent those of their affiliated organizations, or those of the publisher, the editors and the reviewers. Any product that may be evaluated in this article, or claim that may be made by its manufacturer, is not guaranteed or endorsed by the publisher.

## Footnotes

^1^ www.biocloud.net

^2^ https://www.ncbi.nlm.nih.gov/sra
