# Supplementary material for: Effects of dazomet combined with Rhodopsesudomonas palustris PSB-06 on root-knot nematode, Meloidogyne incognita infecting ginger and soil microorganisms diversity
Source: Front Microbiol. 2022 Sep 29;13:1021445. doi: 10.3389/fmicb.2022.1021445 (PMC9558219; doi:10.3389/fmicb.2022.1021445)
Supplement: Supplementary file 1 [file Data_Sheet_1.doc]

**Supporting Information**

**Contents**

Result S1: Treatment using *R. palustris* PSB-06 and dazomet influenced chlorophyll in ginger leaf.

Result S2: Sequences assembly.

Result S3: Microbial diversity and community composition in ginger soil.

Result S4: Alpha diversity and structure of microbial-community.

Result S5: Application of *R. palustris* PSB-06 influences physicochemical properties of ginger soil.

Result S6: Effects of different treatments on beta diversity and the relationship between soil microorganism and physicochemical property at the harvest stage.

**Figure Legends**

Figure S1. Leaf physiological characteristics of ginger at different developmental stages.

Figure S2. The characteristic venn graph in bacterial and fungal samples.

Figure S3. The phylogenetic tree of bacteria (A) and fungi (B) at genus level

Figure S4. Shannon and Simpson indices of soil bacterial and fungal diversities at the four different growth stages.

Figure S5. Shannon and Simpson indices of the soil bacterial and fungal diversity at different growth stages.

Figure S6. Relative of bacteria (*Paenibacillus* spp., *Stenotrophomonas* spp., *Pseudomonas* spp. and *Ralstonia* spp.) and soil-borne pathogenic fungi (*Fusarium spp.*) at different growth stages.

Figure S7. The ginger soil properties.

Figure S8. Principal coordinates analysis (PCoA) and non-MetricMulti-Dimensional Scaling (NMDS) analysis based on Bray–Curtis dissimilarity matrices at harvest stage.

**Table Legends**

Table S1. Statistics of bacterial sample sequencing data.

Table S2. Statistics of fungal sample sequencing data.

Table S3. The OTUs in bacterial and fungal samples.

Table S4. The statistics of alpha diversity indices of soil bacteria.

Table S5. The statistics of alpha diversity indices of soil fungi.

Table S6. The statistics of sequences in each grade of soil bacteria.

Table S7. The statistics of sequences in each grade of soil fungi.

Table S8. The statistics of species in each grade of soil bacteria.

Table S9. The statistics of species in each grade of soil fungi.

Table S10. Taxonomic structure of the soil bacterial diversity at the phylum level.

Table S11. Taxonomic structure of the soil fungal diversity at the phylum level.

Table S12. Taxonomic structure of the soil bacterial diversity at the family level.

Table S13. Taxonomic structure of the soil fungal diversity at the family level.

Table S14. Significant difference analysis (p<0.05) of the top 16 common bacterial families from at different developmental stages.

Table S15. Significant difference analysis (p<0.05) of the top 11 common fungal families from different developmental stages.

Table S16. Taxonomic structure of the soil bacterial diversity at the genera level.

Table S17. Taxonomic structure of the soil fungal diversity at the genera level.

Table S18. Detrended correspondence analysis (DCA) indices of soil microorganisms.

Table S19. Mantel test of soil bacteria at the phylum level with soil physicochemical properties.

Table S20. Mantel test of soil fungi at the phylum level with soil physicochemical properties.

Table S21. Mantel test of soil bacteria at the family level with soil physicochemical properties.

Table S22. Mantel test of soil fungi at the family level with soil physicochemical properties.

**Supporting Results**

**1. Treatment using *R. palustris* PSB-06 and dazomet influenced chlorophyll in ginger leaf**

Analysis of chlorophyll a in the leaf samples showed that the changes of chlorophyll a in the leaf samples were similar to that observed for the total chlorophyll contents, except that no significant statistical differences (p>0.05) were observed between the samples collected from the T1 and T3 treatments at the harvest stage (Figure S1A in File S1). Analysis of chlorophyll b content showed that the changes of chlorophyll b contents in these leaf samples were also similar to that described for the total chlorophyll above, except the chlorophyll b content in the leaf samples collected from the T3 treatment was similar to that observed in the leaf samples collected from the T2 and T4 treatments at the harvest stage (Figure S1B in File S1). Again, no significant statistical differences (p>0.05) were found for chlorophyll b contents in the leaf samples collected from the T2 and T4 treatments at the three growth stages.

**2. Sequences assembly**

A total of 6,677,506 high quality reads representing the V3-V4 region in the bacterial 16S rRNA gene and 6,037,085 high quality reads representing the fungal ITS1 region were obtained from the soil samples (Table S1-S2 in File S1). The maximum value of effective bacterial sequences (97,463.60) was found in the sample collected from the T1 treatment at the germination stage, and the maximum value of effectiveness (Effective sequences/raw sequences×100) was 94.20%, found in the sample collected from the T4 treatment at the harvest stage. The maximum value of effective fungal sequences (78,794.20) and effectiveness (98.45%) were found in the samples collected from the T1 treatment at the vigorous growth stage. A total of 1,833 and 1,069 operational taxonomic units (OTUs, 97% identity) were found to be bacterial and fungal sequences, respectively (Table S3 in File S1). The highest bacterial and fungal OTU numbers (1,599 and 721) were found in the samples collected from the T2 treatment at the harvest stage and the samples collected from the T4 treatment at the vigorous growth stage, respectively. The OTUs found in all the samples collected from four sampling stages represented the majority OTUs, and the number of fungal OTUs commonly found in the samples collected from different treatments was higher than the number of bacterial OTUs (Figure S2 in File S1). The statistics of soil bacteria and fungi alpha diversity indices showed that the Ace and Chao1 indices were closely related and the coverage were all above 99% (Table S4-S5 in File S1), indicating the breadth and depth of the sequencing results.

**3.** **Microbial diversity and community composition in ginger soil**

After removal of low abundance sequences (species abundance less than 0.005%), the effective sequences were annotated to Kindom, Phylum, Class, Order, Family, Genus, and Species categories (Table S6-S7 in File S1). The numbers of species in each grade in individual samples are shown in Table S8-S9 in File S1. The species annotation and taxonomic analyses showed that the 19 to 24 Phyla were found in the bacterial samples, while 6 to 8 Phyla were found in the fungal samples collected from different treatments and at different growth stages. The dominant bacterial Phyla found in the four treatment samples collected at the germination stage were Proteobacteria, Bacteroidetes, and Actinobacteria. The dominant bacterial Phyla found in the four treatment samples collected at the other three growth stages were Proteobacteria, Acidobacteria, and Gemmatimonadetes (Table S10-S11 in File S1). For fungi, the dominant Phyla found in the four treatment samples collected at the germination, seedling, and harvest stages were Ascomycota and Basidiomycota, except that in the T2 treatment sample collected at the seedling stage (Ascomycota and Unclassified). The dominant Phyla found in the four treatment samples collected at the vigorous growth stage were Ascomycota and Unclassified (Table S10-S11 in File S1). The phylogenetic bacterial and fungal trees showed that at the genus level, the majority genera could be clustered together (Figure S3 in File S1).

**4.** **Alpha diversity and structure of microbial-community**

The bacterial Shannon indices of the T1, T2, and T3 treatment samples collected at the germination stage was significantly lower (p<0.01) than that of the T1, T2, and T3 treatment samples collected at other three growth stages (Figure S4 in File S1). No significant statistical difference (p>0.05) was observed among the samples collected at the seedling, vigorous growth, and harvest stages. The Simpson indices of the T2 and T3 treatment samples were similar to the Shannon indices, but the Simpson index of the T1 treatment sample collected at the germination stage was significant lower (p<0.01) than that of the samples collected at the seedling and vigorous growth stages. The Simpson indices of the samples collected at the seedling stage was significantly lower (p<0.05) than that of the samples collected at the vigorous growth and harvest stages. No significant statistical differences (p>0.05) were found among the four treatments in T4 treatment (Figure S4 in File S1). The Shannon indices of the T2 and T3 treatment samples, or the T2 and T4 treatment samples were significantly different (p<0.05) at the germination stage as well as the T1 and T3 treatment samples at the seedling stage (Figure S5 in File S1). No significant statistical differences (p>0.05) were found among the other treatments.

For fungal population structure, The Shannon indices of all the treatment samples collected at the harvest stage were the highest, while at the seedling stage, the Shannon indices of these samples, except the T3 treatment sample, were greater than that of the samples collected at the germination and vigorous growth stages (Figure S4 in File S1). The Simpson indices of the samples collected at the harvest stage were the highest, and were also significantly higher (p<0.05) than that of the T1 and T2 treatment samples collected at the germination and vigorous growth stages, T3 treatment samples collected at the germination and seedling stages as well as the T4 treatment sample collected at the germination stage. The Shannon and Simpson indices of the T3 treatment samples were significantly different (p<0.05) from that of the T4 treatment samples collected at the germination stage (Figure S5 in File S1).

**5. Application of *R. palustris* PSB-06 influences physicochemical properties of ginger soil**

Effects of the four treatments on nutrient utilization by ginger were measured using the samples collected at the germination and harvest stages (Figure S7 in File S1). The result showed that the pH values of the soil samples collected from the T1 and T2 treatments at the harvested stage were unchanged compared to that of the samples collected at the germination stage (Figure S7A in File S1). Analyses of total nitrogen, hydrolytic nitrogen, phosphorus, and potassium contents in these soil samples showed that at the germination stage (Figure S7C-H in File S1), the T1 and T2 treatments decreased the content of nitrogen compared to the T3 and T4 treatments at the germination stage, these differences became small at the harvest stage (Figure S7C in File S1). Similar results were observed for the hydrolytic nitrogen contents at the germination stage. However, at the harvest stage, the hydrolytic nitrogen contents in the soil samples collected from the T1 and T3 treatments were clearly increased (Figure S7D in File S1). The total phosphorus contents in the T1 and T2 treatment soil samples were lower than that in the T3 and T4 treatment soil samples at the germination stage. At the harvest stage, however, these differences became small (Figure S7E in File S1). The total potassium contents in the T1 and T2 treatment soil samples were higher than that in the T3 and T4 treatment soil samples at the germination stage. At the harvest stage, however, these differences also became small (Figure S7G in File S1). The result showed that the available potassium of the soil samples collected from the four treatments was similar at the germination. However, compared to the samples collected from the T2 and T4 treatments at the harvest stage, the available potassium of the T1 and T3 samples were much higher (Figure S7H in File S1).

**6.** **Effects of different treatments on beta diversity and the relationship between soil microorganism and physicochemical property at the harvest stage**

Differences of microorganisms in various soil samples collected at the harvest stage were analyzed through the principal coordinates analysis (PCoA) and the non-MetricMulti-Dimensional Scaling (NMDS) method. The PCoA result indicated that the bacterial and fungal populations in the T4 treatment sample was clearly different from that of the other three treatment samples (Figure S8A-B in File S1). The bacterial population points were widely scattered. The first coordinate (PC1) showed 43.40% difference, while the second coordinate (PC2) showed 8.13% difference (Figure S8A in File S1). The fungal population points were almost centralized, except for the soil sample treated with *R. palustris* PSB-06 and dazomet. The PC1 showed 54.75% difference, while the PC2 showed 13.21% difference (Figure S8B in File S1). The NMDS result showed that the bacterial and fungal populations in the T4 treatment sample were also clearly different from that of other three treatment samples with 0.0235 stress (bacterial) and 0.0977 stress (fungal) (Figure S8C-D in File S1).

Significant positive correlations were found between the bacterial communities and the soil physicochemical properties (p<0.05, Table S19 in Supplementary File S1). The correlation between the available phosphorus and Dependentiae (r=0.739, p<0.01), Patescibacteria (r=0.724, p<0.01) or Rokubacteria (r=0.661, p<0.01), and the correlation between the pH value and Rokubacteria (r=0.707, p<0.01), Firmicutes (r=0.645, p<0.01), Patescibacteria (r=0.622, p<0.01) or Dependentiae (r=0.543, p<0.01) were all significantly positive, indicating that Dependentiae, Patescibacteria and Rokubacteria are closely correlated with the levels of soil pH value and available phosphorus. The RDA1 and RDA2 values accounted for 24.70% and 15.02% of the total fungal variations, respectively (Figure 4B). The correlations between the fungal communities and soil physicochemical properties were also positive (p<0.05, Table S20 in Supplementary File S1). Significant positive correlations (p<0.01) were observed between the available phosphorus and Basidiomycota (r=0.691, p<0.01), Mucoromycota (r=0.613, p<0.01) or Ascomycota (r=0.565, p<0.01), and the pH value and Mucoromycota (r=0.637, p<0.01) (Table S20 in Supplementary File S1), indicating that Mucoromycota is also closely correlated to the levels of pH value and available phosphorus.

**Figures**


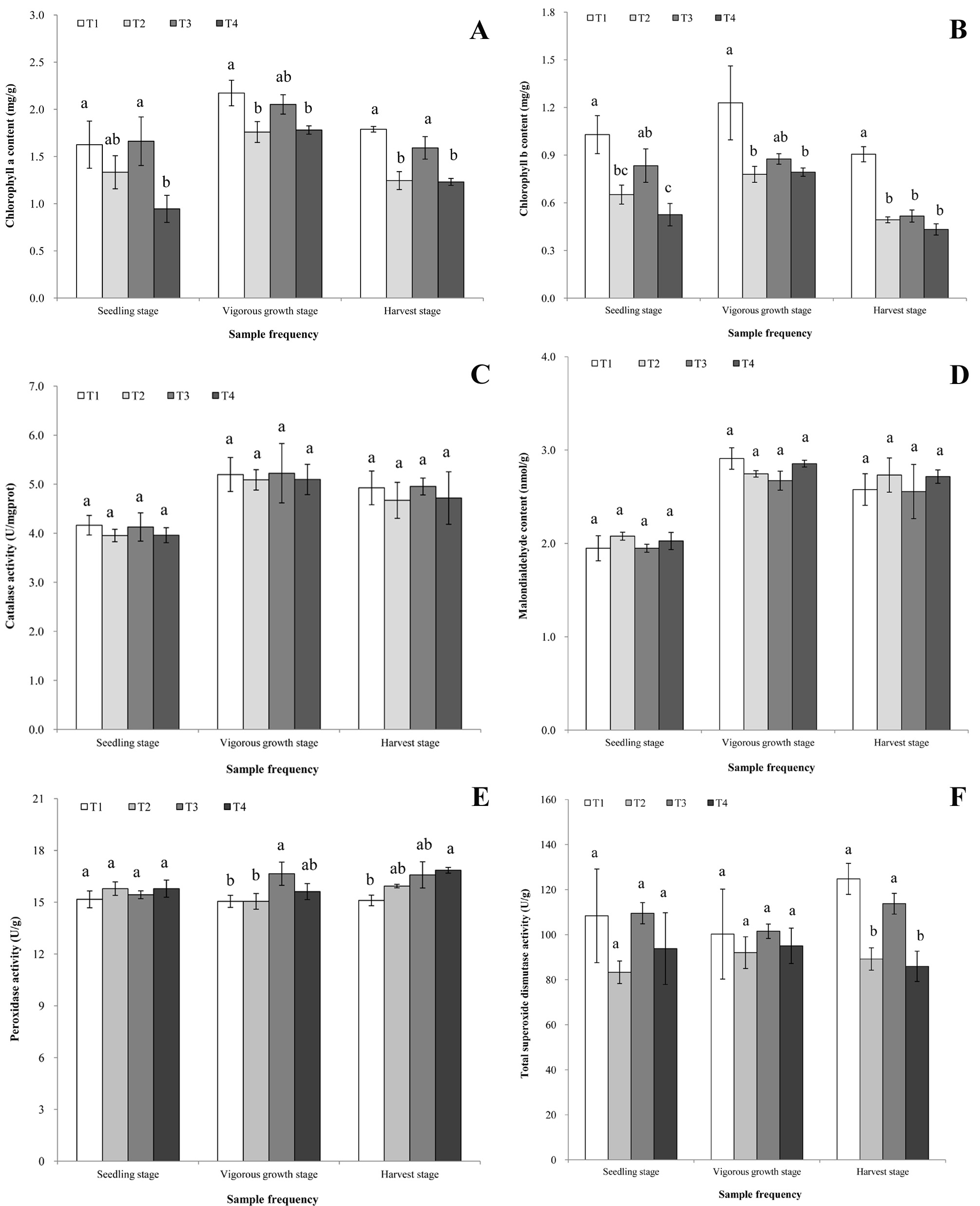


**Figure S1. Leaf physiological characteristics of ginger at different developmental stages.**

A: The chlorophyll a content, B: The chlorophyll b content, C: The catalase (CAT) content, D: The malondialdehyde (MDA) content, E: The peroxidase (POD) content, F: Total superoxide dismutase (T-SOD) content. T1, soil treated with *R. palustris* PSB-06 and dazomet; T2, soil treated with dazomet alone; T3, soil treated with *R. palustris* PSB-06 alone; T4, soil without treatment (blank control). Same low case letters indicate no significant statistical difference (p>0.05) among the treatments, determined using the Duncan’s multiple range test.


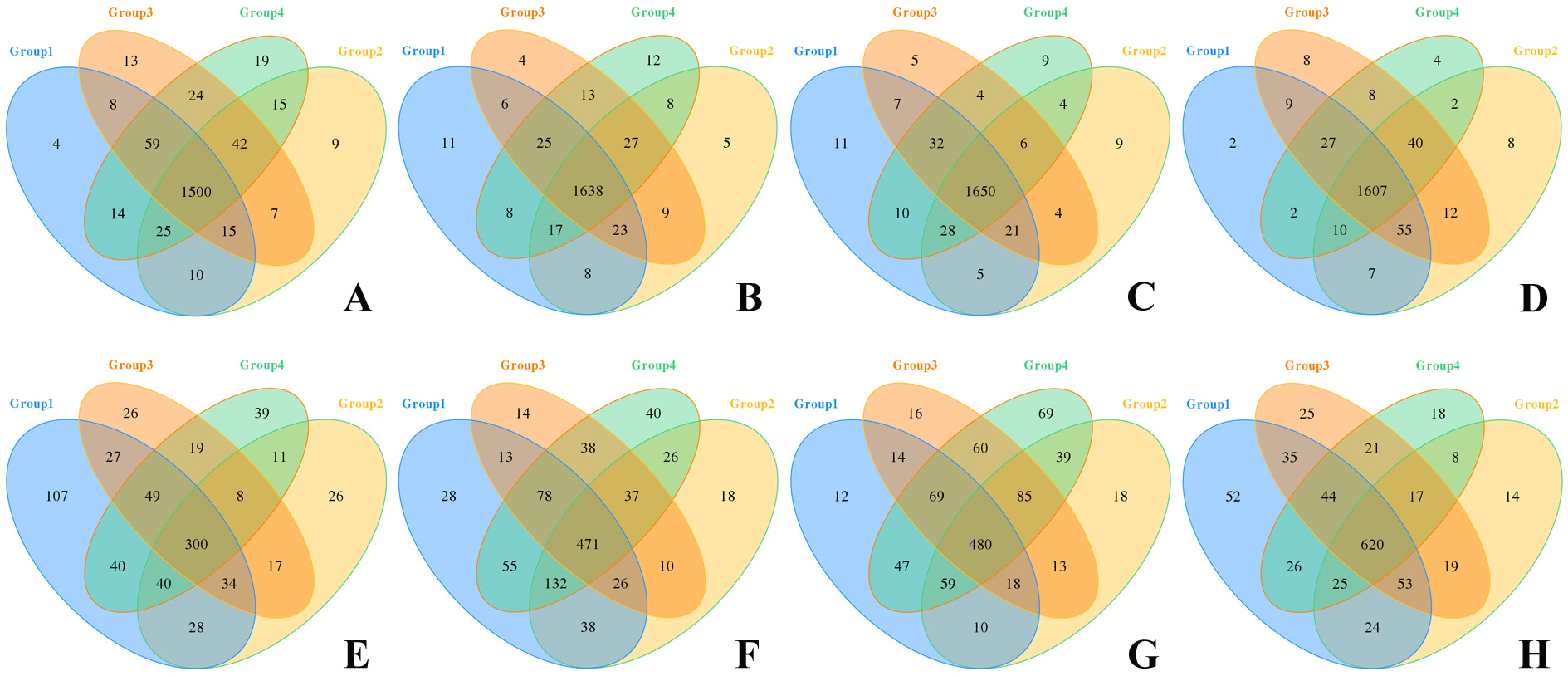


**Figure S2. The characteristic venn graph in bacterial and fungal samples.**

A-D: the sample frequency (Germination stage, Seedling stage, Vigorous growth stage, Harvest stage) in bacterial operational taxonomic units (OTUs), E-H: the sample frequency (Germination stage, Seedling stage, Vigorous growth stage, Harvest stage) in fungal OTUs. Group 1 in A-D: soil treated with *R. palustris* PSB-06 and dazomet, Group 2 in A-D: soil treated with dazomet alone, Group 3 in A-D: soil treated with *R. palustris* PSB-06 alone, Group 4 in A-D: soil without treatment (blank control). Group 1-Group 4 in E: soil treated with *R. palustris* PSB-06 and dazomet, Group 1- Group 4 in F: soil treated with dazomet alone, Group 1- Group 4 in G: soil treated with *R. palustris* PSB-06 alone, Group 1- Group 4 in H: soil without treatment (blank control).


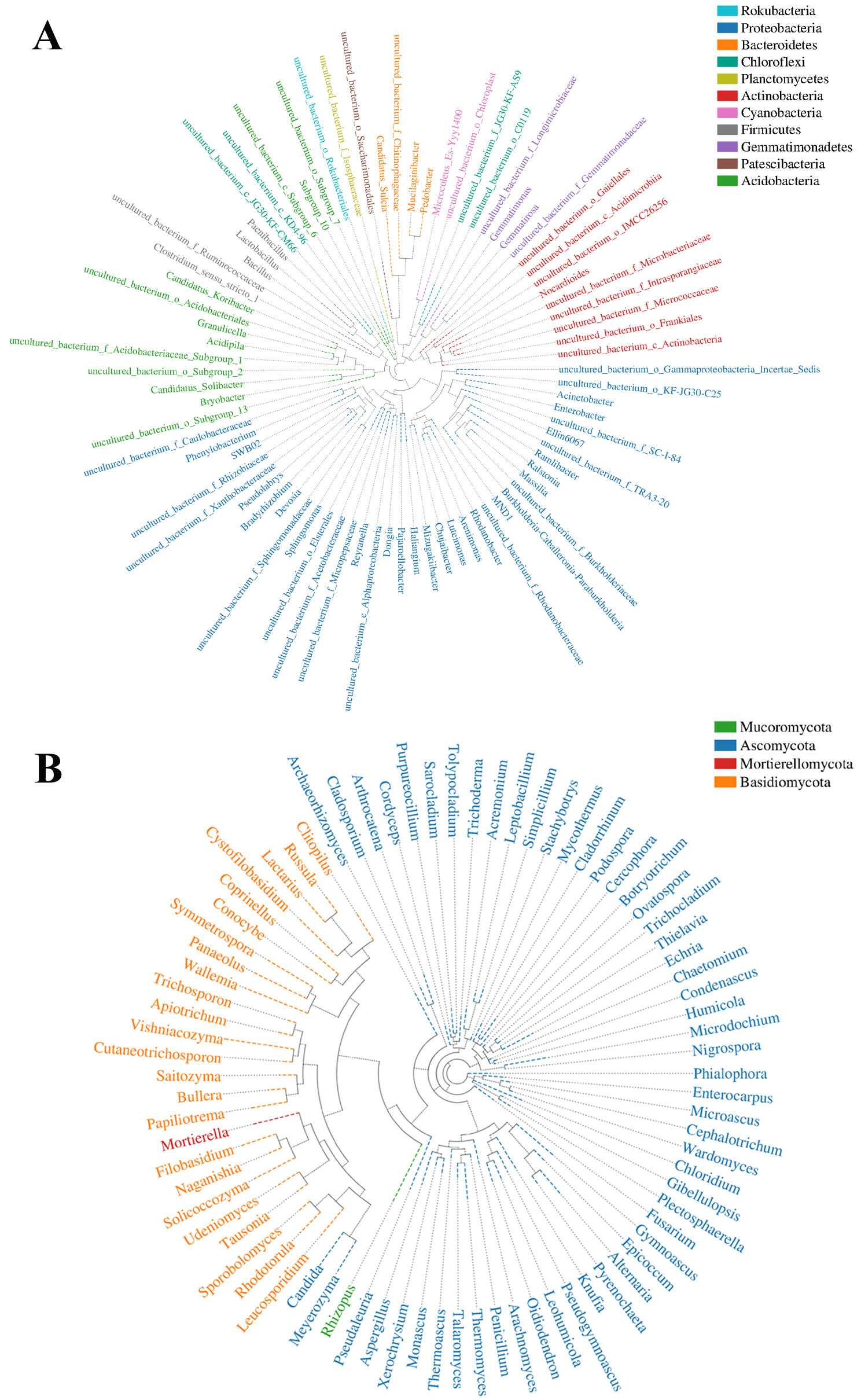


**Figure S3. The phylogenetic tree of bacteria (A) and fungi (B) at genus level**


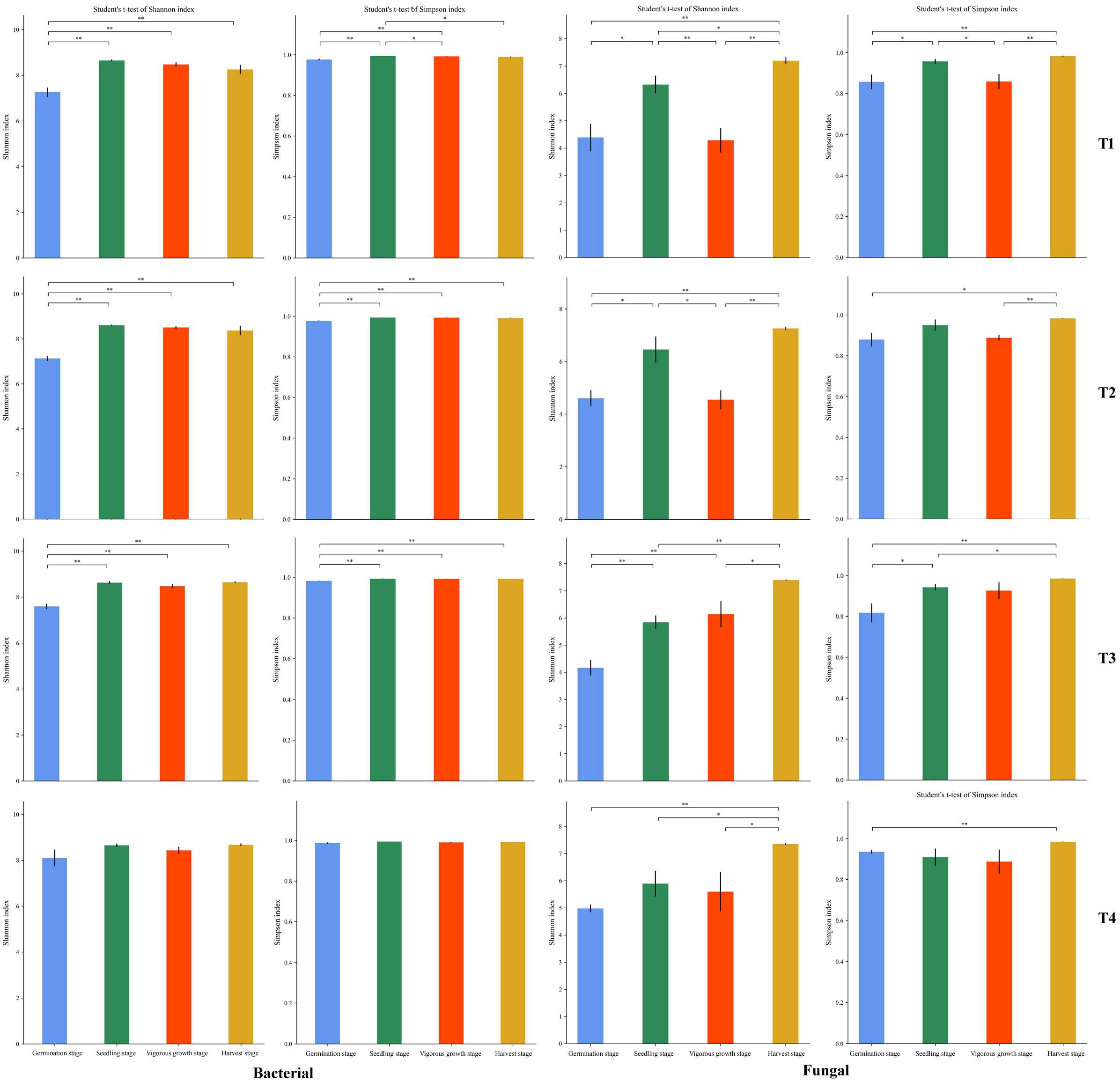


**Figure S4. Shannon and Simpson indices of soil bacterial and fungal diversities at the four different growth stages.**

T1, soil treated with *R. palustris* PSB-06 and dazomet; T2, soil treated with dazomet alone; T3, soil treated with *R. palustris* PSB-06 alone; T4, soil without treatment (blank control). Asterisks indicate significant statistical differences between two treatment (p<0.05 and p<0.01), determined through the Student’s t-test.


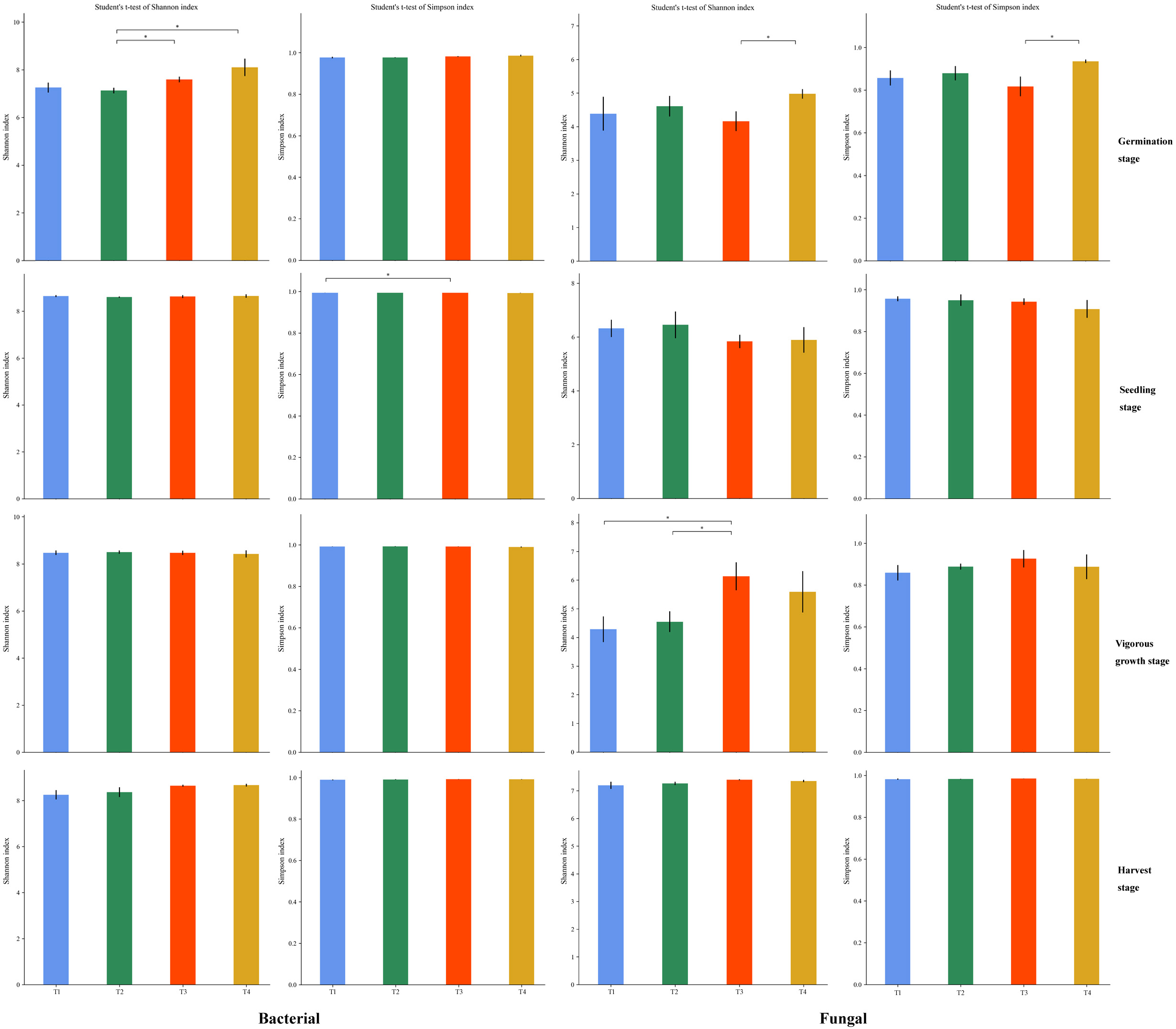


**Figure S5. Shannon and Simpson indices of the soil bacterial and fungal diversity at different treatments.**

T1, soil treated with *R. palustris* PSB-06 and dazomet; T2, soil treated with dazomet alone; T3, soil treated with *R. palustris* PSB-06 alone; T4, soil without treatment (blank control). Asterisks indicate means are significantly different (p<0.05 and p<0.01) between two treatments according to Student’s t-test.


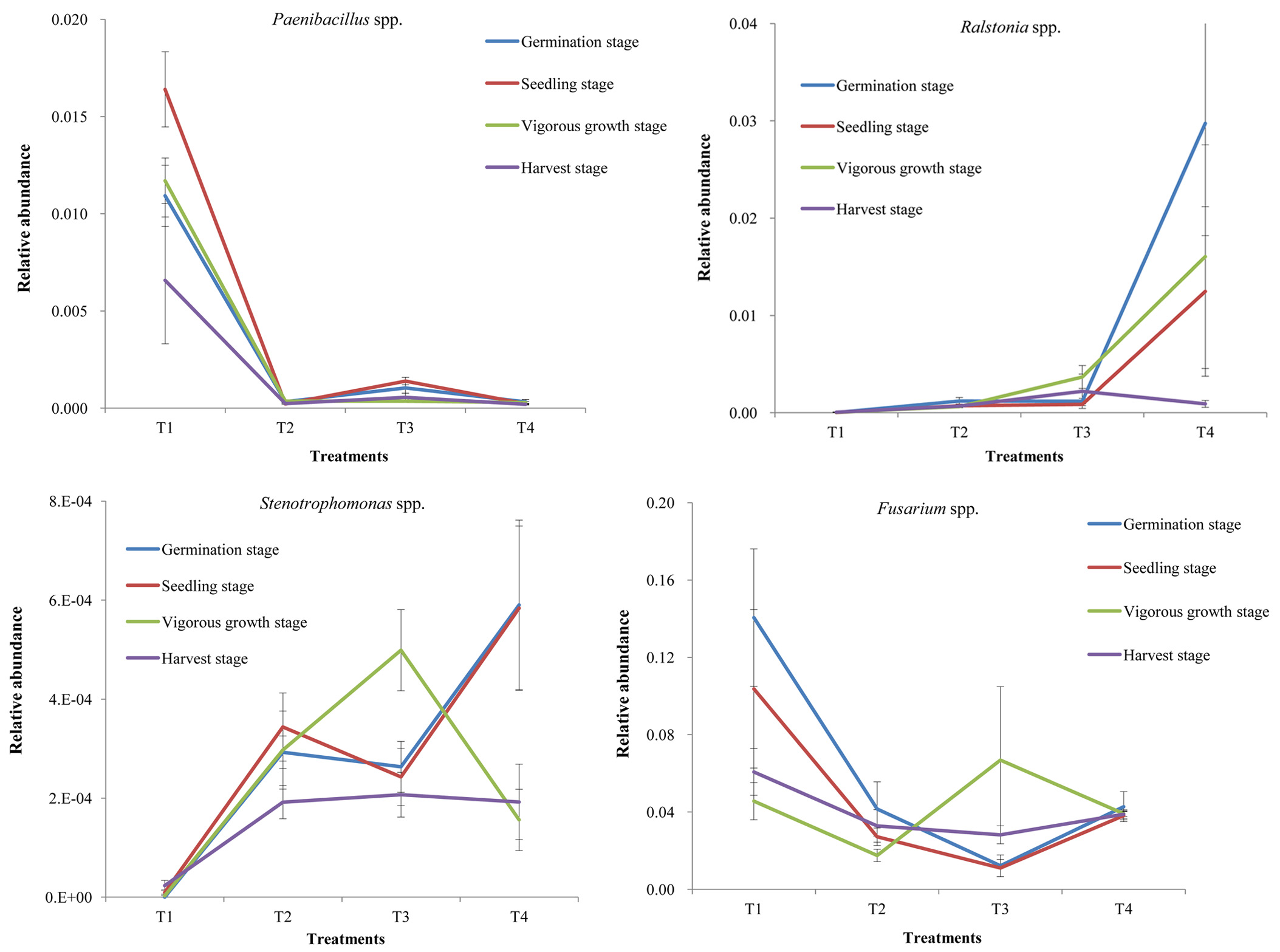


**Figure S6. Relative of bacteria (*Paenibacillus* spp., *Ralstonia* spp., and *Stenotrophomonas* spp.) and soil-borne pathogenic fungi (*Fusarium* spp.) at different growth stages.**

T1, soil treated with *R. palustris* PSB-06 and dazomet; T2, soil treated with dazomet alone; T3, soil treated with *R. palustris* PSB-06 alone; T4, soil without treatment (blank control). The statistical differences between the treatments were determined using the Duncan’s Multiple Range Test (DMRT) at the 5% level.

**
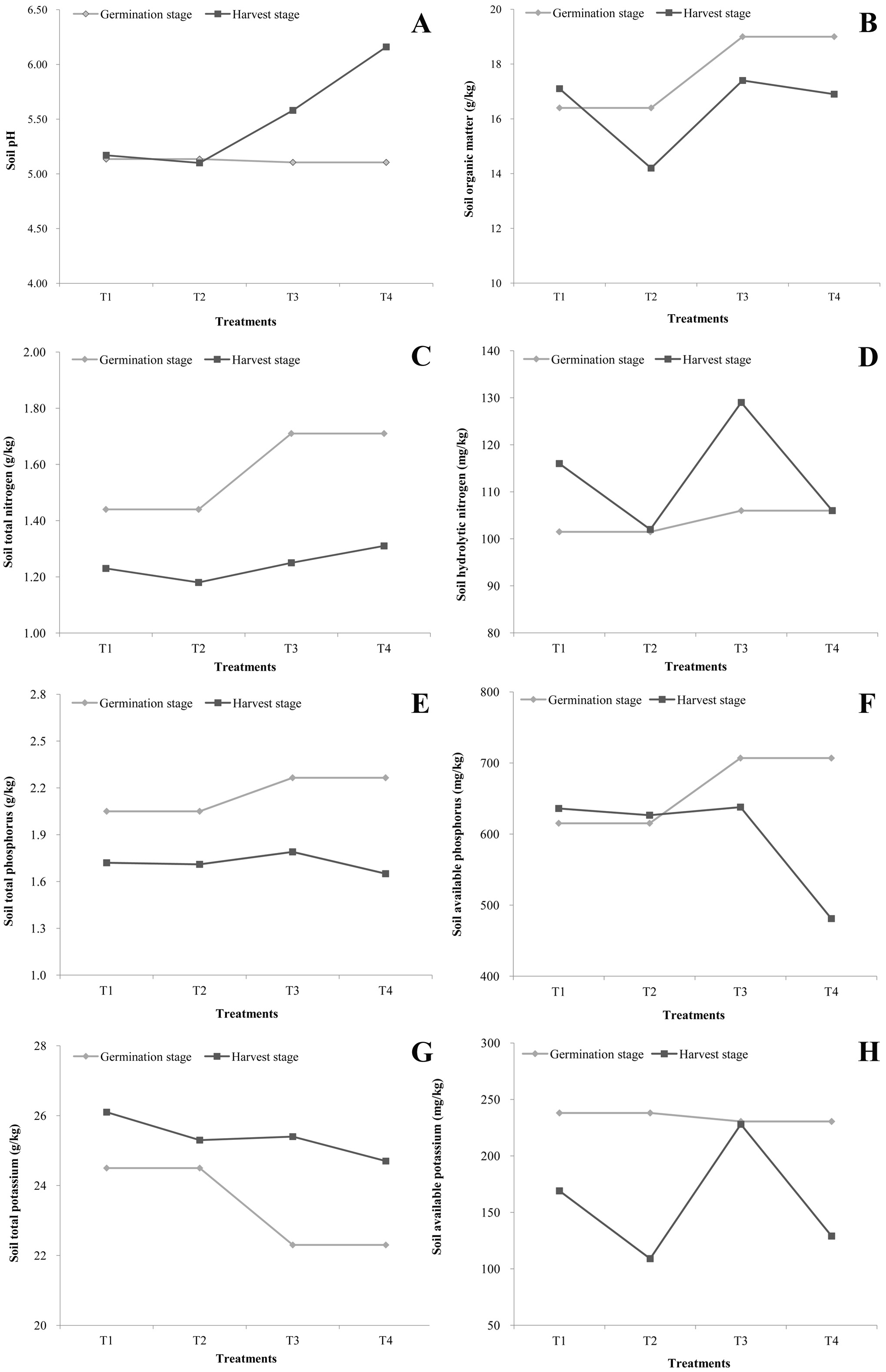
**

**Figure S7. The ginger soil properties.**

A: Soil pH, B: Soil organic matter, C: Soil total nitrogen, D: Soil hydrolytic nitrogen, E: Soil total phosphorus, F: Soil available phosphorus, G: Soil total potassium, H: Soil available potassium. T1, soil treated with *R. palustris* PSB-06 and dazomet; T2, soil treated with dazomet alone; T3, soil treated with *R. palustris* PSB-06 alone; T4, soil without treatment (blank control).


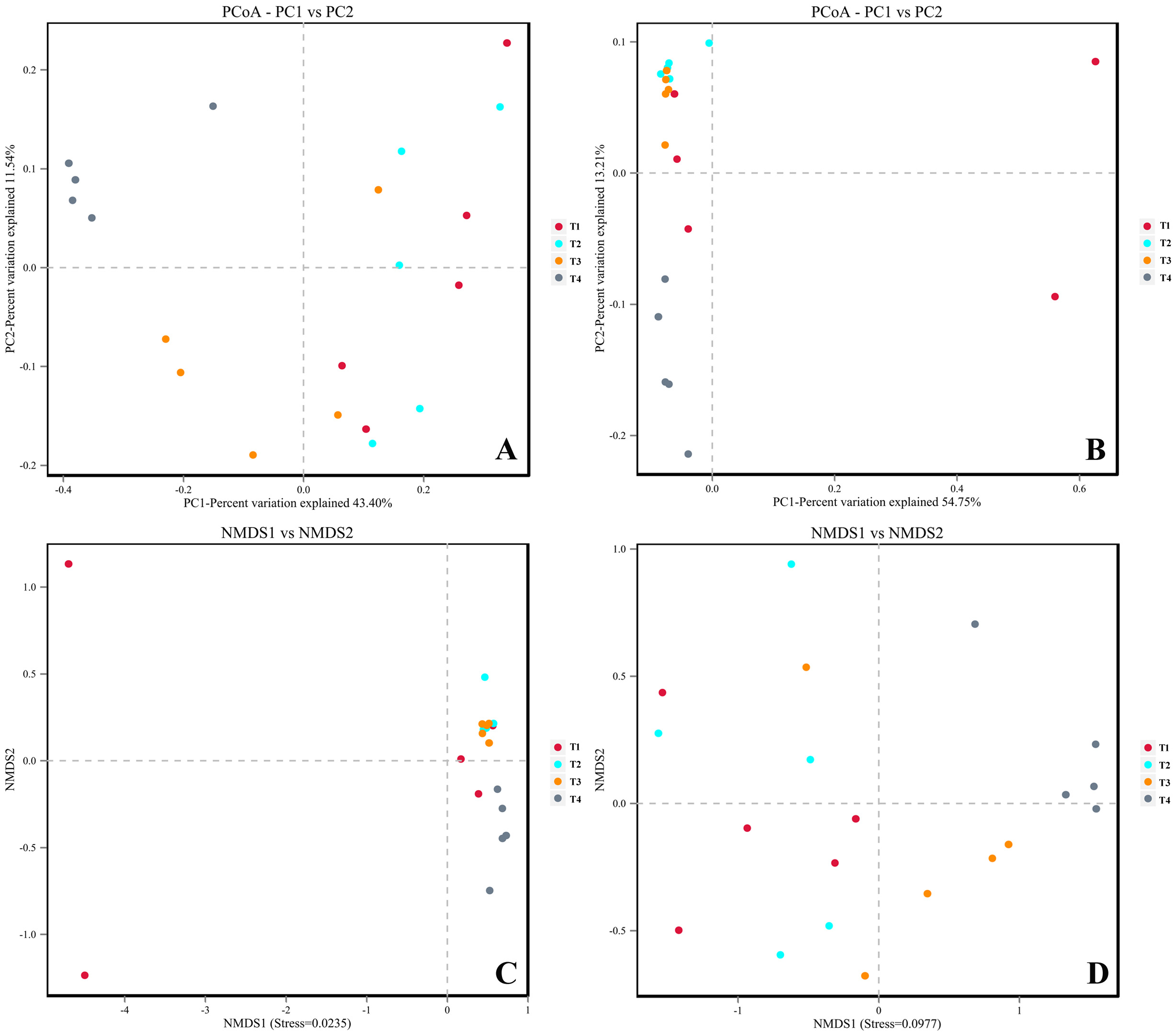


**Figure S8. Principal coordinates analysis (PCoA) and non-MetricMulti-Dimensional Scaling (NMDS) analysis based on Bray–Curtis dissimilarity matrices at harvest stage.**

A, C: PCoA analysis of soil bacteria and fungi, B, D: NMDS analysis of soil bacteria and fungi, the distance between points indicates the degree of difference; the closer the distance, the higher the similarity. T T1, soil treated with *R. palustris* PSB-06 and dazomet; T2, soil treated with dazomet alone; T3, soil treated with *R. palustris* PSB-06 alone; T4, soil without treatment (blank control). OM: Organic matter, TN: Total nitrogen, AN: Hydrolytic nitrogen, TP: Total phosphorus, AP: Available phosphorus, TK: Total potassium, AK: Available potassium.

**Tables**

**Table S1. Statistics of bacterial sample sequencing data.**

| Sample ID | Raw Sequences | Clean Sequences | Effective Sequences | AvgLen(bp) | GC(%) | Q20(%) | Q30(%) | Effective(%) |
| --- | --- | --- | --- | --- | --- | --- | --- | --- |
| A1 | 161676 | 116720 | 99137 | 420 | 55.01 | 98.31 | 94.06 | 61.32 |
| A2 | 144381 | 105678 | 89867 | 420 | 54.69 | 98.28 | 93.99 | 62.24 |
| A3 | 129320 | 97059 | 83943 | 417 | 55.94 | 98.37 | 94.21 | 64.91 |
| A4 | 169481 | 120353 | 103168 | 418 | 55.79 | 98.34 | 94.12 | 60.87 |
| A5 | 179477 | 128827 | 111203 | 418 | 55.85 | 98.36 | 94.18 | 61.96 |
| B1 | 162650 | 123236 | 106731 | 420 | 54.26 | 98.35 | 94.16 | 65.62 |
| B2 | 72431 | 50522 | 44932 | 418 | 55.1 | 98.3 | 94 | 62.03 |
| B3 | 134644 | 97450 | 84631 | 419 | 54.98 | 98.35 | 94.14 | 62.86 |
| B4 | 120119 | 87699 | 75639 | 418 | 55.69 | 98.36 | 94.2 | 62.97 |
| B5 | 114820 | 83206 | 73713 | 418 | 54.82 | 98.4 | 94.28 | 64.2 |
| C1 | 106300 | 78571 | 67835 | 419 | 54.96 | 98.42 | 94.34 | 63.81 |
| C2 | 150688 | 110520 | 95106 | 418 | 55.67 | 98.39 | 94.26 | 63.11 |
| C3 | 105889 | 79076 | 68211 | 418 | 55.98 | 98.45 | 94.42 | 64.42 |
| C4 | 150393 | 114624 | 99781 | 418 | 55.98 | 98.41 | 94.33 | 66.35 |
| C5 | 163615 | 118403 | 103298 | 417 | 55.85 | 98.4 | 94.26 | 63.13 |
| D1 | 157573 | 114636 | 98253 | 420 | 54.54 | 98.35 | 94.14 | 62.35 |
| D2 | 129280 | 94074 | 83880 | 417 | 56.57 | 98.39 | 94.27 | 64.88 |
| D3 | 144760 | 105009 | 91012 | 418 | 56.1 | 98.41 | 94.31 | 62.87 |
| D4 | 130311 | 95802 | 83334 | 417 | 56.44 | 98.44 | 94.42 | 63.95 |
| D5 | 118394 | 88211 | 79940 | 417 | 56.7 | 98.44 | 94.4 | 67.52 |
| E1 | 79973 | 77957 | 75390 | 417 | 57.26 | 98.26 | 94.05 | 94.27 |
| E2 | 79821 | 77838 | 75135 | 417 | 56.98 | 98.29 | 94.09 | 94.13 |
| E3 | 79538 | 77466 | 74794 | 417 | 57 | 98.3 | 94.14 | 94.04 |
| E4 | 79907 | 78060 | 74945 | 417 | 56.83 | 98.32 | 94.18 | 93.79 |
| E5 | 79779 | 77610 | 74973 | 417 | 57.14 | 98.27 | 94.09 | 93.98 |
| F1 | 80083 | 78180 | 74777 | 417 | 57.23 | 98.38 | 94.34 | 93.37 |
| F2 | 80148 | 78288 | 75919 | 417 | 57.51 | 98.43 | 94.45 | 94.72 |
| F3 | 80313 | 78335 | 74987 | 417 | 57.26 | 98.38 | 94.35 | 93.37 |
| F4 | 79947 | 77743 | 75090 | 418 | 57.44 | 98.34 | 94.24 | 93.92 |
| F5 | 80200 | 78097 | 75752 | 416 | 57.17 | 98.38 | 94.33 | 94.45 |
| G1 | 80483 | 78166 | 74995 | 417 | 57.66 | 98.39 | 94.37 | 93.18 |
| G2 | 80077 | 77984 | 73887 | 417 | 57.24 | 98.31 | 94.14 | 92.27 |
| G3 | 80253 | 78184 | 75950 | 417 | 57.86 | 98.34 | 94.2 | 94.64 |
| G4 | 79941 | 77816 | 75538 | 418 | 57.12 | 98.33 | 94.22 | 94.49 |
| G5 | 80080 | 77981 | 75466 | 417 | 57.58 | 98.38 | 94.3 | 94.24 |
| H1 | 80159 | 77678 | 75214 | 419 | 57.55 | 98.27 | 94.09 | 93.83 |
| H2 | 80036 | 77718 | 75726 | 420 | 57.39 | 98.24 | 93.99 | 94.61 |
| H3 | 80325 | 77492 | 75023 | 419 | 57.81 | 98.28 | 94.07 | 93.4 |
| H4 | 79978 | 78125 | 76312 | 416 | 57.52 | 98.38 | 94.29 | 95.42 |
| H5 | 80310 | 78041 | 75279 | 416 | 57.57 | 98.32 | 94.16 | 93.74 |
| I1 | 80336 | 78333 | 74478 | 419 | 56.64 | 98.25 | 93.98 | 92.71 |
| I2 | 79711 | 77561 | 73682 | 418 | 56.86 | 98.29 | 94.08 | 92.44 |
| I3 | 80260 | 78554 | 74609 | 417 | 56.3 | 98.33 | 94.19 | 92.96 |
| I4 | 79938 | 77899 | 75085 | 417 | 57.4 | 98.29 | 94.11 | 93.93 |
| I5 | 79763 | 77903 | 72927 | 417 | 56.25 | 98.25 | 93.99 | 91.43 |
| J1 | 79873 | 78184 | 75118 | 416 | 56.39 | 98.41 | 94.37 | 94.05 |
| J2 | 80123 | 78110 | 75469 | 417 | 57.19 | 98.31 | 94.17 | 94.19 |
| J3 | 79883 | 77873 | 75465 | 417 | 57.17 | 98.3 | 94.11 | 94.47 |
| J4 | 79602 | 77321 | 74700 | 416 | 57.32 | 98.34 | 94.22 | 93.84 |
| J5 | 79937 | 77949 | 74754 | 416 | 57.24 | 98.37 | 94.29 | 93.52 |
| K1 | 80107 | 77781 | 74997 | 417 | 57.22 | 98.29 | 94.12 | 93.62 |
| K2 | 80037 | 77955 | 74874 | 416 | 57.62 | 98.32 | 94.19 | 93.55 |
| K3 | 79955 | 77973 | 75253 | 416 | 57.56 | 98.33 | 94.2 | 94.12 |
| K4 | 79673 | 77933 | 69574 | 416 | 57.7 | 98.38 | 94.32 | 87.32 |
| K5 | 79987 | 78160 | 74275 | 417 | 57.39 | 98.29 | 94.12 | 92.86 |
| L1 | 79885 | 77039 | 73954 | 419 | 58.14 | 98.22 | 93.96 | 92.58 |
| L2 | 79748 | 77458 | 73135 | 418 | 57.14 | 98.27 | 94.07 | 91.71 |
| L3 | 80079 | 78010 | 74396 | 417 | 57.15 | 98.33 | 94.24 | 92.9 |
| L4 | 80002 | 77713 | 73123 | 419 | 57.18 | 98.25 | 94 | 91.4 |
| L5 | 80040 | 77944 | 74221 | 417 | 57.11 | 98.29 | 94.07 | 92.73 |
| M1 | 79808 | 78025 | 74598 | 417 | 56.63 | 98.3 | 94.11 | 93.47 |
| M2 | 80378 | 78322 | 75276 | 417 | 56.67 | 98.36 | 94.25 | 93.65 |
| M3 | 79982 | 78277 | 74570 | 418 | 55.95 | 98.24 | 94 | 93.23 |
| M4 | 80235 | 78273 | 74827 | 417 | 56.34 | 98.27 | 94.02 | 93.26 |
| M5 | 79914 | 77942 | 73498 | 418 | 56.4 | 98.23 | 93.94 | 91.97 |
| N1 | 71622 | 70036 | 64525 | 415 | 57.21 | 98.39 | 94.36 | 90.09 |
| N2 | 80067 | 78470 | 74476 | 416 | 56.91 | 98.34 | 94.22 | 93.02 |
| N3 | 80072 | 78287 | 75671 | 417 | 56.99 | 98.33 | 94.19 | 94.5 |
| N4 | 79915 | 78085 | 74434 | 416 | 57.18 | 98.38 | 94.33 | 93.14 |
| N5 | 79731 | 78087 | 74887 | 417 | 56.58 | 98.32 | 94.16 | 93.92 |
| O1 | 79783 | 77734 | 74412 | 418 | 56.78 | 98.31 | 94.17 | 93.27 |
| O2 | 79972 | 78265 | 72299 | 417 | 57.19 | 98.38 | 94.33 | 90.41 |
| O3 | 80076 | 78438 | 75326 | 418 | 57.31 | 98.39 | 94.36 | 94.07 |
| O4 | 80026 | 77919 | 75679 | 417 | 57.42 | 98.34 | 94.26 | 94.57 |
| O5 | 79717 | 77257 | 72399 | 418 | 57.05 | 98.27 | 94.06 | 90.82 |
| P1 | 79792 | 77147 | 72767 | 419 | 57.76 | 98.31 | 94.17 | 91.2 |
| P2 | 79973 | 78065 | 71612 | 417 | 57.06 | 98.36 | 94.27 | 89.55 |
| P3 | 80179 | 77543 | 75408 | 420 | 57.67 | 98.24 | 93.99 | 94.05 |
| P4 | 80005 | 77543 | 75130 | 419 | 57.59 | 98.24 | 93.98 | 93.91 |
| P5 | 80265 | 77703 | 75732 | 420 | 57.57 | 98.26 | 94.05 | 94.35 |
| Total | 7538004 | 6677506 | 6210381 | - | - | - | - | - |

A, E, I, M: soil treated with R. palustris PSB-06 and dazomet (T1); B, F, J, N: soil treated with dazomet alone (T2); C, G, K, O: soil treated with R. palustris PSB-06 alone (T3); D, H, L, P: soil without treatment (blank control, T4); A-D: germination stage; E-H: seedling stage ; I-J: vigorous growth stage; M-P: harvest stage.

**Table S2. Statistics of fungal sample sequencing data.**

| Sample ID | Raw Sequences | Clean Sequences | Effective Sequences | AvgLen(bp) | GC(%) | Q20(%) | Q30(%) | Effective(%) |
| --- | --- | --- | --- | --- | --- | --- | --- | --- |
| A1 | 80073 | 78614 | 78046 | 243 | 50.63 | 99.86 | 99.21 | 97.47 |
| A2 | 79864 | 78318 | 78086 | 246 | 50.6 | 99.85 | 99.17 | 97.77 |
| A3 | 79997 | 78521 | 76040 | 245 | 51.41 | 99.89 | 99.29 | 95.05 |
| A4 | 80019 | 78115 | 77205 | 248 | 50.59 | 99.84 | 99.11 | 96.48 |
| A5 | 79799 | 78189 | 77095 | 244 | 52.46 | 99.88 | 99.26 | 96.61 |
| B1 | 80226 | 78802 | 78309 | 244 | 49.65 | 99.84 | 99.16 | 97.61 |
| B2 | 79711 | 77884 | 77671 | 255 | 51.73 | 99.75 | 98.75 | 97.44 |
| B3 | 79981 | 78353 | 78099 | 249 | 52.53 | 99.86 | 99.15 | 97.65 |
| B4 | 79819 | 78087 | 77072 | 253 | 51.75 | 99.83 | 99.02 | 96.56 |
| B5 | 79749 | 78225 | 77947 | 250 | 48.85 | 99.83 | 99.07 | 97.74 |
| C1 | 80146 | 78444 | 76247 | 248 | 52.85 | 99.87 | 99.19 | 95.14 |
| C2 | 79930 | 78396 | 77690 | 245 | 51.56 | 99.88 | 99.24 | 97.2 |
| C3 | 80154 | 78561 | 77826 | 247 | 51.18 | 99.86 | 99.18 | 97.1 |
| C4 | 79972 | 78275 | 77693 | 248 | 51.38 | 99.86 | 99.16 | 97.15 |
| C5 | 80022 | 78287 | 77702 | 247 | 52.09 | 99.87 | 99.19 | 97.1 |
| D1 | 80071 | 78639 | 78373 | 248 | 50.66 | 99.84 | 99.1 | 97.88 |
| D2 | 79984 | 78641 | 78372 | 263 | 47.44 | 99.77 | 98.76 | 97.98 |
| D3 | 79739 | 78421 | 77224 | 254 | 48.82 | 99.84 | 99.07 | 96.85 |
| D4 | 79863 | 78417 | 77453 | 250 | 47.64 | 99.85 | 99.13 | 96.98 |
| D5 | 80006 | 78486 | 77906 | 277 | 47.74 | 99.63 | 98.15 | 97.38 |
| E1 | 60803 | 59900 | 59687 | 243 | 48.88 | 99.84 | 99.12 | 98.16 |
| E2 | 79929 | 78697 | 78678 | 239 | 47.4 | 99.86 | 99.16 | 98.43 |
| E3 | 80074 | 78822 | 78195 | 247 | 46.74 | 99.83 | 99.04 | 97.65 |
| E4 | 80237 | 79075 | 78447 | 237 | 46.24 | 99.87 | 99.25 | 97.77 |
| E5 | 79931 | 78752 | 78602 | 263 | 45.07 | 99.76 | 98.72 | 98.34 |
| F1 | 79873 | 78661 | 78442 | 245 | 47.78 | 99.85 | 99.11 | 98.21 |
| F2 | 49129 | 48428 | 47912 | 245 | 47.49 | 99.83 | 99.06 | 97.52 |
| F3 | 80156 | 79030 | 78819 | 248 | 46.36 | 99.84 | 99.1 | 98.33 |
| F4 | 80226 | 78921 | 78237 | 234 | 48.68 | 99.89 | 99.35 | 97.52 |
| F5 | 80137 | 78985 | 78065 | 259 | 46.8 | 99.74 | 98.68 | 97.41 |
| G1 | 80076 | 78935 | 78794 | 252 | 47 | 99.81 | 98.95 | 98.4 |
| G2 | 79956 | 78733 | 78634 | 246 | 46.84 | 99.81 | 98.89 | 98.35 |
| G3 | 43300 | 42636 | 42314 | 234 | 46.94 | 99.89 | 99.32 | 97.72 |
| G4 | 80444 | 79334 | 79108 | 242 | 44.62 | 99.86 | 99.19 | 98.34 |
| G5 | 80109 | 78749 | 78076 | 270 | 46.71 | 99.71 | 98.51 | 97.46 |
| H1 | 77419 | 76199 | 75252 | 234 | 49.64 | 99.88 | 99.29 | 97.2 |
| H2 | 79917 | 78723 | 77808 | 235 | 49.42 | 99.87 | 99.27 | 97.36 |
| H3 | 47915 | 47160 | 46673 | 234 | 47.88 | 99.88 | 99.29 | 97.41 |
| H4 | 79833 | 78781 | 77042 | 263 | 47.99 | 99.81 | 98.91 | 96.5 |
| H5 | 79885 | 78673 | 78282 | 267 | 48.46 | 99.79 | 98.83 | 97.99 |
| I1 | 80057 | 79000 | 78957 | 262 | 47.95 | 99.82 | 98.9 | 98.63 |
| I2 | 80222 | 79225 | 78811 | 243 | 48.69 | 99.9 | 99.36 | 98.24 |
| I3 | 79894 | 78920 | 78902 | 250 | 44.39 | 99.86 | 99.15 | 98.76 |
| I4 | 80215 | 78986 | 78592 | 245 | 44.53 | 99.86 | 99.17 | 97.98 |
| I5 | 79781 | 78757 | 78709 | 239 | 45.36 | 99.91 | 99.44 | 98.66 |
| J1 | 80138 | 79011 | 78994 | 267 | 41.35 | 99.75 | 98.56 | 98.57 |
| J2 | 79853 | 78897 | 78839 | 255 | 46.64 | 99.84 | 99.02 | 98.73 |
| J3 | 79871 | 78753 | 78586 | 254 | 45.99 | 99.84 | 99.07 | 98.39 |
| J4 | 80029 | 78879 | 78217 | 239 | 44.75 | 99.88 | 99.31 | 97.74 |
| J5 | 80039 | 79064 | 78873 | 251 | 44.43 | 99.85 | 99.07 | 98.54 |
| K1 | 79976 | 78594 | 78438 | 257 | 42 | 99.77 | 98.65 | 98.08 |
| K2 | 79934 | 78740 | 77654 | 237 | 47.81 | 99.9 | 99.35 | 97.15 |
| K3 | 79917 | 78697 | 78400 | 244 | 46.75 | 99.85 | 99.13 | 98.1 |
| K4 | 79823 | 78665 | 78318 | 235 | 44.67 | 99.9 | 99.41 | 98.11 |
| K5 | 79965 | 78707 | 77567 | 249 | 46.43 | 99.82 | 98.99 | 97 |
| L1 | 79871 | 78497 | 77616 | 245 | 45.83 | 99.85 | 99.12 | 97.18 |
| L2 | 80035 | 78965 | 78959 | 240 | 46.64 | 99.91 | 99.4 | 98.66 |
| L3 | 68486 | 67556 | 67112 | 231 | 48.86 | 99.9 | 99.39 | 97.99 |
| L4 | 80336 | 79039 | 78953 | 267 | 43.52 | 99.79 | 98.73 | 98.28 |
| L5 | 79972 | 78865 | 78300 | 244 | 47.31 | 99.85 | 99.15 | 97.91 |
| M1 | 79911 | 78819 | 78142 | 250 | 46.67 | 99.83 | 99.05 | 97.79 |
| M2 | 79990 | 78802 | 78083 | 244 | 46.12 | 99.87 | 99.2 | 97.62 |
| M3 | 79939 | 78812 | 78258 | 235 | 48.69 | 99.89 | 99.35 | 97.9 |
| M4 | 79607 | 78437 | 77970 | 238 | 48.11 | 99.89 | 99.32 | 97.94 |
| M5 | 68610 | 67637 | 67161 | 232 | 47.94 | 99.9 | 99.41 | 97.89 |
| N1 | 79639 | 78446 | 78113 | 233 | 48.6 | 99.9 | 99.38 | 98.08 |
| N2 | 80367 | 79191 | 78596 | 232 | 48.53 | 99.9 | 99.38 | 97.8 |
| N3 | 80027 | 78804 | 78409 | 232 | 48.59 | 99.89 | 99.36 | 97.98 |
| N4 | 80054 | 79009 | 78543 | 232 | 48.32 | 99.91 | 99.44 | 98.11 |
| N5 | 78728 | 77532 | 76925 | 234 | 48.51 | 99.89 | 99.33 | 97.71 |
| O1 | 79983 | 78856 | 78477 | 230 | 48.12 | 99.9 | 99.42 | 98.12 |
| O2 | 80020 | 78778 | 78416 | 233 | 48.96 | 99.9 | 99.4 | 98 |
| O3 | 80017 | 78825 | 78331 | 231 | 48.58 | 99.9 | 99.39 | 97.89 |
| O4 | 80146 | 78991 | 78328 | 233 | 48.74 | 99.9 | 99.37 | 97.73 |
| O5 | 73413 | 72269 | 71973 | 234 | 47.85 | 99.89 | 99.33 | 98.04 |
| P1 | 60169 | 59241 | 58839 | 229 | 48.38 | 99.9 | 99.42 | 97.79 |
| P2 | 56022 | 55271 | 55063 | 231 | 47.18 | 99.9 | 99.41 | 98.29 |
| P3 | 50253 | 49497 | 49106 | 228 | 48.11 | 99.91 | 99.41 | 97.72 |
| P4 | 53449 | 52643 | 52334 | 232 | 48.23 | 99.9 | 99.38 | 97.91 |
| P5 | 67434 | 66539 | 66171 | 230 | 47.7 | 99.91 | 99.43 | 98.13 |
| Total | 6134666 | 6037085 | 5995158 | - | - | - | - | - |

A, E, I, M: soil treated with R. palustris PSB-06 and dazomet (T1); B, F, J, N: soil treated with dazomet alone (T2); C, G, K, O: soil treated with R. palustris PSB-06 alone (T3); D, H, L, P: soil without treatment (blank control, T4); A-D: germination stage; E-H: seedling stage ; I-J: vigorous growth stage; M-P: harvest stage.

**Table S3. The OTUs in bacterial and fungal samples.**

|  | Bacteria | | Fungi | |
| --- | --- | --- | --- | --- |
| BMK_ID | OTU_Num | Seqs_Num | OTU_Num | Seqs_Num |
| A1 | 1308 | 75329 | 480 | 67185 |
| A2 | 1268 | 69759 | 256 | 76704 |
| A3 | 1308 | 58636 | 253 | 74645 |
| A4 | 1367 | 72758 | 320 | 74968 |
| A5 | 1386 | 78297 | 208 | 76457 |
| B1 | 1296 | 83843 | 239 | 76620 |
| B2 | 1181 | 33105 | 227 | 76238 |
| B3 | 1283 | 64900 | 236 | 77252 |
| B4 | 1278 | 54382 | 230 | 75622 |
| B5 | 1263 | 57065 | 292 | 74468 |
| C1 | 1292 | 50774 | 230 | 75266 |
| C2 | 1402 | 65305 | 255 | 76071 |
| C3 | 1238 | 49237 | 250 | 75452 |
| C4 | 1434 | 70631 | 249 | 75468 |
| C5 | 1438 | 70182 | 255 | 76365 |
| D1 | 1289 | 75501 | 254 | 74548 |
| D2 | 1454 | 46314 | 260 | 76844 |
| D3 | 1467 | 59602 | 257 | 75320 |
| D4 | 1446 | 48367 | 255 | 76001 |
| D5 | 1385 | 39240 | 283 | 75266 |
| E1 | 1402 | 60096 | 567 | 48459 |
| E2 | 1351 | 59236 | 320 | 74151 |
| E3 | 1405 | 60016 | 416 | 70992 |
| E4 | 1293 | 57876 | 403 | 67990 |
| E5 | 1476 | 60267 | 426 | 75160 |
| F1 | 1409 | 57553 | 463 | 65357 |
| F2 | 1423 | 62250 | 389 | 44225 |
| F3 | 1444 | 59113 | 429 | 71949 |
| F4 | 1447 | 59336 | 311 | 72459 |
| F5 | 1421 | 62646 | 352 | 73785 |
| G1 | 1513 | 56733 | 273 | 74738 |
| G2 | 1508 | 51359 | 314 | 72725 |
| G3 | 1369 | 62288 | 356 | 37841 |
| G4 | 1478 | 54735 | 322 | 77040 |
| G5 | 1453 | 60428 | 369 | 73177 |
| H1 | 1447 | 53258 | 596 | 65833 |
| H2 | 1417 | 53590 | 556 | 62668 |
| H3 | 1468 | 55198 | 356 | 43772 |
| H4 | 1386 | 62772 | 327 | 73779 |
| H5 | 1481 | 58835 | 444 | 72194 |
| I1 | 1505 | 60012 | 307 | 78175 |
| I2 | 1561 | 58737 | 401 | 75457 |
| I3 | 1468 | 58388 | 341 | 77652 |
| I4 | 1502 | 58704 | 387 | 76796 |
| I5 | 1475 | 55657 | 372 | 77874 |
| J1 | 1395 | 60061 | 337 | 77120 |
| J2 | 1449 | 61672 | 314 | 77721 |
| J3 | 1332 | 63233 | 349 | 77128 |
| J4 | 1495 | 60662 | 415 | 72107 |
| J5 | 1473 | 59750 | 339 | 76480 |
| K1 | 1461 | 59743 | 405 | 75461 |
| K2 | 1432 | 59426 | 415 | 65106 |
| K3 | 1401 | 61632 | 370 | 73654 |
| K4 | 1448 | 35226 | 371 | 75879 |
| K5 | 1458 | 55561 | 359 | 72881 |
| L1 | 1404 | 51696 | 380 | 72569 |
| L2 | 1441 | 54019 | 334 | 76994 |
| L3 | 1473 | 54404 | 721 | 65049 |
| L4 | 1522 | 52207 | 449 | 77456 |
| L5 | 1435 | 58626 | 518 | 71396 |
| M1 | 1166 | 55408 | 433 | 71469 |
| M2 | 1361 | 58577 | 537 | 70379 |
| M3 | 1274 | 55985 | 598 | 76334 |
| M4 | 1503 | 53108 | 604 | 76614 |
| M5 | 1423 | 54286 | 586 | 65910 |
| N1 | 1408 | 37023 | 558 | 76559 |
| N2 | 1497 | 50829 | 575 | 76956 |
| N3 | 1119 | 59839 | 561 | 76912 |
| N4 | 1436 | 58808 | 575 | 76882 |
| N5 | 1445 | 57706 | 561 | 75572 |
| O1 | 1599 | 57130 | 572 | 77152 |
| O2 | 1490 | 48723 | 581 | 77381 |
| O3 | 1465 | 53676 | 603 | 76356 |
| O4 | 1536 | 57405 | 624 | 76421 |
| O5 | 1540 | 51680 | 605 | 70614 |
| P1 | 1334 | 36964 | 589 | 56796 |
| P2 | 1509 | 39760 | 591 | 53975 |
| P3 | 1400 | 55641 | 568 | 48210 |
| P4 | 1443 | 53511 | 577 | 51140 |
| P5 | 1430 | 53800 | 585 | 64645 |
| Total | 1833 | 4580087 | 1069 | 5714286 |

A, E, I, M: soil treated with R. palustris PSB-06 and dazomet (T1); B, F, J, N: soil treated with dazomet alone (T2); C, G, K, O: soil treated with R. palustris PSB-06 alone (T3); D, H, L, P: soil without treatment (blank control, T4); A-D: germination stage; E-H: seedling stage ; I-J: vigorous growth stage; M-P: harvest stage.

**Table S4. The statistics of alpha diversity indices of soil bacteria.**

| Sample ID | Feature | ACE | Chao1 | PD_whole_tree | Coverage | Simpson | Shannon |
| --- | --- | --- | --- | --- | --- | --- | --- |
| A1 | 1308 | 1498.455 | 1528.745 | 66.6246 | 0.9967 | 0.9713 | 6.885 |
| A2 | 1268 | 1444.871 | 1460.169 | 66.328 | 0.9966 | 0.9645 | 6.6332 |
| A3 | 1308 | 1479.683 | 1487.708 | 66.7699 | 0.9961 | 0.9837 | 7.6528 |
| A4 | 1367 | 1546.156 | 1632.321 | 67.8931 | 0.9967 | 0.9819 | 7.5274 |
| A5 | 1386 | 1517.363 | 1521.438 | 70.4132 | 0.9975 | 0.9827 | 7.5718 |
| B1 | 1296 | 1472.264 | 1471.273 | 66.2952 | 0.9971 | 0.9715 | 6.7067 |
| B2 | 1181 | 1408.806 | 1448.15 | 63.5422 | 0.9917 | 0.9801 | 7.3302 |
| B3 | 1283 | 1481.116 | 1528.578 | 66.626 | 0.996 | 0.9765 | 7.0964 |
| B4 | 1278 | 1432.825 | 1460.609 | 66.4917 | 0.9959 | 0.9767 | 7.2578 |
| B5 | 1263 | 1418.081 | 1409.913 | 65.769 | 0.9962 | 0.9794 | 7.2441 |
| C1 | 1292 | 1461.559 | 1523.437 | 66.0998 | 0.9952 | 0.98 | 7.3505 |
| C2 | 1402 | 1525.023 | 1564.097 | 70.9604 | 0.9969 | 0.9845 | 7.6988 |
| C3 | 1238 | 1416.005 | 1413.927 | 64.1203 | 0.9953 | 0.9803 | 7.4177 |
| C4 | 1434 | 1570.607 | 1589.257 | 71.6197 | 0.997 | 0.9776 | 7.4916 |
| C5 | 1438 | 1583.368 | 1616.928 | 72.0574 | 0.997 | 0.9862 | 7.9957 |
| D1 | 1289 | 1455.409 | 1477.007 | 66.4245 | 0.9969 | 0.9693 | 6.6698 |
| D2 | 1454 | 1547.799 | 1564.5 | 71.1085 | 0.9963 | 0.9901 | 8.5238 |
| D3 | 1467 | 1559.618 | 1574.084 | 73.0805 | 0.9972 | 0.989 | 8.2328 |
| D4 | 1446 | 1575.963 | 1640.204 | 70.2133 | 0.9957 | 0.9908 | 8.4408 |
| D5 | 1385 | 1500.336 | 1533.224 | 68.963 | 0.9951 | 0.9911 | 8.6342 |
| E1 | 1402 | 1473.289 | 1499.042 | 72.0989 | 0.9977 | 0.9938 | 8.704 |
| E2 | 1351 | 1435.01 | 1480.5 | 70.8595 | 0.9975 | 0.9938 | 8.6045 |
| E3 | 1405 | 1483.624 | 1516.064 | 73.0043 | 0.9976 | 0.9942 | 8.7173 |
| E4 | 1293 | 1379.262 | 1396.6 | 68.4146 | 0.9974 | 0.9933 | 8.495 |
| E5 | 1476 | 1540.694 | 1559.75 | 73.7945 | 0.9978 | 0.9936 | 8.7115 |
| F1 | 1409 | 1465.692 | 1491 | 72.9248 | 0.9978 | 0.9942 | 8.6966 |
| F2 | 1423 | 1488.75 | 1503.804 | 71.9858 | 0.9979 | 0.9935 | 8.5757 |
| F3 | 1444 | 1516.585 | 1536.974 | 74.0249 | 0.9975 | 0.9927 | 8.5376 |
| F4 | 1447 | 1533.986 | 1573.612 | 72.8651 | 0.9973 | 0.9928 | 8.6016 |
| F5 | 1421 | 1487.565 | 1529.111 | 72.8057 | 0.9978 | 0.9937 | 8.6027 |
| G1 | 1513 | 1585.318 | 1611 | 76.2653 | 0.9974 | 0.993 | 8.6753 |
| G2 | 1508 | 1574.796 | 1594.778 | 75.4973 | 0.9972 | 0.9937 | 8.7863 |
| G3 | 1369 | 1447.071 | 1471.093 | 71.1783 | 0.9976 | 0.993 | 8.4082 |
| G4 | 1478 | 1567.652 | 1604.009 | 74.818 | 0.9969 | 0.9933 | 8.7025 |
| G5 | 1453 | 1543.321 | 1590.238 | 74.4906 | 0.9972 | 0.9931 | 8.5688 |
| H1 | 1447 | 1535.877 | 1561.395 | 73.0179 | 0.997 | 0.9931 | 8.7533 |
| H2 | 1417 | 1507.344 | 1527.741 | 72.6712 | 0.9971 | 0.994 | 8.7974 |
| H3 | 1468 | 1539.628 | 1555.526 | 73.3248 | 0.9974 | 0.9934 | 8.7506 |
| H4 | 1386 | 1449.828 | 1476.063 | 71.1003 | 0.9979 | 0.993 | 8.4692 |
| H5 | 1481 | 1558.269 | 1593.594 | 74.6803 | 0.9974 | 0.9915 | 8.4733 |
| I1 | 1505 | 1597.244 | 1628.2 | 75.0296 | 0.9971 | 0.9898 | 8.2189 |
| I2 | 1561 | 1644.109 | 1687.449 | 76.6332 | 0.9972 | 0.9923 | 8.6464 |
| I3 | 1468 | 1565.612 | 1587.38 | 74.2224 | 0.997 | 0.9924 | 8.4357 |
| I4 | 1502 | 1568.747 | 1589.991 | 75.9927 | 0.9976 | 0.9937 | 8.7453 |
| I5 | 1475 | 1578.85 | 1599.221 | 73.8821 | 0.9966 | 0.9903 | 8.3242 |
| J1 | 1395 | 1467.142 | 1477.057 | 72.6175 | 0.9976 | 0.9902 | 8.3245 |
| J2 | 1449 | 1508.412 | 1550.024 | 73.421 | 0.9979 | 0.994 | 8.7032 |
| J3 | 1332 | 1391.294 | 1415.367 | 69.0172 | 0.9981 | 0.9923 | 8.3615 |
| J4 | 1495 | 1554.29 | 1583.724 | 73.9113 | 0.9977 | 0.9921 | 8.5412 |
| J5 | 1473 | 1536.723 | 1571.876 | 74.1263 | 0.9977 | 0.9926 | 8.5741 |
| K1 | 1461 | 1532.179 | 1558.194 | 75.1898 | 0.9976 | 0.9931 | 8.6157 |
| K2 | 1432 | 1501.308 | 1512.11 | 72.9265 | 0.9977 | 0.9915 | 8.3905 |
| K3 | 1401 | 1494.32 | 1544.72 | 72.0468 | 0.9973 | 0.9888 | 8.1757 |
| K4 | 1448 | 1556.872 | 1568.781 | 71.5744 | 0.9945 | 0.9936 | 8.7105 |
| K5 | 1458 | 1517.793 | 1544.888 | 74.6325 | 0.9976 | 0.9907 | 8.4559 |
| L1 | 1404 | 1488.913 | 1504.786 | 69.8198 | 0.9968 | 0.99 | 8.3681 |
| L2 | 1441 | 1521.78 | 1544.358 | 73.4821 | 0.9971 | 0.9887 | 8.3912 |
| L3 | 1473 | 1546.476 | 1556.836 | 74.5218 | 0.9973 | 0.9938 | 8.7124 |
| L4 | 1522 | 1604.023 | 1618.425 | 75.4375 | 0.997 | 0.9923 | 8.7578 |
| L5 | 1435 | 1531.055 | 1561.437 | 72.7908 | 0.9969 | 0.982 | 7.9058 |
| M1 | 1166 | 1412.453 | 1448.485 | 62.7268 | 0.9957 | 0.9884 | 7.8762 |
| M2 | 1361 | 1432.813 | 1485.382 | 70.5093 | 0.9976 | 0.9924 | 8.5414 |
| M3 | 1274 | 1368.421 | 1413.745 | 66.7244 | 0.997 | 0.9825 | 7.7326 |
| M4 | 1503 | 1562.748 | 1595.823 | 74.1302 | 0.9975 | 0.9929 | 8.8448 |
| M5 | 1423 | 1509.39 | 1528.244 | 70.6481 | 0.997 | 0.99 | 8.2648 |
| N1 | 1408 | 1559.913 | 1597.174 | 70.2334 | 0.9938 | 0.9935 | 8.6464 |
| N2 | 1497 | 1583.887 | 1628.429 | 75.1874 | 0.9968 | 0.9954 | 8.968 |
| N3 | 1119 | 1271.029 | 1328.262 | 61.2925 | 0.9969 | 0.9874 | 7.733 |
| N4 | 1436 | 1526.963 | 1559.983 | 73.3873 | 0.9971 | 0.9892 | 8.1857 |
| N5 | 1445 | 1528.719 | 1556.579 | 73.3439 | 0.9972 | 0.9905 | 8.3029 |
| O1 | 1599 | 1658.595 | 1681.2 | 79.3978 | 0.9976 | 0.9905 | 8.6682 |
| O2 | 1490 | 1587.51 | 1632.333 | 73.0531 | 0.9962 | 0.9934 | 8.5917 |
| O3 | 1465 | 1553.549 | 1595.591 | 74.1547 | 0.9968 | 0.9926 | 8.53 |
| O4 | 1536 | 1588.037 | 1602 | 75.4722 | 0.9979 | 0.9926 | 8.7438 |
| O5 | 1540 | 1619.387 | 1628.593 | 75.3484 | 0.9969 | 0.9926 | 8.6993 |
| P1 | 1334 | 1454.017 | 1480.826 | 68.5379 | 0.9949 | 0.9922 | 8.6602 |
| P2 | 1509 | 1583.862 | 1594.5 | 74.1768 | 0.9962 | 0.9912 | 8.7853 |
| P3 | 1400 | 1484.151 | 1501.116 | 69.7726 | 0.9973 | 0.9894 | 8.4504 |
| P4 | 1443 | 1512.544 | 1519.992 | 72.1474 | 0.9974 | 0.9925 | 8.73 |
| P5 | 1430 | 1526.856 | 1559.198 | 71.7204 | 0.9969 | 0.9927 | 8.7361 |

A, E, I, M: soil treated with R. palustris PSB-06 and dazomet (T1); B, F, J, N: soil treated with dazomet alone (T2); C, G, K, O: soil treated with R. palustris PSB-06 alone (T3); D, H, L, P: soil without treatment (blank control, T4); A-D: germination stage; E-H: seedling stage ; I-J: vigorous growth stage; M-P: harvest stage.

**Table S5. The statistics of alpha diversity indices of soil fungi.**

| Sample ID | Feature | ACE | Chao1 | PD_whole_tree | Coverage | Simpson | Shannon |
| --- | --- | --- | --- | --- | --- | --- | --- |
| A1 | 480 | 519.3094 | 549.7895 | 91.8218 | 0.9992 | 0.9517 | 6.1295 |
| A2 | 256 | 384.9202 | 358.7895 | 51.1297 | 0.9992 | 0.8984 | 4.4992 |
| A3 | 253 | 383.2165 | 371.5882 | 53.7827 | 0.9991 | 0.797 | 3.8106 |
| A4 | 320 | 362.9511 | 430.0769 | 61.0662 | 0.9993 | 0.8827 | 4.4124 |
| A5 | 208 | 358.1722 | 314.9375 | 45.3027 | 0.9992 | 0.7553 | 3.0859 |
| B1 | 239 | 387.6822 | 358.25 | 52.6361 | 0.9993 | 0.9112 | 4.8185 |
| B2 | 227 | 409.3348 | 300.6667 | 50.4659 | 0.9993 | 0.8556 | 4.4878 |
| B3 | 236 | 352.7926 | 386.2727 | 51.8611 | 0.9992 | 0.7614 | 3.5889 |
| B4 | 230 | 313.2417 | 290.5455 | 49.262 | 0.9995 | 0.9097 | 4.6671 |
| B5 | 292 | 374.8049 | 362.7143 | 57.1151 | 0.9994 | 0.9574 | 5.4795 |
| C1 | 230 | 276.3586 | 294.6875 | 49.0549 | 0.9994 | 0.7668 | 3.6693 |
| C2 | 255 | 394.8957 | 346.8333 | 56.0463 | 0.9992 | 0.7768 | 3.9452 |
| C3 | 250 | 362.1668 | 349.75 | 53.6521 | 0.9992 | 0.9182 | 4.6989 |
| C4 | 249 | 347.6397 | 343 | 55.0522 | 0.9994 | 0.9302 | 5.0003 |
| C5 | 255 | 388.4501 | 373.0714 | 55.9052 | 0.9992 | 0.6942 | 3.5037 |
| D1 | 254 | 404.0372 | 332.1579 | 54.0071 | 0.9993 | 0.9664 | 5.4756 |
| D2 | 260 | 364.5198 | 325.3333 | 57.4444 | 0.9994 | 0.9271 | 4.7728 |
| D3 | 257 | 416.2859 | 385.3333 | 55.7425 | 0.9993 | 0.9379 | 5.1226 |
| D4 | 255 | 330.7346 | 325.7143 | 55.6849 | 0.9994 | 0.9284 | 4.759 |
| D5 | 283 | 291.0738 | 294.7692 | 60.4965 | 0.9998 | 0.9176 | 4.7504 |
| E1 | 567 | 609.6363 | 628.4167 | 107.3992 | 0.9986 | 0.9821 | 7.118 |
| E2 | 320 | 702.1083 | 535 | 72.7971 | 0.9988 | 0.958 | 6.1852 |
| E3 | 416 | 905.9458 | 620.129 | 86.56 | 0.9984 | 0.9679 | 6.6534 |
| E4 | 403 | 985.3518 | 645.7778 | 85.7779 | 0.9983 | 0.9653 | 6.4638 |
| E5 | 426 | 1029.327 | 664 | 88.607 | 0.9984 | 0.9127 | 5.1964 |
| F1 | 463 | 675.1938 | 615.5 | 91.0195 | 0.9991 | 0.9898 | 7.6631 |
| F2 | 389 | 696.0423 | 505.4348 | 85.4811 | 0.9976 | 0.9877 | 7.0484 |
| F3 | 429 | 649.3924 | 532.5455 | 91.0527 | 0.9991 | 0.8453 | 4.7641 |
| F4 | 311 | 713.3377 | 457.625 | 68.1065 | 0.999 | 0.9819 | 6.7517 |
| F5 | 352 | 687.4399 | 467 | 80.0506 | 0.9991 | 0.9456 | 6.0446 |
| G1 | 273 | 795.337 | 443.0769 | 66.432 | 0.9991 | 0.972 | 6.3162 |
| G2 | 314 | 887.982 | 532 | 74.5722 | 0.9985 | 0.9442 | 5.7656 |
| G3 | 356 | 917.9833 | 624.8889 | 81.0336 | 0.9968 | 0.9792 | 6.4843 |
| G4 | 322 | 674.2836 | 542.0588 | 78.3834 | 0.9989 | 0.9289 | 5.2179 |
| G5 | 369 | 1118.82 | 636.9643 | 88.4988 | 0.9983 | 0.8905 | 5.4054 |
| H1 | 596 | 657.1511 | 720.6154 | 113.8117 | 0.9988 | 0.9759 | 6.7995 |
| H2 | 556 | 622.6678 | 670.2609 | 103.5521 | 0.9988 | 0.9687 | 6.6491 |
| H3 | 356 | 1068.786 | 676.913 | 80.7978 | 0.9972 | 0.9749 | 6.4869 |
| H4 | 327 | 843.9591 | 532.0588 | 75.3698 | 0.9989 | 0.858 | 5.0995 |
| H5 | 444 | 719.2457 | 611.5714 | 94.9269 | 0.999 | 0.7622 | 4.4279 |
| I1 | 307 | 545.1715 | 443.12 | 74.8826 | 0.9989 | 0.7381 | 3.075 |
| I2 | 401 | 556.805 | 511.0357 | 80.059 | 0.999 | 0.9044 | 4.7971 |
| I3 | 341 | 522.7488 | 489.3333 | 75.4255 | 0.9988 | 0.877 | 4.1386 |
| I4 | 387 | 978.0692 | 590.5 | 88.972 | 0.9986 | 0.9519 | 5.6845 |
| I5 | 372 | 620.863 | 547.5263 | 80.2385 | 0.9985 | 0.823 | 3.7246 |
| J1 | 337 | 663.724 | 587.1429 | 78.7066 | 0.9987 | 0.8482 | 3.7832 |
| J2 | 314 | 605.5039 | 496.2857 | 72.7646 | 0.9989 | 0.8621 | 3.7622 |
| J3 | 349 | 964.4433 | 577.3913 | 75.8711 | 0.9987 | 0.9153 | 4.9165 |
| J4 | 415 | 610.3073 | 575 | 92.142 | 0.9991 | 0.9186 | 5.684 |
| J5 | 339 | 723.7798 | 577 | 81.6683 | 0.9989 | 0.8955 | 4.5954 |
| K1 | 405 | 913.6553 | 655.3125 | 88.6206 | 0.9988 | 0.9072 | 5.4139 |
| K2 | 415 | 714.262 | 535.75 | 93.0667 | 0.9989 | 0.9792 | 6.6482 |
| K3 | 370 | 783.6473 | 511.7778 | 86.3336 | 0.9988 | 0.9861 | 7.0005 |
| K4 | 371 | 775.7855 | 509.1034 | 83.8633 | 0.9988 | 0.7711 | 4.5784 |
| K5 | 359 | 820.3674 | 533 | 82.4311 | 0.9988 | 0.9864 | 7.0323 |
| L1 | 380 | 870.4447 | 583.7143 | 86.2707 | 0.9987 | 0.9626 | 6.225 |
| L2 | 334 | 578.6069 | 547.4615 | 73.8793 | 0.999 | 0.8607 | 4.4183 |
| L3 | 721 | 775.8133 | 798.025 | 130.8479 | 0.9988 | 0.9779 | 7.1958 |
| L4 | 449 | 505.7969 | 572.24 | 96.4916 | 0.999 | 0.6662 | 3.3998 |
| L5 | 518 | 740.5501 | 636.3333 | 101.342 | 0.999 | 0.9683 | 6.7306 |
| M1 | 433 | 794.6594 | 664.4286 | 93.3782 | 0.9989 | 0.9732 | 6.6814 |
| M2 | 537 | 674.3876 | 665.3333 | 107.1039 | 0.9992 | 0.9846 | 7.3131 |
| M3 | 598 | 625.345 | 648.1667 | 115.5727 | 0.9994 | 0.9854 | 7.3861 |
| M4 | 604 | 659.6518 | 679.64 | 117.5946 | 0.9992 | 0.9854 | 7.3316 |
| M5 | 586 | 616.4077 | 625.6 | 113.703 | 0.9993 | 0.9833 | 7.2592 |
| N1 | 558 | 591.2144 | 610.5556 | 107.9823 | 0.9994 | 0.9845 | 7.3344 |
| N2 | 575 | 603.542 | 657.0909 | 107.9052 | 0.9994 | 0.9847 | 7.3808 |
| N3 | 561 | 591.8717 | 606.3158 | 109.8084 | 0.9995 | 0.9817 | 7.2173 |
| N4 | 575 | 598.612 | 621.3125 | 111.3835 | 0.9995 | 0.9782 | 7.0594 |
| N5 | 561 | 583.5204 | 653.625 | 108.9156 | 0.9995 | 0.9842 | 7.3216 |
| O1 | 572 | 626.3576 | 657.5556 | 111.0093 | 0.9993 | 0.9839 | 7.3477 |
| O2 | 581 | 605.1496 | 609.12 | 110.6919 | 0.9995 | 0.9858 | 7.4005 |
| O3 | 603 | 625.9163 | 625.8846 | 114.8429 | 0.9995 | 0.9833 | 7.3833 |
| O4 | 624 | 643.3367 | 659.2857 | 116.9255 | 0.9995 | 0.9858 | 7.4475 |
| O5 | 605 | 632.8665 | 657.5556 | 116.2014 | 0.9994 | 0.9847 | 7.4044 |
| P1 | 589 | 609.6877 | 630.0526 | 112.9696 | 0.9993 | 0.9843 | 7.3932 |
| P2 | 591 | 614.2744 | 617.4516 | 116.6169 | 0.9992 | 0.982 | 7.23 |
| P3 | 568 | 589.2379 | 601.913 | 108.9313 | 0.9992 | 0.9822 | 7.2734 |
| P4 | 577 | 607.3056 | 624.1154 | 109.1273 | 0.999 | 0.9855 | 7.4234 |
| P5 | 585 | 607.5788 | 608.4333 | 113.6149 | 0.9994 | 0.985 | 7.4291 |

A, E, I, M: soil treated with R. palustris PSB-06 and dazomet (T1); B, F, J, N: soil treated with dazomet alone (T2); C, G, K, O: soil treated with R. palustris PSB-06 alone (T3); D, H, L, P: soil without treatment (blank control, T4); A-D: germination stage; E-H: seedling stage ; I-J: vigorous growth stage; M-P: harvest stage.

**Table S6. The statistics of sequences in each grade of soil bacteria.**

| Sample | Kindom | Phylum | Class | Order | Family | Genus | Species |
| --- | --- | --- | --- | --- | --- | --- | --- |
| A1 | 75,329 | 75,329 | 75,329 | 75,329 | 75,329 | 75,329 | 75,329 |
| A2 | 69,759 | 69,759 | 69,759 | 69,759 | 69,759 | 69,759 | 69,759 |
| A3 | 58,636 | 58,636 | 58,636 | 58,636 | 58,636 | 58,636 | 58,636 |
| A4 | 72,758 | 72,758 | 72,758 | 72,758 | 72,758 | 72,758 | 72,758 |
| A5 | 78,297 | 78,297 | 78,297 | 78,297 | 78,297 | 78,297 | 78,297 |
| B1 | 83,843 | 83,843 | 83,843 | 83,843 | 83,843 | 83,843 | 83,843 |
| B2 | 33,105 | 33,105 | 33,105 | 33,105 | 33,105 | 33,105 | 33,105 |
| B3 | 64,900 | 64,900 | 64,900 | 64,900 | 64,900 | 64,900 | 64,900 |
| B4 | 54,382 | 54,382 | 54,382 | 54,382 | 54,382 | 54,382 | 54,382 |
| B5 | 57,065 | 57,065 | 57,065 | 57,065 | 57,065 | 57,065 | 57,065 |
| C1 | 50,774 | 50,774 | 50,774 | 50,774 | 50,774 | 50,774 | 50,774 |
| C2 | 65,305 | 65,305 | 65,305 | 65,305 | 65,305 | 65,305 | 65,305 |
| C3 | 49,237 | 49,237 | 49,237 | 49,237 | 49,237 | 49,237 | 49,237 |
| C4 | 70,631 | 70,631 | 70,631 | 70,631 | 70,631 | 70,631 | 70,631 |
| C5 | 70,182 | 70,182 | 70,182 | 70,182 | 70,182 | 70,182 | 70,182 |
| D1 | 75,501 | 75,501 | 75,501 | 75,501 | 75,501 | 75,501 | 75,501 |
| D2 | 46,314 | 46,314 | 46,314 | 46,314 | 46,314 | 46,314 | 46,314 |
| D3 | 59,602 | 59,602 | 59,602 | 59,602 | 59,602 | 59,602 | 59,602 |
| D4 | 48,367 | 48,367 | 48,367 | 48,367 | 48,367 | 48,367 | 48,367 |
| D5 | 39,240 | 39,240 | 39,240 | 39,240 | 39,240 | 39,240 | 39,240 |
| E1 | 60,096 | 60,096 | 60,096 | 60,096 | 60,096 | 60,096 | 60,096 |
| E2 | 59,236 | 59,236 | 59,236 | 59,236 | 59,236 | 59,236 | 59,236 |
| E3 | 60,016 | 60,016 | 60,016 | 60,016 | 60,016 | 60,016 | 60,016 |
| E4 | 57,876 | 57,876 | 57,876 | 57,876 | 57,876 | 57,876 | 57,876 |
| E5 | 60,267 | 60,267 | 60,267 | 60,267 | 60,267 | 60,267 | 60,267 |
| F1 | 57,553 | 57,553 | 57,553 | 57,553 | 57,553 | 57,553 | 57,553 |
| F2 | 62,250 | 62,250 | 62,250 | 62,250 | 62,250 | 62,250 | 62,250 |
| F3 | 59,113 | 59,113 | 59,113 | 59,113 | 59,113 | 59,113 | 59,113 |
| F4 | 59,336 | 59,336 | 59,336 | 59,336 | 59,336 | 59,336 | 59,336 |
| F5 | 62,646 | 62,646 | 62,646 | 62,646 | 62,646 | 62,646 | 62,646 |
| G1 | 56,733 | 56,733 | 56,733 | 56,733 | 56,733 | 56,733 | 56,733 |
| G2 | 51,359 | 51,359 | 51,359 | 51,359 | 51,359 | 51,359 | 51,359 |
| G3 | 62,288 | 62,288 | 62,288 | 62,288 | 62,288 | 62,288 | 62,288 |
| G4 | 54,735 | 54,735 | 54,735 | 54,735 | 54,735 | 54,735 | 54,735 |
| G5 | 60,428 | 60,428 | 60,428 | 60,428 | 60,428 | 60,428 | 60,428 |
| H1 | 53,258 | 53,258 | 53,258 | 53,258 | 53,258 | 53,258 | 53,258 |
| H2 | 53,590 | 53,590 | 53,590 | 53,590 | 53,590 | 53,590 | 53,590 |
| H3 | 55,198 | 55,198 | 55,198 | 55,198 | 55,198 | 55,198 | 55,198 |
| H4 | 62,772 | 62,772 | 62,772 | 62,772 | 62,772 | 62,772 | 62,772 |
| H5 | 58,835 | 58,835 | 58,835 | 58,835 | 58,835 | 58,835 | 58,835 |
| I1 | 60,012 | 60,012 | 60,012 | 60,012 | 60,012 | 60,012 | 60,012 |
| I2 | 58,737 | 58,737 | 58,737 | 58,737 | 58,737 | 58,737 | 58,737 |
| I3 | 58,388 | 58,388 | 58,388 | 58,388 | 58,388 | 58,388 | 58,388 |
| I4 | 58,704 | 58,704 | 58,704 | 58,704 | 58,704 | 58,704 | 58,704 |
| I5 | 55,657 | 55,657 | 55,657 | 55,657 | 55,657 | 55,657 | 55,657 |
| J1 | 60,061 | 60,061 | 60,061 | 60,061 | 60,061 | 60,061 | 60,061 |
| J2 | 61,672 | 61,672 | 61,672 | 61,672 | 61,672 | 61,672 | 61,672 |
| J3 | 63,233 | 63,233 | 63,233 | 63,233 | 63,233 | 63,233 | 63,233 |
| J4 | 60,662 | 60,662 | 60,662 | 60,662 | 60,662 | 60,662 | 60,662 |
| J5 | 59,750 | 59,750 | 59,750 | 59,750 | 59,750 | 59,750 | 59,750 |
| K1 | 59,743 | 59,743 | 59,743 | 59,743 | 59,743 | 59,743 | 59,743 |
| K2 | 59,426 | 59,426 | 59,426 | 59,426 | 59,426 | 59,426 | 59,426 |
| K3 | 61,632 | 61,632 | 61,632 | 61,632 | 61,632 | 61,632 | 61,632 |
| K4 | 35,226 | 35,226 | 35,226 | 35,226 | 35,226 | 35,226 | 35,226 |
| K5 | 55,561 | 55,561 | 55,561 | 55,561 | 55,561 | 55,561 | 55,561 |
| L1 | 51,696 | 51,696 | 51,696 | 51,696 | 51,696 | 51,696 | 51,696 |
| L2 | 54,019 | 54,019 | 54,019 | 54,019 | 54,019 | 54,019 | 54,019 |
| L3 | 54,404 | 54,404 | 54,404 | 54,404 | 54,404 | 54,404 | 54,404 |
| L4 | 52,207 | 52,207 | 52,207 | 52,207 | 52,207 | 52,207 | 52,207 |
| L5 | 58,626 | 58,626 | 58,626 | 58,626 | 58,626 | 58,626 | 58,626 |
| M1 | 55,408 | 55,408 | 55,408 | 55,408 | 55,408 | 55,408 | 55,408 |
| M2 | 58,577 | 58,577 | 58,577 | 58,577 | 58,577 | 58,577 | 58,577 |
| M3 | 55,985 | 55,985 | 55,985 | 55,985 | 55,985 | 55,985 | 55,985 |
| M4 | 53,108 | 53,108 | 53,108 | 53,108 | 53,108 | 53,108 | 53,108 |
| M5 | 54,286 | 54,286 | 54,286 | 54,286 | 54,286 | 54,286 | 54,286 |
| N1 | 37,023 | 37,023 | 37,023 | 37,023 | 37,023 | 37,023 | 37,023 |
| N2 | 50,829 | 50,829 | 50,829 | 50,829 | 50,829 | 50,829 | 50,829 |
| N3 | 59,839 | 59,839 | 59,839 | 59,839 | 59,839 | 59,839 | 59,839 |
| N4 | 58,808 | 58,808 | 58,808 | 58,808 | 58,808 | 58,808 | 58,808 |
| N5 | 57,706 | 57,706 | 57,706 | 57,706 | 57,706 | 57,706 | 57,706 |
| O1 | 57,130 | 57,130 | 57,130 | 57,130 | 57,130 | 57,130 | 57,130 |
| O2 | 48,723 | 48,723 | 48,723 | 48,723 | 48,723 | 48,723 | 48,723 |
| O3 | 53,676 | 53,676 | 53,676 | 53,676 | 53,676 | 53,676 | 53,676 |
| O4 | 57,405 | 57,405 | 57,405 | 57,405 | 57,405 | 57,405 | 57,405 |
| O5 | 51,680 | 51,680 | 51,680 | 51,680 | 51,680 | 51,680 | 51,680 |
| P1 | 36,964 | 36,964 | 36,964 | 36,964 | 36,964 | 36,964 | 36,964 |
| P2 | 39,760 | 39,760 | 39,760 | 39,760 | 39,760 | 39,760 | 39,760 |
| P3 | 55,641 | 55,641 | 55,641 | 55,641 | 55,641 | 55,641 | 55,641 |
| P4 | 53,511 | 53,511 | 53,511 | 53,511 | 53,511 | 53,511 | 53,511 |
| P5 | 53,800 | 53,800 | 53,800 | 53,800 | 53,800 | 53,800 | 53,800 |

A, E, I, M: soil treated with R. palustris PSB-06 and dazomet (T1); B, F, J, N: soil treated with dazomet alone (T2); C, G, K, O: soil treated with R. palustris PSB-06 alone (T3); D, H, L, P: soil without treatment (blank control, T4); A-D: germination stage; E-H: seedling stage ; I-J: vigorous growth stage; M-P: harvest stage.

**Table S7. The statistics of sequences in each grade of soil fungi.**

| Sample | Kindom | Phylum | Class | Order | Family | Genus | Species |
| --- | --- | --- | --- | --- | --- | --- | --- |
| A1 | 67,185 | 64,417 | 63,437 | 60,285 | 59,077 | 56,794 | 28,116 |
| A2 | 76,704 | 73,885 | 73,700 | 69,550 | 69,475 | 68,469 | 25,948 |
| A3 | 74,645 | 72,774 | 72,697 | 65,569 | 65,551 | 65,202 | 46,052 |
| A4 | 74,968 | 72,523 | 72,405 | 63,549 | 63,608 | 59,388 | 33,095 |
| A5 | 76,457 | 75,622 | 75,499 | 75,235 | 75,276 | 75,111 | 41,529 |
| B1 | 76,620 | 73,175 | 72,760 | 71,345 | 71,248 | 70,569 | 25,882 |
| B2 | 76,238 | 71,298 | 70,667 | 68,948 | 68,785 | 63,496 | 46,705 |
| B3 | 77,252 | 75,914 | 75,782 | 72,333 | 72,340 | 71,124 | 53,395 |
| B4 | 75,622 | 72,207 | 71,930 | 66,097 | 66,033 | 64,178 | 38,222 |
| B5 | 74,468 | 71,358 | 71,013 | 64,634 | 64,056 | 54,038 | 31,219 |
| C1 | 75,266 | 72,191 | 72,120 | 71,319 | 70,790 | 69,907 | 59,513 |
| C2 | 76,071 | 75,139 | 74,960 | 74,483 | 74,650 | 73,919 | 59,451 |
| C3 | 75,452 | 73,349 | 73,206 | 61,522 | 61,604 | 60,964 | 47,212 |
| C4 | 75,468 | 69,331 | 69,015 | 64,613 | 64,564 | 64,237 | 47,768 |
| C5 | 76,365 | 73,737 | 73,665 | 73,347 | 73,319 | 72,584 | 57,220 |
| D1 | 74,548 | 73,775 | 73,452 | 70,183 | 70,268 | 69,178 | 36,197 |
| D2 | 76,844 | 62,213 | 61,921 | 61,590 | 61,333 | 43,760 | 33,571 |
| D3 | 75,320 | 69,854 | 69,529 | 68,350 | 68,157 | 67,379 | 49,177 |
| D4 | 76,001 | 66,944 | 66,575 | 66,400 | 65,739 | 58,677 | 39,984 |
| D5 | 75,266 | 57,622 | 48,554 | 48,308 | 47,373 | 38,239 | 30,378 |
| E1 | 48,459 | 40,793 | 39,349 | 37,299 | 36,168 | 34,280 | 22,140 |
| E2 | 74,151 | 52,245 | 45,283 | 41,526 | 39,651 | 37,274 | 25,127 |
| E3 | 70,992 | 64,629 | 61,582 | 57,781 | 54,314 | 52,026 | 33,861 |
| E4 | 67,990 | 48,437 | 46,412 | 44,679 | 43,025 | 38,955 | 22,699 |
| E5 | 75,160 | 57,016 | 54,661 | 52,604 | 46,214 | 45,469 | 30,493 |
| F1 | 65,357 | 55,468 | 52,427 | 48,330 | 45,329 | 42,675 | 24,031 |
| F2 | 44,225 | 37,624 | 35,213 | 33,018 | 30,957 | 30,409 | 19,436 |
| F3 | 71,949 | 61,091 | 58,803 | 44,095 | 42,472 | 41,933 | 34,782 |
| F4 | 72,459 | 62,937 | 54,111 | 50,991 | 48,310 | 47,202 | 28,785 |
| F5 | 73,785 | 66,728 | 63,171 | 58,728 | 53,675 | 51,177 | 39,035 |
| G1 | 74,738 | 58,801 | 54,752 | 51,922 | 48,457 | 46,611 | 31,510 |
| G2 | 72,725 | 46,134 | 43,365 | 40,872 | 34,693 | 33,787 | 25,890 |
| G3 | 37,841 | 31,187 | 28,275 | 25,556 | 23,384 | 22,370 | 14,109 |
| G4 | 77,040 | 33,048 | 28,753 | 18,862 | 16,309 | 15,355 | 11,050 |
| G5 | 73,177 | 64,997 | 60,843 | 59,372 | 57,909 | 55,158 | 22,033 |
| H1 | 65,833 | 59,287 | 55,560 | 55,006 | 52,081 | 49,055 | 29,354 |
| H2 | 62,668 | 57,865 | 53,726 | 52,512 | 48,435 | 45,762 | 31,162 |
| H3 | 43,772 | 33,427 | 30,929 | 27,952 | 26,655 | 24,745 | 16,662 |
| H4 | 73,779 | 68,922 | 64,983 | 34,294 | 31,948 | 30,455 | 22,105 |
| H5 | 72,194 | 69,737 | 68,909 | 33,685 | 30,147 | 29,437 | 21,793 |
| I1 | 78,175 | 37,378 | 36,387 | 35,143 | 32,942 | 32,783 | 29,691 |
| I2 | 75,457 | 56,367 | 50,374 | 41,929 | 22,401 | 21,642 | 17,569 |
| I3 | 77,652 | 42,747 | 18,578 | 18,192 | 8,866 | 7,392 | 5,103 |
| I4 | 76,796 | 46,319 | 42,952 | 40,523 | 36,703 | 35,303 | 25,245 |
| I5 | 77,874 | 24,822 | 17,963 | 17,763 | 14,225 | 14,007 | 11,647 |
| J1 | 77,120 | 31,514 | 8,567 | 7,403 | 5,196 | 4,992 | 3,862 |
| J2 | 77,721 | 36,770 | 30,595 | 30,270 | 14,246 | 14,114 | 12,319 |
| J3 | 77,128 | 53,904 | 48,909 | 31,466 | 23,315 | 22,668 | 13,546 |
| J4 | 72,107 | 31,759 | 28,618 | 25,405 | 22,813 | 22,003 | 14,151 |
| J5 | 76,480 | 32,978 | 22,868 | 20,669 | 11,051 | 10,253 | 6,856 |
| K1 | 75,461 | 47,293 | 26,760 | 24,718 | 22,791 | 21,596 | 14,944 |
| K2 | 65,106 | 54,702 | 52,411 | 48,736 | 46,121 | 43,209 | 23,224 |
| K3 | 73,654 | 50,213 | 46,590 | 43,788 | 40,819 | 39,550 | 23,651 |
| K4 | 75,879 | 31,312 | 28,930 | 26,201 | 23,700 | 23,006 | 14,768 |
| K5 | 72,881 | 54,341 | 51,908 | 50,472 | 46,386 | 45,603 | 29,562 |
| L1 | 72,569 | 61,339 | 57,528 | 56,444 | 54,515 | 51,127 | 37,663 |
| L2 | 76,994 | 59,114 | 37,532 | 36,663 | 17,712 | 16,697 | 10,463 |
| L3 | 65,049 | 57,831 | 55,980 | 53,207 | 47,792 | 45,225 | 32,431 |
| L4 | 77,456 | 25,762 | 22,800 | 22,496 | 19,632 | 19,074 | 12,475 |
| L5 | 71,396 | 67,282 | 64,104 | 61,779 | 52,638 | 50,432 | 38,024 |
| M1 | 71,469 | 61,129 | 58,910 | 42,038 | 36,206 | 34,245 | 22,390 |
| M2 | 70,379 | 62,033 | 59,027 | 55,330 | 46,728 | 44,186 | 30,393 |
| M3 | 76,334 | 67,727 | 65,486 | 62,980 | 57,335 | 54,239 | 37,335 |
| M4 | 76,614 | 62,603 | 61,039 | 52,831 | 47,231 | 44,236 | 29,468 |
| M5 | 65,910 | 57,179 | 55,870 | 49,861 | 45,802 | 43,772 | 29,046 |
| N1 | 76,559 | 68,009 | 66,345 | 61,601 | 54,979 | 51,357 | 35,403 |
| N2 | 76,956 | 66,589 | 65,013 | 61,606 | 55,925 | 52,887 | 35,408 |
| N3 | 76,912 | 68,292 | 66,368 | 63,288 | 58,302 | 55,351 | 37,069 |
| N4 | 76,882 | 68,945 | 67,291 | 53,603 | 48,755 | 46,109 | 31,990 |
| N5 | 75,572 | 66,431 | 64,566 | 59,184 | 54,286 | 51,371 | 34,805 |
| O1 | 77,152 | 66,246 | 64,406 | 61,005 | 55,435 | 52,647 | 36,742 |
| O2 | 77,381 | 67,729 | 65,832 | 60,421 | 55,570 | 52,801 | 36,380 |
| O3 | 76,356 | 66,766 | 64,903 | 61,829 | 56,428 | 53,934 | 37,308 |
| O4 | 76,421 | 67,024 | 65,506 | 61,311 | 55,812 | 52,590 | 36,351 |
| O5 | 70,614 | 61,087 | 57,060 | 54,424 | 48,127 | 45,530 | 31,214 |
| P1 | 56,796 | 49,796 | 48,420 | 46,250 | 42,439 | 39,564 | 26,689 |
| P2 | 53,975 | 40,848 | 39,849 | 38,089 | 34,754 | 32,873 | 22,940 |
| P3 | 48,210 | 42,211 | 41,206 | 39,036 | 36,109 | 34,056 | 24,217 |
| P4 | 51,140 | 44,676 | 43,511 | 41,188 | 37,716 | 34,768 | 23,984 |
| P5 | 64,645 | 54,285 | 51,822 | 49,166 | 44,779 | 41,938 | 29,498 |

A, E, I, M: soil treated with R. palustris PSB-06 and dazomet (T1); B, F, J, N: soil treated with dazomet alone (T2); C, G, K, O: soil treated with R. palustris PSB-06 alone (T3); D, H, L, P: soil without treatment (blank control, T4); A-D: germination stage; E-H: seedling stage ; I-J: vigorous growth stage; M-P: harvest stage.

**Table S8. The statistics of species in each grade of soil bacteria.**

| Sample | Kindom | Phylum | Class | Order | Family | Genus | Species |
| --- | --- | --- | --- | --- | --- | --- | --- |
| A1 | 1 | 22 | 58 | 130 | 218 | 371 | 412 |
| A2 | 1 | 22 | 59 | 130 | 220 | 373 | 411 |
| A3 | 1 | 22 | 55 | 127 | 213 | 368 | 408 |
| A4 | 1 | 21 | 56 | 128 | 220 | 376 | 416 |
| A5 | 1 | 22 | 60 | 134 | 224 | 376 | 417 |
| B1 | 1 | 22 | 57 | 126 | 215 | 372 | 416 |
| B2 | 1 | 20 | 56 | 127 | 211 | 348 | 385 |
| B3 | 1 | 22 | 58 | 130 | 220 | 375 | 420 |
| B4 | 1 | 22 | 59 | 129 | 221 | 368 | 408 |
| B5 | 1 | 21 | 55 | 129 | 221 | 365 | 405 |
| C1 | 1 | 22 | 56 | 128 | 220 | 361 | 400 |
| C2 | 1 | 21 | 57 | 133 | 228 | 382 | 424 |
| C3 | 1 | 21 | 54 | 124 | 206 | 345 | 381 |
| C4 | 1 | 22 | 61 | 133 | 229 | 386 | 429 |
| C5 | 1 | 22 | 59 | 130 | 224 | 385 | 426 |
| D1 | 1 | 22 | 59 | 130 | 213 | 364 | 405 |
| D2 | 1 | 22 | 61 | 135 | 220 | 379 | 420 |
| D3 | 1 | 23 | 61 | 138 | 230 | 383 | 427 |
| D4 | 1 | 19 | 55 | 129 | 221 | 372 | 415 |
| D5 | 1 | 22 | 59 | 134 | 222 | 377 | 417 |
| E1 | 1 | 22 | 56 | 131 | 233 | 399 | 435 |
| E2 | 1 | 22 | 56 | 137 | 237 | 409 | 446 |
| E3 | 1 | 23 | 54 | 134 | 235 | 414 | 450 |
| E4 | 1 | 23 | 57 | 136 | 231 | 407 | 439 |
| E5 | 1 | 23 | 58 | 140 | 241 | 414 | 455 |
| F1 | 1 | 22 | 59 | 139 | 241 | 417 | 458 |
| F2 | 1 | 24 | 60 | 141 | 236 | 413 | 449 |
| F3 | 1 | 24 | 59 | 140 | 242 | 414 | 451 |
| F4 | 1 | 21 | 55 | 137 | 236 | 398 | 441 |
| F5 | 1 | 23 | 60 | 138 | 235 | 398 | 433 |
| G1 | 1 | 23 | 59 | 141 | 240 | 418 | 455 |
| G2 | 1 | 23 | 59 | 141 | 239 | 425 | 462 |
| G3 | 1 | 21 | 57 | 138 | 230 | 392 | 428 |
| G4 | 1 | 23 | 59 | 138 | 239 | 413 | 450 |
| G5 | 1 | 22 | 61 | 142 | 240 | 405 | 437 |
| H1 | 1 | 22 | 59 | 139 | 237 | 413 | 451 |
| H2 | 1 | 21 | 58 | 138 | 240 | 409 | 445 |
| H3 | 1 | 21 | 57 | 138 | 238 | 400 | 435 |
| H4 | 1 | 21 | 55 | 135 | 235 | 398 | 433 |
| H5 | 1 | 24 | 60 | 143 | 244 | 420 | 455 |
| I1 | 1 | 23 | 56 | 139 | 243 | 435 | 476 |
| I2 | 1 | 23 | 58 | 139 | 245 | 436 | 478 |
| I3 | 1 | 24 | 56 | 138 | 247 | 433 | 470 |
| I4 | 1 | 23 | 63 | 145 | 243 | 429 | 465 |
| I5 | 1 | 23 | 55 | 138 | 239 | 428 | 471 |
| J1 | 1 | 23 | 57 | 140 | 240 | 421 | 457 |
| J2 | 1 | 23 | 58 | 140 | 242 | 419 | 459 |
| J3 | 1 | 23 | 55 | 135 | 235 | 407 | 443 |
| J4 | 1 | 23 | 58 | 139 | 247 | 430 | 468 |
| J5 | 1 | 22 | 57 | 139 | 243 | 433 | 474 |
| K1 | 1 | 24 | 61 | 144 | 245 | 418 | 459 |
| K2 | 1 | 22 | 58 | 142 | 244 | 416 | 449 |
| K3 | 1 | 23 | 59 | 137 | 241 | 415 | 446 |
| K4 | 1 | 22 | 56 | 135 | 232 | 415 | 456 |
| K5 | 1 | 22 | 58 | 142 | 244 | 434 | 474 |
| L1 | 1 | 20 | 55 | 136 | 234 | 395 | 431 |
| L2 | 1 | 23 | 59 | 143 | 236 | 412 | 449 |
| L3 | 1 | 22 | 57 | 141 | 243 | 427 | 472 |
| L4 | 1 | 22 | 59 | 143 | 246 | 434 | 477 |
| L5 | 1 | 22 | 59 | 141 | 238 | 416 | 452 |
| M1 | 1 | 21 | 50 | 126 | 221 | 387 | 417 |
| M2 | 1 | 21 | 54 | 133 | 232 | 410 | 445 |
| M3 | 1 | 22 | 54 | 131 | 233 | 410 | 445 |
| M4 | 1 | 22 | 56 | 138 | 239 | 427 | 468 |
| M5 | 1 | 22 | 55 | 134 | 238 | 423 | 464 |
| N1 | 1 | 21 | 56 | 136 | 235 | 426 | 461 |
| N2 | 1 | 23 | 59 | 140 | 245 | 433 | 474 |
| N3 | 1 | 21 | 54 | 129 | 217 | 374 | 405 |
| N4 | 1 | 21 | 55 | 138 | 238 | 420 | 458 |
| N5 | 1 | 22 | 60 | 138 | 245 | 422 | 461 |
| O1 | 1 | 22 | 61 | 144 | 253 | 443 | 482 |
| O2 | 1 | 21 | 57 | 135 | 230 | 390 | 425 |
| O3 | 1 | 23 | 56 | 141 | 243 | 434 | 472 |
| O4 | 1 | 22 | 59 | 140 | 240 | 423 | 458 |
| O5 | 1 | 22 | 58 | 141 | 241 | 423 | 467 |
| P1 | 1 | 20 | 57 | 135 | 224 | 374 | 402 |
| P2 | 1 | 22 | 57 | 138 | 241 | 427 | 468 |
| P3 | 1 | 20 | 56 | 133 | 235 | 405 | 442 |
| P4 | 1 | 21 | 56 | 137 | 234 | 410 | 446 |
| P5 | 1 | 21 | 56 | 136 | 236 | 416 | 457 |
| Total | 1 | 24 | 66 | 151 | 269 | 486 | 541 |

A, E, I, M: soil treated with R. palustris PSB-06 and dazomet (T1); B, F, J, N: soil treated with dazomet alone (T2); C, G, K, O: soil treated with R. palustris PSB-06 alone (T3); D, H, L, P: soil without treatment (blank control, T4); A-D: germination stage; E-H: seedling stage ; I-J: vigorous growth stage; M-P: harvest stage.

**Table S9. The statistics of species in each grade of soil fungi.**

| Sample | Kindom | Phylum | Class | Order | Family | Genus | Species |
| --- | --- | --- | --- | --- | --- | --- | --- |
| A1 | 1 | 8 | 22 | 54 | 105 | 167 | 185 |
| A2 | 1 | 6 | 16 | 39 | 66 | 100 | 101 |
| A3 | 1 | 7 | 21 | 42 | 69 | 104 | 112 |
| A4 | 1 | 7 | 17 | 40 | 72 | 123 | 126 |
| A5 | 1 | 6 | 15 | 35 | 57 | 89 | 92 |
| B1 | 1 | 6 | 16 | 34 | 65 | 109 | 101 |
| B2 | 1 | 6 | 17 | 37 | 65 | 98 | 91 |
| B3 | 1 | 6 | 16 | 35 | 63 | 98 | 103 |
| B4 | 1 | 6 | 15 | 32 | 52 | 90 | 91 |
| B5 | 1 | 6 | 17 | 38 | 71 | 118 | 117 |
| C1 | 1 | 7 | 16 | 37 | 60 | 89 | 100 |
| C2 | 1 | 7 | 17 | 35 | 63 | 95 | 100 |
| C3 | 1 | 6 | 15 | 34 | 64 | 95 | 104 |
| C4 | 1 | 6 | 16 | 35 | 61 | 100 | 103 |
| C5 | 1 | 8 | 20 | 37 | 65 | 101 | 105 |
| D1 | 1 | 7 | 20 | 42 | 70 | 99 | 108 |
| D2 | 1 | 7 | 17 | 36 | 60 | 97 | 103 |
| D3 | 1 | 7 | 16 | 36 | 62 | 96 | 103 |
| D4 | 1 | 6 | 16 | 33 | 60 | 93 | 104 |
| D5 | 1 | 7 | 18 | 40 | 67 | 107 | 119 |
| E1 | 1 | 8 | 22 | 56 | 106 | 182 | 206 |
| E2 | 1 | 7 | 17 | 39 | 72 | 107 | 107 |
| E3 | 1 | 8 | 19 | 47 | 80 | 133 | 138 |
| E4 | 1 | 8 | 20 | 48 | 89 | 143 | 149 |
| E5 | 1 | 8 | 19 | 49 | 84 | 138 | 152 |
| F1 | 1 | 8 | 20 | 52 | 92 | 151 | 169 |
| F2 | 1 | 8 | 19 | 46 | 80 | 135 | 131 |
| F3 | 1 | 8 | 18 | 50 | 90 | 140 | 143 |
| F4 | 1 | 7 | 17 | 36 | 68 | 100 | 111 |
| F5 | 1 | 8 | 19 | 46 | 83 | 135 | 130 |
| G1 | 1 | 7 | 15 | 37 | 63 | 95 | 88 |
| G2 | 1 | 6 | 15 | 42 | 58 | 89 | 98 |
| G3 | 1 | 7 | 18 | 45 | 73 | 108 | 120 |
| G4 | 1 | 7 | 17 | 43 | 75 | 102 | 111 |
| G5 | 1 | 8 | 20 | 43 | 74 | 118 | 111 |
| H1 | 1 | 8 | 24 | 55 | 102 | 179 | 201 |
| H2 | 1 | 8 | 23 | 55 | 98 | 165 | 192 |
| H3 | 1 | 7 | 16 | 45 | 64 | 100 | 119 |
| H4 | 1 | 7 | 18 | 43 | 71 | 107 | 116 |
| H5 | 1 | 8 | 20 | 51 | 91 | 151 | 163 |
| I1 | 1 | 6 | 16 | 43 | 61 | 100 | 109 |
| I2 | 1 | 6 | 15 | 42 | 75 | 116 | 130 |
| I3 | 1 | 6 | 17 | 42 | 75 | 113 | 115 |
| I4 | 1 | 8 | 20 | 47 | 83 | 117 | 127 |
| I5 | 1 | 7 | 16 | 42 | 70 | 123 | 126 |
| J1 | 1 | 7 | 19 | 40 | 68 | 108 | 107 |
| J2 | 1 | 8 | 16 | 42 | 70 | 106 | 107 |
| J3 | 1 | 7 | 18 | 44 | 72 | 112 | 124 |
| J4 | 1 | 8 | 18 | 44 | 84 | 135 | 148 |
| J5 | 1 | 7 | 16 | 38 | 69 | 104 | 120 |
| K1 | 1 | 8 | 21 | 44 | 82 | 130 | 140 |
| K2 | 1 | 7 | 20 | 49 | 86 | 127 | 135 |
| K3 | 1 | 7 | 19 | 44 | 71 | 116 | 129 |
| K4 | 1 | 7 | 17 | 49 | 77 | 118 | 128 |
| K5 | 1 | 8 | 17 | 43 | 76 | 118 | 121 |
| L1 | 1 | 7 | 19 | 44 | 81 | 124 | 142 |
| L2 | 1 | 8 | 17 | 46 | 72 | 111 | 125 |
| L3 | 1 | 8 | 24 | 60 | 117 | 204 | 245 |
| L4 | 1 | 8 | 18 | 48 | 83 | 134 | 157 |
| L5 | 1 | 8 | 21 | 50 | 88 | 153 | 184 |
| M1 | 1 | 8 | 22 | 51 | 84 | 130 | 145 |
| M2 | 1 | 8 | 22 | 53 | 99 | 163 | 183 |
| M3 | 1 | 8 | 25 | 57 | 103 | 186 | 212 |
| M4 | 1 | 8 | 23 | 54 | 106 | 180 | 202 |
| M5 | 1 | 8 | 24 | 57 | 108 | 178 | 204 |
| N1 | 1 | 8 | 21 | 50 | 98 | 168 | 192 |
| N2 | 1 | 8 | 24 | 57 | 104 | 173 | 197 |
| N3 | 1 | 8 | 24 | 55 | 104 | 184 | 200 |
| N4 | 1 | 8 | 23 | 52 | 101 | 170 | 191 |
| N5 | 1 | 8 | 22 | 53 | 98 | 171 | 195 |
| O1 | 1 | 8 | 23 | 57 | 105 | 171 | 196 |
| O2 | 1 | 8 | 23 | 53 | 99 | 171 | 198 |
| O3 | 1 | 8 | 25 | 54 | 103 | 182 | 200 |
| O4 | 1 | 8 | 23 | 56 | 104 | 192 | 218 |
| O5 | 1 | 8 | 25 | 55 | 103 | 174 | 196 |
| P1 | 1 | 8 | 24 | 56 | 106 | 179 | 203 |
| P2 | 1 | 8 | 25 | 57 | 101 | 173 | 200 |
| P3 | 1 | 8 | 21 | 52 | 99 | 169 | 201 |
| P4 | 1 | 8 | 23 | 58 | 104 | 179 | 204 |
| P5 | 1 | 8 | 22 | 53 | 99 | 172 | 195 |
| Total | 1 | 8 | 27 | 67 | 144 | 274 | 332 |

A, E, I, M: soil treated with R. palustris PSB-06 and dazomet (T1); B, F, J, N: soil treated with dazomet alone (T2); C, G, K, O: soil treated with R. palustris PSB-06 alone (T3); D, H, L, P: soil without treatment (blank control, T4); A-D: germination stage; E-H: seedling stage ; I-J: vigorous growth stage; M-P: harvest stage.

**Table S10. Taxonomic structure of the soil bacterial diversity at the phylum level.**

| Germination stage | | | | | | | | | | | | | | | Seedling stage | | | | | | | | | | | | | | | |
| --- | --- | --- | --- | --- | --- | --- | --- | --- | --- | --- | --- | --- | --- | --- | --- | --- | --- | --- | --- | --- | --- | --- | --- | --- | --- | --- | --- | --- | --- | --- |
| Phylum | A | Phylum | | | B | | Phylum | | C | | Phylum | | D | | Phylum | | E | | Phylum | | F | | Phylum | | G | | Phylum | | H | |
| Proteobacteria | 0.38 | Proteobacteria | | | 0.37 | | Proteobacteria | | 0.36 | | Proteobacteria | | 0.36 | | Proteobacteria | | 0.41 | | Proteobacteria | | 0.41 | | Proteobacteria | | 0.40 | | Acidobacteria | | 0.39 | |
| Bacteroidetes | 0.17 | Bacteroidetes | | | 0.20 | | Bacteroidetes | | 0.18 | | Bacteroidetes | | 0.14 | | Acidobacteria | | 0.24 | | Acidobacteria | | 0.24 | | Acidobacteria | | 0.26 | | Actinobacteria | | 0.29 | |
| Actinobacteria | 0.15 | Actinobacteria | | | 0.14 | | Actinobacteria | | 0.14 | | Actinobacteria | | 0.13 | | Gemmatimonadetes | | 0.13 | | Gemmatimonadetes | | 0.15 | | Gemmatimonadetes | | 0.14 | | Armatimonadetes | | 0.12 | |
| Gemmatimonadetes | 0.07 | Patescibacteria | | | 0.07 | | Gemmatimonadetes | | 0.09 | | Acidobacteria | | 0.09 | | Actinobacteria | | 0.09 | | Actinobacteria | | 0.09 | | Actinobacteria | | 0.09 | | Bacteroidetes | | 0.09 | |
| Firmicutes | 0.05 | Gemmatimonadetes | | | 0.06 | | Chloroflexi | | 0.05 | | Gemmatimonadetes | | 0.09 | | Firmicutes | | 0.04 | | Firmicutes | | 0.04 | | Firmicutes | | 0.04 | | Chloroflexi | | 0.03 | |
| Patescibacteria | 0.05 | Firmicutes | | | 0.06 | | Firmicutes | | 0.05 | | Chloroflexi | | 0.07 | | Patescibacteria | | 0.03 | | Patescibacteria | | 0.03 | | Patescibacteria | | 0.02 | | Cyanobacteria | | 0.02 | |
| Acidobacteria | 0.04 | Acidobacteria | | | 0.03 | | Patescibacteria | | 0.05 | | Firmicutes | | 0.04 | | Cyanobacteria | | 0.02 | | Chloroflexi | | 0.02 | | Chloroflexi | | 0.02 | | Dependentiae | | 0.02 | |
| Chloroflexi | 0.03 | Planctomycetes | | | 0.03 | | Acidobacteria | | 0.04 | | Planctomycetes | | 0.04 | | Chloroflexi | | 0.01 | | Cyanobacteria | | 0.01 | | Cyanobacteria | | 0.01 | | Elusimicrobia | | 0.01 | |
| Planctomycetes | 0.03 | Chloroflexi | | | 0.03 | | Planctomycetes | | 0.04 | | Patescibacteria | | 0.03 | | Bacteroidetes | | 0.01 | | Bacteroidetes | | 0.01 | | Bacteroidetes | | 0.01 | | Epsilonbacteraeota | | 0.01 | |
| other | 0.01 | other | | | 0.01 | | other | | 0.01 | | other | | 0.02 | | other | | 0.01 | | other | | 0.01 | | other | | 0.01 | | other | | 0.02 | |
| Vigorous growth stage | | | | | | | | | | | | | | | | Harvest stage | | | | | | | | | | | | | | |
| Phylum | | | I | Phylum | | J | | Phylum | | K | | Phylum | | L | | Phylum | | M | | Phylum | | N | | Phylum | | O | | Phylum | | P |
| Proteobacteria | | | 0.47 | Proteobacteria | | 0.40 | | Proteobacteria | | 0.38 | | Proteobacteria | | 0.44 | | Proteobacteria | | 0.49 | | Proteobacteria | | 0.42 | | Proteobacteria | | 0.47 | | Proteobacteria | | 0.41 |
| Acidobacteria | | | 0.18 | Acidobacteria | | 0.23 | | Acidobacteria | | 0.27 | | Acidobacteria | | 0.24 | | Acidobacteria | | 0.18 | | Acidobacteria | | 0.24 | | Acidobacteria | | 0.23 | | Acidobacteria | | 0.26 |
| Gemmatimonadetes | | | 0.11 | Gemmatimonadetes | | 0.13 | | Gemmatimonadetes | | 0.14 | | Gemmatimonadetes | | 0.10 | | Gemmatimonadetes | | 0.09 | | Gemmatimonadetes | | 0.11 | | Gemmatimonadetes | | 0.12 | | Gemmatimonadetes | | 0.10 |
| Actinobacteria | | | 0.10 | Actinobacteria | | 0.09 | | Actinobacteria | | 0.08 | | Actinobacteria | | 0.08 | | Actinobacteria | | 0.09 | | Actinobacteria | | 0.10 | | Actinobacteria | | 0.08 | | Actinobacteria | | 0.10 |
| Cyanobacteria | | | 0.05 | Cyanobacteria | | 0.05 | | Firmicutes | | 0.06 | | Cyanobacteria | | 0.04 | | Firmicutes | | 0.07 | | Firmicutes | | 0.07 | | Firmicutes | | 0.03 | | Rokubacteria | | 0.05 |
| Firmicutes | | | 0.04 | Firmicutes | | 0.03 | | Patescibacteria | | 0.02 | | Firmicutes | | 0.03 | | Patescibacteria | | 0.02 | | Patescibacteria | | 0.02 | | Patescibacteria | | 0.02 | | Firmicutes | | 0.02 |
| Patescibacteria | | | 0.02 | Patescibacteria | | 0.03 | | Chloroflexi | | 0.02 | | Rokubacteria | | 0.02 | | Cyanobacteria | | 0.02 | | Bacteroidetes | | 0.01 | | Chloroflexi | | 0.02 | | Cyanobacteria | | 0.02 |
| Chloroflexi | | | 0.01 | Chloroflexi | | 0.02 | | Cyanobacteria | | 0.01 | | Chloroflexi | | 0.01 | | Bacteroidetes | | 0.02 | | Cyanobacteria | | 0.01 | | Cyanobacteria | | 0.01 | | Chloroflexi | | 0.01 |
| Bacteroidetes | | | 0.01 | Bacteroidetes | | 0.01 | | Bacteroidetes | | 0.01 | | Patescibacteria | | 0.01 | | Chloroflexi | | 0.01 | | Chloroflexi | | 0.01 | | Rokubacteria | | 0.01 | | Bacteroidetes | | 0.01 |
| other | | | 0.01 | other | | 0.01 | | other | | 0.01 | | other | | 0.02 | | other | | 0.01 | | other | | 0.01 | | other | | 0.01 | | other | | 0.01 |

A, E, I, M: soil treated with R. palustris PSB-06 and dazomet (T1); B, F, J, N: soil treated with dazomet alone (T2); C, G, K, O: soil treated with R. palustris PSB-06 alone (T3); D, H, L, P: soil without treatment (blank control, T4).

**Table S11. Taxonomic structure of the soil fungal diversity at the phylum level.**

| Germination stage | | | | | | | | Seedling stage | | | | | | | | | | | | |  |
| --- | --- | --- | --- | --- | --- | --- | --- | --- | --- | --- | --- | --- | --- | --- | --- | --- | --- | --- | --- | --- | --- |
| Phylum | A | Phylum | B | Phylum | C | Phylum | D | Phylum | E | Phylum | F | | Phylum | | G | | Phylum | | H | |  |
| Ascomycota | 0.83 | Ascomycota | 0.83 | Ascomycota | 0.83 | Ascomycota | 0.66 | Ascomycota | 0.44 | Ascomycota | 0.47 | | Ascomycota | | 0.39 | | Ascomycota | | 0.47 | |  |
| Basidiomycota | 0.12 | Basidiomycota | 0.11 | Basidiomycota | 0.11 | Basidiomycota | 0.13 | Basidiomycota | 0.30 | Basidiomycota | 0.36 | | Unclassified | | 0.29 | | Basidiomycota | | 0.39 | |  |
| Unclassified | 0.03 | Unclassified | 0.04 | Unclassified | 0.04 | Unclassified | 0.13 | Unclassified | 0.21 | Unclassified | 0.14 | | Basidiomycota | | 0.28 | | Unclassified | | 0.10 | |  |
| Mortierellomycota | 0.01 | Mortierellomycota | 0.01 | Mortierellomycota | 0.01 | Mortierellomycota | 0.05 | Mortierellomycota | 0.02 | Rozellomycota | 0.01 | | Mortierellomycota | | 0.02 | | Mortierellomycota | | 0.02 | |  |
| Chytridiomycota | 0.00 | Chytridiomycota | 0.01 | Chytridiomycota | 0.01 | Chytridiomycota | 0.03 | Olpidiomycota | 0.01 | Mucoromycota | 0.01 | | Mucoromycota | | 0.01 | | Mucoromycota | | 0.01 | |  |
| other | 0.00 | other | 0.00 | other | 0.00 | other | 0.00 | other | 0.02 | other | 0.02 | | other | | 0.01 | | other | | 0.01 | |  |
| Vigorous growth stage | | | | | | | | Harvest stage | | | | | | | | | | | | | |
| Phylum | I | Phylum | J | Phylum | K | Phylum | L | Phylum | M | Phylum | | N | | Phylum | | O | | Phylum | | P | |
| Unclassified | 0.46 | Unclassified | 0.51 | Ascomycota | 0.40 | Ascomycota | 0.46 | Ascomycota | 0.54 | Ascomycota | | 0.60 | | Ascomycota | | 0.60 | | Ascomycota | | 0.58 | |
| Ascomycota | 0.32 | Ascomycota | 0.25 | Unclassified | 0.34 | Unclassified | 0.24 | Basidiomycota | 0.28 | Basidiomycota | | 0.25 | | Basidiomycota | | 0.23 | | Basidiomycota | | 0.20 | |
| Chytridiomycota | 0.07 | Basidiomycota | 0.10 | Basidiomycota | 0.15 | Basidiomycota | 0.16 | Unclassified | 0.14 | Unclassified | | 0.12 | | Unclassified | | 0.13 | | Unclassified | | 0.16 | |
| Basidiomycota | 0.07 | Chytridiomycota | 0.09 | Chytridiomycota | 0.05 | Mortierellomycota | 0.06 | Mortierellomycota | 0.02 | Mortierellomycota | | 0.02 | | Mortierellomycota | | 0.02 | | Mortierellomycota | | 0.04 | |
| Mortierellomycota | 0.05 | Mortierellomycota | 0.02 | Mortierellomycota | 0.04 | Chytridiomycota | 0.06 | Mucoromycota | 0.01 | Mucoromycota | | 0.01 | | Mucoromycota | | 0.01 | | Mucoromycota | | 0.01 | |
| other | 0.03 | other | 0.02 | other | 0.02 | other | 0.02 | other | 0.01 | other | | 0.01 | | other | | 0.01 | | other | | 0.01 | |

A, E, I, M: soil treated with R. palustris PSB-06 and dazomet (T1); B, F, J, N: soil treated with dazomet alone (T2); C, G, K, O: soil treated with R. palustris PSB-06 alone (T3); D, H, L, P: soil without treatment (blank control, T4).

**Table S12. Taxonomic structure of the soil bacterial diversity at the family level.**

| Family | Germination stage | | | | Seedling stage | | | | Vigorous growth stage | | | | Harvest stage | | | |
| --- | --- | --- | --- | --- | --- | --- | --- | --- | --- | --- | --- | --- | --- | --- | --- | --- |
|  | A | B | C | D | E | F | G | H | I | J | K | L | M | N | O | P |
| Acidobacteriaceae_Subgroup_1 | 0.012 | 0.010 | 0.006 | 0.006 | 0.021 | 0.024 | 0.014 | 0.015 | 0.019 | 0.022 | 0.018 | 0.010 | 0.047 | 0.056 | 0.021 | 0.006 |
| Bacillaceae | 0.019 | 0.018 | 0.016 | 0.012 | 0.009 | 0.008 | 0.006 | 0.008 | 0.012 | 0.009 | 0.010 | 0.011 | 0.011 | 0.009 | 0.007 | 0.005 |
| Burkholderiaceae | 0.148 | 0.167 | 0.133 | 0.101 | 0.021 | 0.021 | 0.013 | 0.015 | 0.050 | 0.036 | 0.035 | 0.057 | 0.093 | 0.069 | 0.053 | 0.025 |
| Caulobacteraceae | 0.014 | 0.014 | 0.012 | 0.013 | 0.011 | 0.010 | 0.009 | 0.007 | 0.007 | 0.009 | 0.008 | 0.007 | 0.009 | 0.009 | 0.009 | 0.006 |
| Devosiaceae | 0.006 | 0.006 | 0.007 | 0.005 | 0.010 | 0.010 | 0.008 | 0.005 | 0.009 | 0.010 | 0.010 | 0.008 | 0.010 | 0.012 | 0.010 | 0.005 |
| Gemmatimonadaceae | 0.062 | 0.053 | 0.075 | 0.082 | 0.125 | 0.137 | 0.134 | 0.116 | 0.104 | 0.126 | 0.135 | 0.100 | 0.084 | 0.108 | 0.117 | 0.098 |
| Rhizobiaceae | 0.004 | 0.003 | 0.004 | 0.005 | 0.010 | 0.008 | 0.007 | 0.005 | 0.011 | 0.011 | 0.008 | 0.011 | 0.011 | 0.010 | 0.010 | 0.008 |
| Rhodanobacteraceae | 0.048 | 0.038 | 0.022 | 0.013 | 0.033 | 0.032 | 0.024 | 0.019 | 0.088 | 0.033 | 0.017 | 0.021 | 0.066 | 0.041 | 0.043 | 0.015 |
| Solibacteraceae_Subgroup_3 | 0.009 | 0.009 | 0.010 | 0.020 | 0.024 | 0.022 | 0.020 | 0.025 | 0.023 | 0.026 | 0.026 | 0.027 | 0.021 | 0.028 | 0.026 | 0.027 |
| Sphingomonadaceae | 0.073 | 0.055 | 0.082 | 0.085 | 0.068 | 0.078 | 0.070 | 0.062 | 0.080 | 0.070 | 0.050 | 0.058 | 0.062 | 0.048 | 0.070 | 0.076 |
| uncultured_bacterium_c_Subgroup_6 | 0.006 | 0.004 | 0.009 | 0.029 | 0.081 | 0.082 | 0.095 | 0.112 | 0.050 | 0.059 | 0.068 | 0.088 | 0.033 | 0.035 | 0.077 | 0.116 |
| uncultured_bacterium_o_Acidobacteriales | 0.006 | 0.005 | 0.007 | 0.015 | 0.074 | 0.079 | 0.083 | 0.086 | 0.058 | 0.088 | 0.107 | 0.069 | 0.041 | 0.064 | 0.064 | 0.047 |
| uncultured_bacterium_o_Elsterales | 0.003 | 0.003 | 0.003 | 0.004 | 0.017 | 0.019 | 0.016 | 0.017 | 0.011 | 0.015 | 0.016 | 0.013 | 0.014 | 0.017 | 0.010 | 0.008 |
| uncultured_bacterium_o_Gaiellales | 0.015 | 0.009 | 0.014 | 0.014 | 0.029 | 0.030 | 0.030 | 0.028 | 0.023 | 0.032 | 0.026 | 0.019 | 0.019 | 0.020 | 0.019 | 0.011 |
| uncultured_bacterium_o_Saccharimonadales | 0.047 | 0.060 | 0.036 | 0.028 | 0.030 | 0.030 | 0.022 | 0.017 | 0.021 | 0.031 | 0.024 | 0.012 | 0.022 | 0.021 | 0.021 | 0.006 |
| Xanthobacteraceae | 0.020 | 0.021 | 0.020 | 0.025 | 0.033 | 0.031 | 0.032 | 0.032 | 0.031 | 0.036 | 0.034 | 0.036 | 0.038 | 0.042 | 0.042 | 0.033 |
| other | 0.647 | 0.682 | 0.736 | 0.747 | 0.665 | 0.656 | 0.672 | 0.658 | 0.710 | 0.653 | 0.644 | 0.669 | 0.661 | 0.645 | 0.665 | 0.732 |

A, E, I, M: soil treated with R. palustris PSB-06 and dazomet (T1); B, F, J, N: soil treated with dazomet alone (T2); C, G, K, O: soil treated with R. palustris PSB-06 alone (T3); D, H, L, P: soil without treatment (blank control, T4).

**Table S13. Taxonomic structure of the soil fungal diversity at the family level.**

| Family | Germination stage | | | | Seedling stage | | | | Vigorous growth stage | | | | Harvest stage | | | |
| --- | --- | --- | --- | --- | --- | --- | --- | --- | --- | --- | --- | --- | --- | --- | --- | --- |
|  | A | B | C | D | E | F | G | H | I | J | K | L | M | N | O | P |
| Aspergillaceae | 0.034 | 0.037 | 0.053 | 0.042 | 0.051 | 0.052 | 0.038 | 0.101 | 0.010 | 0.015 | 0.036 | 0.041 | 0.064 | 0.078 | 0.087 | 0.072 |
| Chaetomiaceae | 0.443 | 0.424 | 0.515 | 0.243 | 0.066 | 0.066 | 0.063 | 0.056 | 0.018 | 0.016 | 0.051 | 0.050 | 0.056 | 0.051 | 0.053 | 0.060 |
| Cladosporiaceae | 0.014 | 0.022 | 0.016 | 0.015 | 0.008 | 0.017 | 0.005 | 0.021 | 0.002 | 0.003 | 0.010 | 0.009 | 0.017 | 0.014 | 0.015 | 0.013 |
| Didymellaceae | 0.004 | 0.004 | 0.004 | 0.007 | 0.020 | 0.010 | 0.003 | 0.007 | 0.001 | 0.002 | 0.004 | 0.005 | 0.011 | 0.016 | 0.017 | 0.015 |
| Hypocreaceae | 0.020 | 0.025 | 0.014 | 0.019 | 0.016 | 0.021 | 0.015 | 0.020 | 0.010 | 0.016 | 0.038 | 0.021 | 0.037 | 0.040 | 0.020 | 0.019 |
| Microascaceae | 0.029 | 0.040 | 0.035 | 0.034 | 0.021 | 0.021 | 0.011 | 0.012 | 0.004 | 0.005 | 0.010 | 0.010 | 0.013 | 0.009 | 0.010 | 0.012 |
| Mortierellaceae | 0.013 | 0.011 | 0.014 | 0.051 | 0.019 | 0.014 | 0.019 | 0.025 | 0.052 | 0.022 | 0.040 | 0.058 | 0.021 | 0.018 | 0.022 | 0.038 |
| Nectriaceae | 0.141 | 0.104 | 0.046 | 0.061 | 0.044 | 0.031 | 0.021 | 0.035 | 0.013 | 0.012 | 0.074 | 0.033 | 0.048 | 0.046 | 0.046 | 0.046 |
| Plectosphaerellaceae | 0.003 | 0.001 | 0.005 | 0.012 | 0.009 | 0.010 | 0.004 | 0.010 | 0.004 | 0.002 | 0.008 | 0.008 | 0.014 | 0.022 | 0.023 | 0.022 |
| Pleosporaceae | 0.027 | 0.030 | 0.025 | 0.040 | 0.014 | 0.012 | 0.013 | 0.011 | 0.002 | 0.002 | 0.007 | 0.008 | 0.016 | 0.015 | 0.016 | 0.013 |
| Unclassified | 0.101 | 0.100 | 0.089 | 0.172 | 0.341 | 0.324 | 0.451 | 0.395 | 0.701 | 0.797 | 0.497 | 0.459 | 0.353 | 0.289 | 0.282 | 0.286 |
| other | 0.170 | 0.203 | 0.184 | 0.304 | 0.390 | 0.421 | 0.357 | 0.306 | 0.183 | 0.107 | 0.224 | 0.298 | 0.350 | 0.401 | 0.409 | 0.402 |

A, E, I, M: soil treated with R. palustris PSB-06 and dazomet (T1); B, F, J, N: soil treated with dazomet alone (T2); C, G, K, O: soil treated with R. palustris PSB-06 alone (T3); D, H, L, P: soil without treatment (blank control, T4).

**Table S14. Significant difference analysis (p<0.05) of the top 16 common bacterial families from at different developmental stages**.

| Family | Germination stage | | | | Seedling stage | | | | Vigorous growth stage | | | | Harvest stage | | | |
| --- | --- | --- | --- | --- | --- | --- | --- | --- | --- | --- | --- | --- | --- | --- | --- | --- |
|  | T1 | T2 | T3 | T4 | T1 | T2 | T3 | T4 | T1 | T2 | T3 | T4 | T1 | T2 | T3 | T4 |
| Acidobacteriaceae | - | - | - | - | 0.02a | 0.02a | 0.01a | 0.02a | 0.02ab | 0.02a | 0.02ab | 0.01b | - | - | - | - |
| Acidobacteriales | - | - | - | - | 0.07a | 0.08a | 0.08a | 0.09a | 0.06b | 0.09ab | 0.11a | 0.07b | 0.04a | 0.06a | 0.06a | 0.05a |
| Alphaproteobacteria | - | - | - | - | 0.01a | 0.01a | 0.01a | 0.01a | - | - | - | - | - | - | - | - |
| Bacillaceae | 0.02a | 0.02a | 0.02ab | 0.01b | - | - | - | - | 0.01a | 0.01a | 0.01a | 0.01a | - | - | - | - |
| Burkholderiaceae | 0.15ab | 0.17a | 0.13ab | 0.10b | 0.02a | 0.02ab | 0.01b | 0.01ab | 0.05a | 0.04a | 0.03a | 0.06a | 0.09a | 0.07ab | 0.05ab | 0.03b |
| Caulobacteraceae | 0.01a | 0.01a | 0.01a | 0.01a | - | - | - | - | - | - | - | - | - | - | - | - |
| Chitinophagaceae | 0.02ab | 0.02b | 0.03a | 0.02ab | - | - | - | - | - | - | - | - | - | - | - | - |
| Elsterales | - | - | - | - | 0.02a | 0.02a | 0.02a | 0.02a | 0.01a | 0.01a | 0.02a | 0.01a | 0.01a | 0.02a | 0.01a | 0.01a |
| Gaiellales | 0.02a | 0.01a | 0.01a | 0.01a | 0.03a | 0.03a | 0.03a | 0.03a | 0.02b | 0.03a | 0.03ab | 0.02c | 0.02ab | 0.02a | 0.02ab | 0.01b |
| Gammaproteobacteria_Incertae_Sedis | - | - | - | - | - | - | - | - | - | - | - | - | 0.01a | 0.01a | 0.01a | 0.01a |
| Gemmatimonadaceae | 0.06a | 0.05a | 0.07a | 0.08a | 0.13a | 0.14a | 0.13a | 0.12a | 0.10bc | 0.13ab | 0.13a | 0.10c | 0.08b | 0.11ab | 0.12a | 0.10ab |
| Haliangiaceae | - | - | - | - | 0.02a | 0.02a | 0.02a | 0.02a | 0.01a | 0.01a | 0.02a | 0.02a | 0.01b | 0.01b | 0.02a | 0.02ab |
| Isosphaeraceae | 0.03a | 0.02a | 0.03a | 0.03a | - | - | - | - | - | - | - | - | - | - | - | - |
| Micrococcaceae | 0.06a | 0.06a | 0.05a | 0.03b | - | - | - | - | - | - | - | - | - | - | - | - |
| Nitrosomonadaceae | - | - | - | - | 0.04a | 0.05a | 0.05a | 0.04a | 0.03a | 0.03a | 0.04a | 0.03a | 0.02b | 0.01b | 0.03a | 0.04a |
| Nocardioidaceae | 0.02a | 0.02a | 0.02a | 0.02a | - | - | - | - | - | - | - | - | - | - | - | - |
| Rhizobiaceae | - | - | - | - | - | - | - | - | - | - | - | - | 0.01a | 0.01a | 0.01a | 0.01a |
| Rhodanobacteraceae | 0.05a | 0.04a | 0.02b | 0.01b | 0.03a | 0.03a | 0.02ab | 0.02b | 0.09a | 0.03ab | 0.02b | 0.02b | 0.07a | 0.04ab | 0.04ab | 0.01b |
| Saccharimonadales | 0.05ab | 0.06a | 0.04ab | 0.03b | 0.03a | 0.03a | 0.02ab | 0.02b | 0.02b | 0.03a | 0.02ab | 0.01c | 0.02a | 0.02a | 0.02a | 0.01b |
| SC-I-84 | - | - | - | - | 0.02b | 0.01b | 0.02b | 0.03a | 0.02a | 0.02a | 0.01a | 0.03a | 0.01bc | 0.01c | 0.02b | 0.03a |
| Solibacteraceae | 0.01b | 0.01b | 0.01b | 0.02a | 0.02a | 0.02a | 0.02a | 0.02a | 0.02a | 0.02a | 0.03a | 0.03a | 0.02a | 0.03a | 0.03a | 0.03a |
| Sphingobacteriaceae | 0.14ab | 0.17a | 0.14ab | 0.11b | - | - | - | - | - | - | - | - | - | - | - | - |
| Sphingomonadaceae | 0.07a | 0.05a | 0.08a | 0.09a | 0.07a | 0.08a | 0.07a | 0.06a | 0.08a | 0.07ab | 0.05b | 0.06b | 0.06ab | 0.05b | 0.07a | 0.08a |
| Subgroup_6 | - | - | - | - | 0.08a | 0.08a | 0.10a | 0.11a | 0.05b | 0.06b | 0.07ab | 0.09a | 0.03c | 0.04c | 0.08b | 0.12a |
| Xanthobacteraceae | 0.02b | 0.02b | 0.02b | 0.03a | 0.03a | 0.03a | 0.03a | 0.03a | 0.03a | 0.04a | 0.03a | 0.04a | 0.04ab | 0.04a | 0.04a | 0.03b |
| Xanthomonadaceae | 0.04a | 0.04a | 0.04a | 0.04a | - | - | - | - | - | - | - | - | - | - | - | - |

**Table S15. Significant difference analysis (p<0.05) of the top 11 common fungal families from different developmental stages**.

| Family | Germination stage | | | | Seedling stage | | | | Vigorous growth stage | | | | Harvest stage | | | |
| --- | --- | --- | --- | --- | --- | --- | --- | --- | --- | --- | --- | --- | --- | --- | --- | --- |
|  | T1 | T2 | T3 | T4 | T1 | T2 | T3 | T4 | T1 | T2 | T3 | T4 | T1 | T2 | T3 | T4 |
| Aspergillaceae | 0.03a | 0.04a | 0.05a | 0.04a | 0.05a | 0.05a | 0.04a | 0.10a | 0.01c | 0.01bc | 0.04ab | 0.04a | 0.06b | 0.08ab | 0.09a | 0.07ab |
| Chaetomiaceae | 0.44ab | 0.42ab | 0.52a | 0.24b | 0.07a | 0.07a | 0.06a | 0.06a | 0.02a | 0.02a | 0.05a | 0.05a | 0.06a | 0.05a | 0.05a | 0.06a |
| Cladosporiaceae | 0.01a | 0.02a | 0.02a | 0.02a | - | - | - | - | - | - | - | - | - | - | - | - |
| Cordycipitaceae | - | - | - | - | 0.02a | 0.01a | 0.01a | 0.01a | 0.00a | 0.00a | 0.01a | 0.02a | 0.04b | 0.06a | 0.06a | 0.06a |
| Cucurbitariaceae | - | - | - | - | - | - | - | - | - | - | - | - | 0.02a | 0.02a | 0.02a | 0.02a |
| Gymnoascaceae | - | - | - | - | 0.01a | 0.01a | 0.05a | 0.04a | - | - | - | - | - | - | - | - |
| Hypocreaceae | 0.02a | 0.02a | 0.01a | 0.02a | 0.02a | 0.02a | 0.01a | 0.02a | 0.01a | 0.02a | 0.04a | 0.02a | 0.04a | 0.04a | 0.02a | 0.02a |
| Hypocreales | 0.02a | 0.03a | 0.02a | 0.01a | - | - | - | - | - | - | - | - | - | - | - | - |
| Microascaceae | 0.03a | 0.04a | 0.03a | 0.03a | 0.02a | 0.02a | 0.01ab | 0.01b | 0.00a | 0.00a | 0.01a | 0.01a | - | - | - | - |
| Mortierellaceae | 0.01b | 0.01b | 0.01b | 0.05a | 0.02a | 0.01a | 0.02a | 0.03a | 0.05a | 0.02a | 0.04a | 0.06a | 0.02b | 0.02b | 0.02b | 0.04a |
| Nectriaceae | 0.14a | 0.10ab | 0.05b | 0.06ab | 0.04a | 0.03a | 0.02a | 0.04a | 0.01a | 0.01a | 0.07a | 0.03a | 0.05a | 0.05a | 0.05a | 0.05a |
| Pleosporaceae | 0.03a | 0.03a | 0.02a | 0.04a | 0.01a | 0.01a | 0.01a | 0.01a | - | - | - | - | - | - | - | - |
| Pseudeurotiaceae | 0.03a | 0.03a | 0.02a | 0.02a | - | - | - | - | - | - | - | - | - | - | - | - |
| Trichocomaceae | - | - | - | - | - | - | - | - | 0.11a | 0.04a | 0.02a | 0.03a | 0.02a | 0.02a | 0.02a | 0.02a |
| Trichosporonaceae | - | - | - | - | 0.03a | 0.03a | 0.04a | 0.03a | 0.01b | 0.01ab | 0.03a | 0.02ab | 0.03b | 0.04a | 0.04a | 0.04a |
| Trimorphomycetaceae | - | - | - | - | 0.02a | 0.01a | 0.03a | 0.06a | 0.00a | 0.01a | 0.02a | 0.03a | 0.04b | 0.07a | 0.07a | 0.08a |
| Unclassified | 0.10a | 0.10a | 0.09a | 0.17a | 0.34a | 0.32a | 0.45a | 0.40a | 0.70ab | 0.80a | 0.50b | 0.46b | 0.35a | 0.29a | 0.28a | 0.29a |

**Table S16. Taxonomic structure of the soil bacterial diversity at the genera level**.

| Germination stage | | | | | | | | | | | | | | | | | | | | | |
| --- | --- | --- | --- | --- | --- | --- | --- | --- | --- | --- | --- | --- | --- | --- | --- | --- | --- | --- | --- | --- | --- |
| Genus | A | | | | Genus | | B | | | | Genus | | C | | | | Genus | | D | | |
| Massilia | 0.11 | | | | Massilia | | 0.13 | | | | Massilia | | 0.10 | | | | Pedobacter | | 0.07 | | |
| Pedobacter | 0.07 | | | | Pedobacter | | 0.10 | | | | Pedobacter | | 0.09 | | | | Sphingomonas | | 0.07 | | |
| Mucilaginibacter | 0.07 | | | | Mucilaginibacter | | 0.07 | | | | Sphingomonas | | 0.07 | | | | Massilia | | 0.07 | | |
| Sphingomonas | 0.06 | | | | uncultured_bacterium_o_Saccharimonadales | | 0.06 | | | | uncultured_bacterium_f_Micrococcaceae | | 0.05 | | | | Gemmatimonas | | 0.04 | | |
| uncultured_bacterium_f_Micrococcaceae | 0.06 | | | | uncultured_bacterium_f_Micrococcaceae | | 0.06 | | | | Mucilaginibacter | | 0.05 | | | | Mucilaginibacter | | 0.04 | | |
| uncultured_bacterium_o_Saccharimonadales | 0.05 | | | | Sphingomonas | | 0.05 | | | | Gemmatimonas | | 0.05 | | | | uncultured_bacterium_f_Gemmatimonadaceae | | 0.03 | | |
| Gemmatimonas | 0.04 | | | | Gemmatimonas | | 0.04 | | | | uncultured_bacterium_o_Saccharimonadales | | 0.04 | | | | Luteimonas | | 0.03 | | |
| Rhodanobacter | 0.04 | | | | Rhodanobacter | | 0.03 | | | | Luteimonas | | 0.03 | | | | uncultured_bacterium_f_Micrococcaceae | | 0.03 | | |
| Luteimonas | 0.03 | | | | Luteimonas | | 0.03 | | | | uncultured_bacterium_f_Gemmatimonadaceae | | 0.02 | | | | uncultured_bacterium_c_Subgroup_6 | | 0.03 | | |
| uncultured_bacterium_f_Burkholderiaceae | 0.02 | | | | uncultured_bacterium_f_Burkholderiaceae | | 0.02 | | | | uncultured_bacterium_f_Chitinophagaceae | | 0.02 | | | | uncultured_bacterium_o_Saccharimonadales | | 0.03 | | |
| uncultured_bacterium_f_Chitinophagaceae | 0.02 | | | | Nocardioides | | 0.02 | | | | uncultured_bacterium_f_Isosphaeraceae | | 0.02 | | | | uncultured_bacterium_f_Burkholderiaceae | | 0.02 | | |
| uncultured_bacterium_f_Isosphaeraceae | 0.02 | | | | uncultured_bacterium_f_Xanthobacteraceae | | 0.02 | | | | uncultured_bacterium_f_Burkholderiaceae | | 0.02 | | | | uncultured_bacterium_f_Xanthobacteraceae | | 0.02 | | |
| Nocardioides | 0.02 | | | | Bacillus | | 0.02 | | | | Nocardioides | | 0.02 | | | | uncultured_bacterium_f_Isosphaeraceae | | 0.02 | | |
| uncultured_bacterium_f_Gemmatimonadaceae | 0.02 | | | | uncultured_bacterium_f_Chitinophagaceae | | 0.02 | | | | uncultured_bacterium_f_Xanthobacteraceae | | 0.02 | | | | uncultured_bacterium_o_Acidobacteriales | | 0.01 | | |
| Bacillus | 0.02 | | | | Paenibacillus | | 0.02 | | | | Rhodanobacter | | 0.02 | | | | uncultured_bacterium_o_Gaiellales | | 0.01 | | |
| uncultured_bacterium_f_Xanthobacteraceae | 0.02 | | | | uncultured_bacterium_f_Isosphaeraceae | | 0.01 | | | | Bacillus | | 0.01 | | | | uncultured_bacterium_o_C0119 | | 0.01 | | |
| uncultured_bacterium_o_Gaiellales | 0.02 | | | | uncultured_bacterium_f_Microbacteriaceae | | 0.01 | | | | uncultured_bacterium_o_Gaiellales | | 0.01 | | | | uncultured_bacterium_f_Sphingomonadaceae | | 0.01 | | |
| uncultured_bacterium_f_Microbacteriaceae | 0.01 | | | | Ramlibacter | | 0.01 | | | | uncultured_bacterium_f_Microbacteriaceae | | 0.01 | | | | Bryobacter | | 0.01 | | |
| Paenibacillus | 0.01 | | | | uncultured_bacterium_f_Gemmatimonadaceae | | 0.01 | | | | Paenibacillus | | 0.01 | | | | Nocardioides | | 0.01 | | |
| uncultured_bacterium_f_Sphingomonadaceae | 0.01 | | | | uncultured_bacterium_f_Longimicrobiaceae | | 0.01 | | | | uncultured_bacterium_f_Longimicrobiaceae | | 0.01 | | | | Bacillus | | 0.01 | | |
| other | 0.29 | | | | other | | 0.27 | | | | other | | 0.32 | | | | other | | 0.41 | | |
| Seedling stage | | | | | | | | | | | | | | | | | | | | | |
| Genus | | E | | | Genus | | | F | | | Genus | | | G | | | Genus | | | | H |
| uncultured_bacterium_c_Subgroup_6 | | 0.08 | | | uncultured_bacterium_c_Subgroup_6 | | | 0.08 | | | uncultured_bacterium_c_Subgroup_6 | | | 0.10 | | | uncultured_bacterium_c_Subgroup_6 | | | | 0.11 |
| uncultured_bacterium_o_Acidobacteriales | | 0.07 | | | uncultured_bacterium_o_Acidobacteriales | | | 0.08 | | | uncultured_bacterium_o_Acidobacteriales | | | 0.08 | | | uncultured_bacterium_o_Acidobacteriales | | | | 0.09 |
| Gemmatimonas | | 0.06 | | | Sphingomonas | | | 0.06 | | | uncultured_bacterium_f_Gemmatimonadaceae | | | 0.06 | | | uncultured_bacterium_f_Gemmatimonadaceae | | | | 0.06 |
| Sphingomonas | | 0.06 | | | Gemmatimonas | | | 0.06 | | | Sphingomonas | | | 0.06 | | | Sphingomonas | | | | 0.05 |
| uncultured_bacterium_f_Gemmatimonadaceae | | 0.05 | | | uncultured_bacterium_f_Gemmatimonadaceae | | | 0.05 | | | Gemmatimonas | | | 0.05 | | | Gemmatimonas | | | | 0.03 |
| uncultured_bacterium_o_Saccharimonadales | | 0.03 | | | uncultured_bacterium_o_Saccharimonadales | | | 0.03 | | | MND1 | | | 0.03 | | | uncultured_bacterium_o_Gaiellales | | | | 0.03 |
| uncultured_bacterium_o_Gaiellales | | 0.03 | | | uncultured_bacterium_o_Gaiellales | | | 0.03 | | | uncultured_bacterium_o_Gaiellales | | | 0.03 | | | uncultured_bacterium_f_SC-I-84 | | | | 0.03 |
| MND1 | | 0.02 | | | MND1 | | | 0.03 | | | Gemmatirosa | | | 0.03 | | | MND1 | | | | 0.02 |
| uncultured_bacterium_f_Xanthobacteraceae | | 0.02 | | | Gemmatirosa | | | 0.03 | | | uncultured_bacterium_o_Saccharimonadales | | | 0.02 | | | Gemmatirosa | | | | 0.02 |
| Haliangium | | 0.02 | | | Ellin6067 | | | 0.02 | | | uncultured_bacterium_f_Xanthobacteraceae | | | 0.02 | | | uncultured_bacterium_f_Xanthobacteraceae | | | | 0.02 |
| Ellin6067 | | 0.02 | | | uncultured_bacterium_o_Elsterales | | | 0.02 | | | uncultured_bacterium_f_SC-I-84 | | | 0.02 | | | uncultured_bacterium_o_Elsterales | | | | 0.02 |
| Gemmatirosa | | 0.02 | | | uncultured_bacterium_f_Xanthobacteraceae | | | 0.02 | | | Haliangium | | | 0.02 | | | Haliangium | | | | 0.02 |
| uncultured_bacterium_o_Elsterales | | 0.02 | | | Haliangium | | | 0.02 | | | uncultured_bacterium_o_Elsterales | | | 0.02 | | | uncultured_bacterium_o_Saccharimonadales | | | | 0.02 |
| uncultured_bacterium_f_SC-I-84 | | 0.02 | | | uncultured_bacterium_f_Acidobacteriaceae_Subgroup_1 | | | 0.01 | | | Ellin6067 | | | 0.01 | | | uncultured_bacterium_o_Rokubacteriales | | | | 0.01 |
| uncultured_bacterium_f_Acidobacteriaceae_Subgroup_1 | | 0.02 | | | uncultured_bacterium_f_Sphingomonadaceae | | | 0.01 | | | uncultured_bacterium_f_Sphingomonadaceae | | | 0.01 | | | Bryobacter | | | | 0.01 |
| uncultured_bacterium_f_Rhodanobacteraceae | | 0.01 | | | Mizugakiibacter | | | 0.01 | | | uncultured_bacterium_f_Acidobacteriaceae_Subgroup_1 | | | 0.01 | | | uncultured_bacterium_f_Acidobacteriaceae_Subgroup_1 | | | | 0.01 |
| Candidatus_Solibacter | | 0.01 | | | uncultured_bacterium_f_SC-I-84 | | | 0.01 | | | uncultured_bacterium_o_Subgroup_13 | | | 0.01 | | | Ellin6067 | | | | 0.01 |
| uncultured_bacterium_f_Sphingomonadaceae | | 0.01 | | | Candidatus_Solibacter | | | 0.01 | | | uncultured_bacterium_c_Alphaproteobacteria | | | 0.01 | | | Candidatus_Solibacter | | | | 0.01 |
| uncultured_bacterium_c_Alphaproteobacteria | | 0.01 | | | Pseudolabrys | | | 0.01 | | | Bryobacter | | | 0.01 | | | uncultured_bacterium_f_Sphingomonadaceae | | | | 0.01 |
| uncultured_bacterium_o_Subgroup_13 | | 0.01 | | | uncultured_bacterium_o_Subgroup_13 | | | 0.01 | | | uncultured_bacterium_f_Ruminococcaceae | | | 0.01 | | | uncultured_bacterium_o_Subgroup_2 | | | | 0.01 |
| other | | 0.42 | | | other | | | 0.39 | | | other | | | 0.40 | | | other | | | | 0.40 |
| Vigorous growth stage | | | | | | | | | | | | | | | | | | | | | |
| Genus | | | | I | | Genus | | | | J | | Genus | | | | K | | Genus | | L | |
| Sphingomonas | | | | 0.06 | | uncultured_bacterium_o_Acidobacteriales | | | | 0.09 | | uncultured_bacterium_o_Acidobacteriales | | | | 0.11 | | uncultured_bacterium_c_Subgroup_6 | | 0.09 | |
| Rhodanobacter | | | | 0.06 | | uncultured_bacterium_c_Subgroup_6 | | | | 0.06 | | uncultured_bacterium_f_Gemmatimonadaceae | | | | 0.07 | | uncultured_bacterium_o_Acidobacteriales | | 0.07 | |
| uncultured_bacterium_o_Acidobacteriales | | | | 0.06 | | Sphingomonas | | | | 0.06 | | uncultured_bacterium_c_Subgroup_6 | | | | 0.07 | | uncultured_bacterium_f_Gemmatimonadaceae | | 0.05 | |
| uncultured_bacterium_c_Subgroup_6 | | | | 0.05 | | uncultured_bacterium_f_Gemmatimonadaceae | | | | 0.05 | | Gemmatimonas | | | | 0.04 | | Sphingomonas | | 0.04 | |
| uncultured_bacterium_f_Gemmatimonadaceae | | | | 0.04 | | Gemmatimonas | | | | 0.05 | | Sphingomonas | | | | 0.04 | | uncultured_bacterium_f_Xanthobacteraceae | | 0.03 | |
| Gemmatimonas | | | | 0.04 | | uncultured_bacterium_o_Gaiellales | | | | 0.03 | | uncultured_bacterium_o_Gaiellales | | | | 0.03 | | uncultured_bacterium_f_SC-I-84 | | 0.03 | |
| uncultured_bacterium_o_Gaiellales | | | | 0.02 | | uncultured_bacterium_o_Saccharimonadales | | | | 0.03 | | Gemmatirosa | | | | 0.03 | | Gemmatimonas | | 0.02 | |
| Gemmatirosa | | | | 0.02 | | Gemmatirosa | | | | 0.03 | | MND1 | | | | 0.03 | | uncultured_bacterium_o_Rokubacteriales | | 0.02 | |
| uncultured_bacterium_o_Saccharimonadales | | | | 0.02 | | uncultured_bacterium_f_Xanthobacteraceae | | | | 0.02 | | uncultured_bacterium_o_Saccharimonadales | | | | 0.02 | | MND1 | | 0.02 | |
| uncultured_bacterium_f_Xanthobacteraceae | | | | 0.02 | | Candidatus_Solibacter | | | | 0.02 | | uncultured_bacterium_f_Xanthobacteraceae | | | | 0.02 | | Massilia | | 0.02 | |
| uncultured_bacterium_f_SC-I-84 | | | | 0.02 | | uncultured_bacterium_f_SC-I-84 | | | | 0.02 | | uncultured_bacterium_o_Subgroup_2 | | | | 0.02 | | Gemmatirosa | | 0.02 | |
| uncultured_bacterium_o_Chloroplast | | | | 0.02 | | MND1 | | | | 0.01 | | Haliangium | | | | 0.02 | | uncultured_bacterium_o_Gaiellales | | 0.02 | |
| uncultured_bacterium_f_Sphingomonadaceae | | | | 0.01 | | Haliangium | | | | 0.01 | | uncultured_bacterium_o_Elsterales | | | | 0.02 | | Haliangium | | 0.02 | |
| Candidatus_Solibacter | | | | 0.01 | | uncultured_bacterium_o_Elsterales | | | | 0.01 | | Burkholderia-Caballeronia-Paraburkholderia | | | | 0.01 | | Candidatus_Solibacter | | 0.02 | |
| uncultured_bacterium_f_Burkholderiaceae | | | | 0.01 | | Rhodanobacter | | | | 0.01 | | uncultured_bacterium_f_SC-I-84 | | | | 0.01 | | Microcoleus_Es-Yyy1400 | | 0.02 | |
| uncultured_bacterium_f_Acidobacteriaceae_Subgroup_1 | | | | 0.01 | | uncultured_bacterium_f_Acidobacteriaceae_Subgroup_1 | | | | 0.01 | | Candidatus_Solibacter | | | | 0.01 | | uncultured_bacterium_o_Elsterales | | 0.01 | |
| Haliangium | | | | 0.01 | | Ellin6067 | | | | 0.01 | | uncultured_bacterium_f_Acidobacteriaceae_Subgroup_1 | | | | 0.01 | | uncultured_bacterium_f_Sphingomonadaceae | | 0.01 | |
| MND1 | | | | 0.01 | | Microcoleus_Es-Yyy1400 | | | | 0.01 | | uncultured_bacterium_o_Subgroup_13 | | | | 0.01 | | uncultured_bacterium_o_Saccharimonadales | | 0.01 | |
| Dyella | | | | 0.01 | | Pseudolabrys | | | | 0.01 | | Bryobacter | | | | 0.01 | | uncultured_bacterium_c_Alphaproteobacteria | | 0.01 | |
| Bacillus | | | | 0.01 | | uncultured_bacterium_f_Sphingomonadaceae | | | | 0.01 | | uncultured_bacterium_f_Sphingomonadaceae | | | | 0.01 | | Dongia | | 0.01 | |
| other | | | | 0.47 | | other | | | | 0.43 | | other | | | | 0.41 | | other | | 0.45 | |
| Harvest stage | | | | | | | | | | | | | | | | | | | | | |
| Genus | | | M | | | Genus | | | N | | | Genus | | | O | | | Genus | | P | |
| Sphingomonas | | | 0.05 | | | uncultured_bacterium_o_Acidobacteriales | | | 0.06 | | | uncultured_bacterium_c_Subgroup_6 | | | 0.08 | | | uncultured_bacterium_c_Subgroup_6 | | 0.12 | |
| uncultured_bacterium_o_Acidobacteriales | | | 0.04 | | | uncultured_bacterium_f_Gemmatimonadaceae | | | 0.06 | | | uncultured_bacterium_o_Acidobacteriales | | | 0.06 | | | Sphingomonas | | 0.06 | |
| uncultured_bacterium_f_Gemmatimonadaceae | | | 0.04 | | | Sphingomonas | | | 0.04 | | | uncultured_bacterium_f_Gemmatimonadaceae | | | 0.06 | | | uncultured_bacterium_f_Gemmatimonadaceae | | 0.05 | |
| uncultured_bacterium_c_Subgroup_6 | | | 0.03 | | | uncultured_bacterium_c_Subgroup_6 | | | 0.04 | | | Sphingomonas | | | 0.05 | | | uncultured_bacterium_o_Acidobacteriales | | 0.05 | |
| uncultured_bacterium_f_Acidobacteriaceae_Subgroup_1 | | | 0.03 | | | uncultured_bacterium_f_Acidobacteriaceae_Subgroup_1 | | | 0.03 | | | Gemmatimonas | | | 0.03 | | | uncultured_bacterium_o_Rokubacteriales | | 0.05 | |
| Chujaibacter | | | 0.03 | | | uncultured_bacterium_o_Subgroup_2 | | | 0.03 | | | uncultured_bacterium_f_Xanthobacteraceae | | | 0.03 | | | uncultured_bacterium_f_SC-I-84 | | 0.03 | |
| Ralstonia | | | 0.03 | | | Gemmatirosa | | | 0.03 | | | Gemmatirosa | | | 0.03 | | | Gemmatimonas | | 0.03 | |
| Enterobacter | | | 0.03 | | | Gemmatimonas | | | 0.03 | | | uncultured_bacterium_o_Saccharimonadales | | | 0.02 | | | uncultured_bacterium_f_Xanthobacteraceae | | 0.03 | |
| Gemmatimonas | | | 0.03 | | | Burkholderia-Caballeronia-Paraburkholderia | | | 0.03 | | | Haliangium | | | 0.02 | | | MND1 | | 0.02 | |
| uncultured_bacterium_f_Xanthobacteraceae | | | 0.02 | | | uncultured_bacterium_f_Xanthobacteraceae | | | 0.02 | | | uncultured_bacterium_o_Gaiellales | | | 0.02 | | | Gemmatirosa | | 0.02 | |
| uncultured_bacterium_o_Saccharimonadales | | | 0.02 | | | uncultured_bacterium_o_Saccharimonadales | | | 0.02 | | | Rhodanobacter | | | 0.02 | | | Candidatus_Solibacter | | 0.02 | |
| uncultured_bacterium_o_Gaiellales | | | 0.02 | | | uncultured_bacterium_o_Gaiellales | | | 0.02 | | | Ralstonia | | | 0.02 | | | Ellin6067 | | 0.02 | |
| uncultured_bacterium_o_Chloroplast | | | 0.02 | | | Rhodanobacter | | | 0.02 | | | uncultured_bacterium_f_SC-I-84 | | | 0.02 | | | uncultured_bacterium_f_Micrococcaceae | | 0.02 | |
| Gemmatirosa | | | 0.02 | | | uncultured_bacterium_o_Elsterales | | | 0.02 | | | uncultured_bacterium_f_Acidobacteriaceae_Subgroup_1 | | | 0.01 | | | Haliangium | | 0.02 | |
| Rhodanobacter | | | 0.02 | | | Pseudolabrys | | | 0.02 | | | MND1 | | | 0.01 | | | uncultured_bacterium_f_Sphingomonadaceae | | 0.01 | |
| uncultured_bacterium_f_Burkholderiaceae | | | 0.02 | | | Candidatus_Solibacter | | | 0.01 | | | uncultured_bacterium_f_Sphingomonadaceae | | | 0.01 | | | uncultured_bacterium_c_Acidimicrobiia | | 0.01 | |
| uncultured_bacterium_o_Subgroup_2 | | | 0.01 | | | Haliangium | | | 0.01 | | | Candidatus_Solibacter | | | 0.01 | | | uncultured_bacterium_c_Alphaproteobacteria | | 0.01 | |
| uncultured_bacterium_o_Elsterales | | | 0.01 | | | Bryobacter | | | 0.01 | | | Ellin6067 | | | 0.01 | | | uncultured_bacterium_o_Gaiellales | | 0.01 | |
| Massilia | | | 0.01 | | | Enterobacter | | | 0.01 | | | Bryobacter | | | 0.01 | | | uncultured_bacterium_o_Subgroup_7 | | 0.01 | |
| Candidatus_Sulcia | | | 0.01 | | | Ralstonia | | | 0.01 | | | Pseudolabrys | | | 0.01 | | | Dongia | | 0.01 | |
| other | | | 0.50 | | | other | | | 0.49 | | | other | | | 0.46 | | | other | | 0.42 | |

A, E, I, M: soil treated with R. palustris PSB-06 and dazomet (T1); B, F, J, N: soil treated with dazomet alone (T2); C, G, K, O: soil treated with R. palustris PSB-06 alone (T3); D, H, L, P: soil without treatment (blank control, T4).

**Table S17. Taxonomic structure of the soil fungal diversity at the genera level**.

| Germination stage | | | | | | | | | | | | | | | | Seedling stage | | | | | | | | | | | | |  | |
| --- | --- | --- | --- | --- | --- | --- | --- | --- | --- | --- | --- | --- | --- | --- | --- | --- | --- | --- | --- | --- | --- | --- | --- | --- | --- | --- | --- | --- | --- | --- |
| Genus | | A | | Genus | | B | | Genus | | C | | Genus | | | D | Genus | | E | | Genus | F | | Genus | | G | | Genus | | H | |
| Botryotrichum | | 0.26 | | Botryotrichum | | 0.21 | | Botryotrichum | | 0.46 | | Unclassified | | | 0.27 | Unclassified | | 0.38 | | Unclassified | 0.35 | | Unclassified | | 0.47 | | Unclassified | | 0.43 | |
| Fusarium | | 0.14 | | Unclassified | | 0.15 | | Unclassified | | 0.10 | | Botryotrichum | | | 0.11 | Conocybe | | 0.09 | | Rhodotorula | 0.11 | | Conocybe | | 0.09 | | Aspergillus | | 0.06 | |
| Unclassified | | 0.12 | | Fusarium | | 0.10 | | Fusarium | | 0.05 | | Thielavia | | | 0.08 | Fusarium | | 0.04 | | Conocybe | 0.05 | | Gymnoascus | | 0.04 | | Saitozyma | | 0.06 | |
| Chaetomium | | 0.07 | | Thielavia | | 0.10 | | Aspergillus | | 0.04 | | Fusarium | | | 0.06 | Rhodotorula | | 0.03 | | Aspergillus | 0.03 | | Trichosporon | | 0.04 | | Gymnoascus | | 0.04 | |
| Ovatospora | | 0.05 | | Ovatospora | | 0.09 | | Gymnoascus | | 0.03 | | Mortierella | | | 0.05 | Aspergillus | | 0.03 | | Trichosporon | 0.03 | | Botryotrichum | | 0.03 | | Penicillium | | 0.04 | |
| Thielavia | | 0.04 | | Pseudogymnoascus | | 0.03 | | Thielavia | | 0.03 | | Alternaria | | | 0.04 | Trichosporon | | 0.03 | | Fusarium | 0.03 | | Saitozyma | | 0.03 | | Fusarium | | 0.03 | |
| Alternaria | | 0.03 | | Microascus | | 0.03 | | Microascus | | 0.03 | | Panaeolus | | | 0.03 | Mortierella | | 0.02 | | Trichoderma | 0.02 | | Mortierella | | 0.02 | | Trichosporon | | 0.03 | |
| Pseudogymnoascus | | 0.03 | | Alternaria | | 0.03 | | Alternaria | | 0.02 | | Aspergillus | | | 0.03 | Saitozyma | | 0.02 | | Cladosporium | 0.02 | | Penicillium | | 0.02 | | Mortierella | | 0.03 | |
| Microascus | | 0.02 | | Aspergillus | | 0.03 | | Sarocladium | | 0.02 | | Trichocladium | | | 0.02 | Trichoderma | | 0.02 | | Penicillium | 0.02 | | Fusarium | | 0.02 | | Cladosporium | | 0.02 | |
| Trichoderma | | 0.02 | | Trichoderma | | 0.02 | | Pseudogymnoascus | | 0.02 | | Microascus | | | 0.02 | Leptobacillium | | 0.02 | | Saitozyma | 0.01 | | Aspergillus | | 0.02 | | Trichoderma | | 0.02 | |
| Sarocladium | | 0.02 | | Sarocladium | | 0.02 | | Sporobolomyces | | 0.02 | | Tausonia | | | 0.02 | Talaromyces | | 0.02 | | Mortierella | 0.01 | | Trichoderma | | 0.01 | | Thielavia | | 0.02 | |
| Aspergillus | | 0.02 | | Cladosporium | | 0.02 | | Cercophora | | 0.02 | | Gymnoascus | | | 0.02 | Thielavia | | 0.01 | | Thielavia | 0.01 | | Alternaria | | 0.01 | | Botryotrichum | | 0.01 | |
| Penicillium | | 0.02 | | Oidiodendron | | 0.02 | | Cladosporium | | 0.02 | | Trichoderma | | | 0.02 | Botryotrichum | | 0.01 | | Leptobacillium | 0.01 | | Leptobacillium | | 0.01 | | Archaeorhizomyces | | 0.01 | |
| Cladosporium | | 0.01 | | Leucosporidium | | 0.02 | | Mortierella | | 0.01 | | Pseudogymnoascus | | | 0.02 | Gymnoascus | | 0.01 | | Botryotrichum | 0.01 | | Oidiodendron | | 0.01 | | Russula | | 0.01 | |
| Sporobolomyces | | 0.01 | | Penicillium | | 0.01 | | Trichoderma | | 0.01 | | Cladosporium | | | 0.02 | Penicillium | | 0.01 | | Gymnoascus | 0.01 | | Echria | | 0.01 | | Leptobacillium | | 0.01 | |
| Mortierella | | 0.01 | | Mortierella | | 0.01 | | Filobasidium | | 0.01 | | Ovatospora | | | 0.01 | Alternaria | | 0.01 | | Microascus | 0.01 | | Thielavia | | 0.01 | | Alternaria | | 0.01 | |
| Filobasidium | | 0.01 | | Sporobolomyces | | 0.01 | | Chaetomium | | 0.01 | | Penicillium | | | 0.01 | Cladosporium | | 0.01 | | Clitopilus | 0.01 | | Talaromyces | | 0.01 | | Microascus | | 0.01 | |
| Gymnoascus | | 0.01 | | Filobasidium | | 0.01 | | Ovatospora | | 0.01 | | Sarocladium | | | 0.01 | Ovatospora | | 0.01 | | Alternaria | 0.01 | | Russula | | 0.01 | | Oidiodendron | | 0.01 | |
| Oidiodendron | | 0.01 | | Gymnoascus | | 0.01 | | Penicillium | | 0.01 | | Plectosphaerella | | | 0.01 | Candida | | 0.01 | | Filobasidium | 0.01 | | Archaeorhizomyces | | 0.01 | | Cutaneotrichosporon | | 0.00 | |
| Leucosporidium | | 0.01 | | Lasiobolidium | | 0.00 | | Leucosporidium | | 0.01 | | Pseudaleuria | | | 0.01 | Clitopilus | | 0.01 | | Talaromyces | 0.01 | | Cladosporium | | 0.01 | | Trichocladium | | 0.00 | |
| other | | 0.08 | | other | | 0.06 | | other | | 0.07 | | other | | | 0.13 | other | | 0.23 | | other | 0.23 | | other | | 0.13 | | other | | 0.16 | |
| Vigorous growth stage | | | | | | | | | | | | | | Harvest stage | | | | | | | | | | | | | | | | |
| Genus | I | | Genus | | J | | Genus | | K | | Genus | | L | Genus | | | M | | Genus | | | N | | Genus | | O | | Genus | | P |
| Unclassified | 0.71 | | Unclassified | | 0.80 | | Unclassified | | 0.52 | | Unclassified | | 0.49 | Unclassified | | | 0.39 | | Unclassified | | | 0.33 | | Unclassified | | 0.32 | | Unclassified | | 0.33 |
| Talaromyces | 0.11 | | Talaromyces | | 0.04 | | Fusarium | | 0.07 | | Mortierella | | 0.06 | Fusarium | | | 0.04 | | Saitozyma | | | 0.07 | | Saitozyma | | 0.07 | | Saitozyma | | 0.08 |
| Mortierella | 0.05 | | Mortierella | | 0.02 | | Mortierella | | 0.04 | | Coprinellus | | 0.03 | Aspergillus | | | 0.04 | | Leptobacillium | | | 0.05 | | Aspergillus | | 0.05 | | Leptobacillium | | 0.05 |
| Fusarium | 0.01 | | Trichoderma | | 0.02 | | Trichoderma | | 0.04 | | Fusarium | | 0.03 | Saitozyma | | | 0.04 | | Aspergillus | | | 0.05 | | Leptobacillium | | 0.05 | | Aspergillus | | 0.04 |
| Conocybe | 0.01 | | Fusarium | | 0.01 | | Trichosporon | | 0.03 | | Saitozyma | | 0.03 | Trichoderma | | | 0.04 | | Trichoderma | | | 0.04 | | Fusarium | | 0.04 | | Fusarium | | 0.04 |
| Trichoderma | 0.01 | | Aspergillus | | 0.01 | | Aspergillus | | 0.02 | | Talaromyces | | 0.03 | Leptobacillium | | | 0.03 | | Fusarium | | | 0.04 | | Trichosporon | | 0.03 | | Mortierella | | 0.04 |
| Microdochium | 0.01 | | Trichosporon | | 0.01 | | Saitozyma | | 0.02 | | Aspergillus | | 0.02 | Trichosporon | | | 0.02 | | Trichosporon | | | 0.03 | | Penicillium | | 0.03 | | Trichosporon | | 0.03 |
| Botryotrichum | 0.01 | | Saitozyma | | 0.01 | | Talaromyces | | 0.02 | | Pseudaleuria | | 0.02 | Filobasidium | | | 0.02 | | Pyrenochaeta | | | 0.02 | | Pyrenochaeta | | 0.02 | | Penicillium | | 0.02 |
| Gymnoascus | 0.01 | | Filobasidium | | 0.01 | | Gymnoascus | | 0.01 | | Trichoderma | | 0.02 | Mortierella | | | 0.02 | | Penicillium | | | 0.02 | | Mortierella | | 0.02 | | Pyrenochaeta | | 0.02 |
| Aspergillus | 0.01 | | Botryotrichum | | 0.00 | | Penicillium | | 0.01 | | Trichosporon | | 0.02 | Cladosporium | | | 0.02 | | Plectosphaerella | | | 0.02 | | Plectosphaerella | | 0.02 | | Plectosphaerella | | 0.02 |
| Trichosporon | 0.00 | | Penicillium | | 0.00 | | Botryotrichum | | 0.01 | | Leptobacillium | | 0.02 | Talaromyces | | | 0.02 | | Mortierella | | | 0.02 | | Talaromyces | | 0.02 | | Archaeorhizomyces | | 0.02 |
| Saitozyma | 0.00 | | Archaeorhizomyces | | 0.00 | | Filobasidium | | 0.01 | | Filobasidium | | 0.01 | Penicillium | | | 0.02 | | Archaeorhizomyces | | | 0.02 | | Trichoderma | | 0.02 | | Trichoderma | | 0.02 |
| Plectosphaerella | 0.00 | | Leptobacillium | | 0.00 | | Cladosporium | | 0.01 | | Penicillium | | 0.01 | Mycothermus | | | 0.02 | | Talaromyces | | | 0.02 | | Cladosporium | | 0.02 | | Talaromyces | | 0.02 |
| Penicillium | 0.00 | | Cladosporium | | 0.00 | | Leptobacillium | | 0.01 | | Thielavia | | 0.01 | Pyrenochaeta | | | 0.02 | | Cladosporium | | | 0.01 | | Archaeorhizomyces | | 0.01 | | Botryotrichum | | 0.01 |
| Ovatospora | 0.00 | | Russula | | 0.00 | | Condenascus | | 0.01 | | Trichocladium | | 0.01 | Plectosphaerella | | | 0.01 | | Botryotrichum | | | 0.01 | | Botryotrichum | | 0.01 | | Cladosporium | | 0.01 |
| Sarocladium | 0.00 | | Microdochium | | 0.00 | | Humicola | | 0.01 | | Solicoccozyma | | 0.01 | Alternaria | | | 0.01 | | Chaetomium | | | 0.01 | | Chaetomium | | 0.01 | | Cercophora | | 0.01 |
| Thielavia | 0.00 | | Candida | | 0.00 | | Candida | | 0.01 | | Cladosporium | | 0.01 | Botryotrichum | | | 0.01 | | Cercophora | | | 0.01 | | Cercophora | | 0.01 | | Chaetomium | | 0.01 |
| Leptobacillium | 0.00 | | Oidiodendron | | 0.00 | | Plectosphaerella | | 0.01 | | Pyrenochaeta | | 0.01 | Candida | | | 0.01 | | Humicola | | | 0.01 | | Humicola | | 0.01 | | Humicola | | 0.01 |
| Rhodotorula | 0.00 | | Thielavia | | 0.00 | | Trichocladium | | 0.00 | | Plectosphaerella | | 0.01 | Humicola | | | 0.01 | | Coprinellus | | | 0.01 | | Coprinellus | | 0.01 | | Arachnomyces | | 0.01 |
| Oidiodendron | 0.00 | | Alternaria | | 0.00 | | Russula | | 0.00 | | Mycothermus | | 0.01 | Archaeorhizomyces | | | 0.01 | | Russula | | | 0.01 | | Alternaria | | 0.01 | | Nigrospora | | 0.01 |
| other | 0.04 | | other | | 0.05 | | other | | 0.16 | | other | | 0.16 | other | | | 0.21 | | other | | | 0.20 | | other | | 0.21 | | other | | 0.21 |

A, E, I, M: soil treated with R. palustris PSB-06 and dazomet (T1); B, F, J, N: soil treated with dazomet alone (T2); C, G, K, O: soil treated with R. palustris PSB-06 alone (T3); D, H, L, P: soil without treatment (blank control, T4).

**Table S18. Detrended correspondence analysis (DCA) indices of soil microorganisms.**

| DCA | x | |
| --- | --- | --- |
|  | Bacteria | Fungi |
| DCA1 | 2.338149343 | 2.243258031 |
| DCA2 | 1.427947365 | 1.572386335 |
| DCA3 | 1.293627304 | 0.91088826 |
| DCA4 | 1.116702998 | 0.805775192 |

**Table S19. Mantel test of soil bacteria at the phylum level with soil physicochemical properties.**

| Phylum | Soil physicochemical properties | r | p.value | Phylum | Soil physicochemical properties | r | p.value |
| --- | --- | --- | --- | --- | --- | --- | --- |
| Acidobacteria | pH | 0.049526 | >=0.05 | Gemmatimonadetes | pH | -0.076784 | >=0.05 |
| Acidobacteria | OM | -0.11504 | >=0.05 | Gemmatimonadetes | OM | 0.0670045 | >=0.05 |
| Acidobacteria | TN | -0.11551 | >=0.05 | Gemmatimonadetes | TN | 0.1544559 | >=0.05 |
| Acidobacteria | AN | 0.066004 | >=0.05 | Gemmatimonadetes | AN | 0.0846295 | >=0.05 |
| Acidobacteria | TP | -0.03092 | >=0.05 | Gemmatimonadetes | TP | 0.0181989 | >=0.05 |
| Acidobacteria | AP | 0.096397 | >=0.05 | Gemmatimonadetes | AP | -0.04463 | >=0.05 |
| Acidobacteria | TK | -0.05466 | >=0.05 | Gemmatimonadetes | TK | -0.049793 | >=0.05 |
| Acidobacteria | AK | -0.07272 | >=0.05 | Gemmatimonadetes | AK | -0.054211 | >=0.05 |
| Actinobacteria | pH | 0.133041 | >=0.05 | Latescibacteria | pH | 0.3085402 | 0.001-0.01 |
| Actinobacteria | OM | -0.00275 | >=0.05 | Latescibacteria | OM | 0.1622892 | >=0.05 |
| Actinobacteria | TN | 0.131701 | >=0.05 | Latescibacteria | TN | 0.2990944 | 0.001-0.01 |
| Actinobacteria | AN | -0.01596 | >=0.05 | Latescibacteria | AN | 0.052593 | >=0.05 |
| Actinobacteria | TP | 0.004186 | >=0.05 | Latescibacteria | TP | -0.134027 | >=0.05 |
| Actinobacteria | AP | 0.143581 | >=0.05 | Latescibacteria | AP | 0.042755 | >=0.05 |
| Actinobacteria | TK | -0.02137 | >=0.05 | Latescibacteria | TK | -0.038313 | >=0.05 |
| Actinobacteria | AK | -0.02768 | >=0.05 | Latescibacteria | AK | -0.031134 | >=0.05 |
| Bacteroidetes | pH | 0.437514 | 0.001-0.01 | Nitrospirae | pH | 0.2608524 | 0.01-0.05 |
| Bacteroidetes | OM | -0.06064 | >=0.05 | Nitrospirae | OM | -0.024556 | >=0.05 |
| Bacteroidetes | TN | 0.106545 | >=0.05 | Nitrospirae | TN | 0.1571309 | >=0.05 |
| Bacteroidetes | AN | 0.022537 | >=0.05 | Nitrospirae | AN | 0.0407357 | >=0.05 |
| Bacteroidetes | TP | 0.02755 | >=0.05 | Nitrospirae | TP | 0.074379 | >=0.05 |
| Bacteroidetes | AP | 0.259957 | 0.001-0.01 | Nitrospirae | AP | 0.346024 | 0.001-0.01 |
| Bacteroidetes | TK | 0.301124 | 0.001-0.01 | Nitrospirae | TK | 0.1702984 | 0.01-0.05 |
| Bacteroidetes | AK | -0.00652 | >=0.05 | Nitrospirae | AK | 0.0654083 | >=0.05 |
| Chloroflexi | pH | 0.120669 | >=0.05 | Patescibacteria | pH | 0.6221774 | 0.001-0.01 |
| Chloroflexi | OM | -0.01959 | >=0.05 | Patescibacteria | OM | -0.056356 | >=0.05 |
| Chloroflexi | TN | 0.236176 | 0.01-0.05 | Patescibacteria | TN | 0.0798546 | >=0.05 |
| Chloroflexi | AN | 0.216542 | 0.01-0.05 | Patescibacteria | AN | 0.0928924 | >=0.05 |
| Chloroflexi | TP | 0.096177 | >=0.05 | Patescibacteria | TP | -0.062594 | >=0.05 |
| Chloroflexi | AP | -0.10536 | >=0.05 | Patescibacteria | AP | 0.724472 | 0.001-0.01 |
| Chloroflexi | TK | -0.02947 | >=0.05 | Patescibacteria | TK | 0.2611348 | 0.001-0.01 |
| Chloroflexi | AK | 0.099258 | >=0.05 | Patescibacteria | AK | 0.0336122 | >=0.05 |
| Cyanobacteria | pH | -0.00229 | >=0.05 | Planctomycetes | pH | -0.056763 | >=0.05 |
| Cyanobacteria | OM | 0.008729 | >=0.05 | Planctomycetes | OM | -0.162406 | >=0.05 |
| Cyanobacteria | TN | -0.01879 | >=0.05 | Planctomycetes | TN | -0.032135 | >=0.05 |
| Cyanobacteria | AN | -0.07848 | >=0.05 | Planctomycetes | AN | 0.0550231 | >=0.05 |
| Cyanobacteria | TP | -0.0669 | >=0.05 | Planctomycetes | TP | 0.3737972 | 0.001-0.01 |
| Cyanobacteria | AP | -0.005 | >=0.05 | Planctomycetes | AP | -0.06044 | >=0.05 |
| Cyanobacteria | TK | -0.06327 | >=0.05 | Planctomycetes | TK | 0.0043709 | >=0.05 |
| Cyanobacteria | AK | -0.05772 | >=0.05 | Planctomycetes | AK | 0.0354844 | >=0.05 |
| Dependentiae | pH | 0.54258 | 0.001-0.01 | Proteobacteria | pH | 0.148293 | >=0.05 |
| Dependentiae | OM | 0.12492 | >=0.05 | Proteobacteria | OM | 0.0470346 | >=0.05 |
| Dependentiae | TN | 0.025122 | >=0.05 | Proteobacteria | TN | 0.2019662 | >=0.05 |
| Dependentiae | AN | -0.04998 | >=0.05 | Proteobacteria | AN | 0.0871697 | >=0.05 |
| Dependentiae | TP | 0.018539 | >=0.05 | Proteobacteria | TP | 0.0316627 | >=0.05 |
| Dependentiae | AP | 0.738739 | 0.001-0.01 | Proteobacteria | AP | 0.2232327 | >=0.05 |
| Dependentiae | TK | 0.093525 | >=0.05 | Proteobacteria | TK | 0.2100927 | 0.01-0.05 |
| Dependentiae | AK | 0.010115 | >=0.05 | Proteobacteria | AK | -0.032892 | >=0.05 |
| Elusimicrobia | pH | 0.018792 | >=0.05 | Rokubacteria | pH | 0.7072941 | 0.001-0.01 |
| Elusimicrobia | OM | 0.199803 | >=0.05 | Rokubacteria | OM | -0.075818 | >=0.05 |
| Elusimicrobia | TN | 0.390927 | 0.001-0.01 | Rokubacteria | TN | 0.0927748 | >=0.05 |
| Elusimicrobia | AN | 0.168709 | >=0.05 | Rokubacteria | AN | -0.058552 | >=0.05 |
| Elusimicrobia | TP | -0.03878 | >=0.05 | Rokubacteria | TP | -0.072317 | >=0.05 |
| Elusimicrobia | AP | -0.02298 | >=0.05 | Rokubacteria | AP | 0.6605322 | 0.001-0.01 |
| Elusimicrobia | TK | -0.04806 | >=0.05 | Rokubacteria | TK | 0.1234844 | >=0.05 |
| Elusimicrobia | AK | 0.092949 | >=0.05 | Rokubacteria | AK | -0.060243 | >=0.05 |
| FCPU426 | pH | -0.10946 | >=0.05 | Verrucomicrobia | pH | -0.027682 | >=0.05 |
| FCPU426 | OM | 0.062337 | >=0.05 | Verrucomicrobia | OM | -0.007854 | >=0.05 |
| FCPU426 | TN | 0.228373 | 0.01-0.05 | Verrucomicrobia | TN | -0.175761 | >=0.05 |
| FCPU426 | AN | 0.227438 | 0.01-0.05 | Verrucomicrobia | AN | -0.110184 | >=0.05 |
| FCPU426 | TP | -0.06664 | >=0.05 | Verrucomicrobia | TP | 0.2459832 | >=0.05 |
| FCPU426 | AP | -0.05483 | >=0.05 | Verrucomicrobia | AP | -0.02188 | >=0.05 |
| FCPU426 | TK | 0.077514 | >=0.05 | Verrucomicrobia | TK | -0.069732 | >=0.05 |
| FCPU426 | AK | 0.228216 | 0.01-0.05 | Verrucomicrobia | AK | -0.110866 | >=0.05 |
| Firmicutes | pH | 0.645319 | 0.001-0.01 | WPS-2 | pH | 0.1625421 | >=0.05 |
| Firmicutes | OM | -0.04303 | >=0.05 | WPS-2 | OM | 0.1687417 | >=0.05 |
| Firmicutes | TN | 0.173051 | >=0.05 | WPS-2 | TN | 0.1683702 | >=0.05 |
| Firmicutes | AN | -0.05266 | >=0.05 | WPS-2 | AN | 0.0045636 | >=0.05 |
| Firmicutes | TP | 0.142803 | >=0.05 | WPS-2 | TP | -0.145678 | >=0.05 |
| Firmicutes | AP | 0.363828 | 0.001-0.01 | WPS-2 | AP | 0.2186947 | >=0.05 |
| Firmicutes | TK | 0.135991 | >=0.05 | WPS-2 | TK | -0.052797 | >=0.05 |
| Firmicutes | AK | 0.00803 | >=0.05 | WPS-2 | AK | -0.104648 | >=0.05 |
| Fusobacteria | pH | 0.228425 | 0.01-0.05 | uncultured_bacterium_k_Bacteria | pH | 0.2346695 | 0.01-0.05 |
| Fusobacteria | OM | -0.0393 | >=0.05 | uncultured_bacterium_k_Bacteria | OM | 0.1734773 | >=0.05 |
| Fusobacteria | TN | 0.206506 | >=0.05 | uncultured_bacterium_k_Bacteria | TN | 0.4799751 | 0.001-0.01 |
| Fusobacteria | AN | 0.034079 | >=0.05 | uncultured_bacterium_k_Bacteria | AN | 0.1982572 | 0.01-0.05 |
| Fusobacteria | TP | 0.182752 | >=0.05 | uncultured_bacterium_k_Bacteria | TP | 0.0240066 | >=0.05 |
| Fusobacteria | AP | 0.010543 | >=0.05 | uncultured_bacterium_k_Bacteria | AP | 0.08241 | >=0.05 |
| Fusobacteria | TK | 0.013067 | >=0.05 | uncultured_bacterium_k_Bacteria | TK | 0.0182389 | >=0.05 |
| Fusobacteria | AK | 0.112629 | >=0.05 | uncultured_bacterium_k_Bacteria | AK | 0.0602223 | >=0.05 |

OM, organic matter; TN, total nitrogen; AN, hydrolytic nitrogen; TP, total phosphorus; AP, available phosphorus; TK, total potassium; and AK, available potassium.

**Table S20. Mantel test of soil fungi at the phylum level with soil physicochemical properties.**

| Phylum | Soil physicochemical properties | r | p.value |
| --- | --- | --- | --- |
| Ascomycota | pH | 0.37024 | 0.001-0.01 |
| Ascomycota | OM | -0.01475 | >=0.05 |
| Ascomycota | TN | 0.001092 | >=0.05 |
| Ascomycota | AN | 0.050702 | >=0.05 |
| Ascomycota | TP | -0.0238 | >=0.05 |
| Ascomycota | AP | 0.565303 | 0.001-0.01 |
| Ascomycota | TK | -0.01728 | >=0.05 |
| Ascomycota | AK | -0.03175 | >=0.05 |
| Basidiomycota | pH | 0.495981 | 0.001-0.01 |
| Basidiomycota | OM | -0.04452 | >=0.05 |
| Basidiomycota | TN | 0.072475 | >=0.05 |
| Basidiomycota | AN | -0.16372 | >=0.05 |
| Basidiomycota | TP | -0.04319 | >=0.05 |
| Basidiomycota | AP | 0.690739 | 0.001-0.01 |
| Basidiomycota | TK | 0.144033 | >=0.05 |
| Basidiomycota | AK | -0.14956 | >=0.05 |
| Chytridiomycota | pH | 0.066134 | >=0.05 |
| Chytridiomycota | OM | -0.08763 | >=0.05 |
| Chytridiomycota | TN | 0.066507 | >=0.05 |
| Chytridiomycota | AN | -0.01852 | >=0.05 |
| Chytridiomycota | TP | -0.09289 | >=0.05 |
| Chytridiomycota | AP | 0.096561 | >=0.05 |
| Chytridiomycota | TK | 0.193388 | 0.01-0.05 |
| Chytridiomycota | AK | 0.027916 | >=0.05 |
| Glomeromycota | pH | -0.13434 | >=0.05 |
| Glomeromycota | OM | -0.13967 | >=0.05 |
| Glomeromycota | TN | 0.064719 | >=0.05 |
| Glomeromycota | AN | -0.13929 | >=0.05 |
| Glomeromycota | TP | -0.09001 | >=0.05 |
| Glomeromycota | AP | -0.10737 | >=0.05 |
| Glomeromycota | TK | 0.139252 | >=0.05 |
| Glomeromycota | AK | -0.11063 | >=0.05 |
| Mortierellomycota | pH | 0.104405 | >=0.05 |
| Mortierellomycota | OM | -0.06615 | >=0.05 |
| Mortierellomycota | TN | 0.088261 | >=0.05 |
| Mortierellomycota | AN | -0.17073 | >=0.05 |
| Mortierellomycota | TP | -0.13747 | >=0.05 |
| Mortierellomycota | AP | 0.12079 | >=0.05 |
| Mortierellomycota | TK | 0.087414 | >=0.05 |
| Mortierellomycota | AK | -0.15051 | >=0.05 |
| Mucoromycota | pH | 0.637382 | 0.001-0.01 |
| Mucoromycota | OM | -0.06538 | >=0.05 |
| Mucoromycota | TN | 0.049534 | >=0.05 |
| Mucoromycota | AN | -0.03738 | >=0.05 |
| Mucoromycota | TP | 0.044037 | >=0.05 |
| Mucoromycota | AP | 0.613356 | 0.001-0.01 |
| Mucoromycota | TK | 0.263211 | 0.01-0.05 |
| Mucoromycota | AK | -0.12501 | >=0.05 |
| Olpidiomycota | pH | 0.000763 | >=0.05 |
| Olpidiomycota | OM | -0.13659 | >=0.05 |
| Olpidiomycota | TN | -0.03956 | >=0.05 |
| Olpidiomycota | AN | -0.00482 | >=0.05 |
| Olpidiomycota | TP | 0.138726 | >=0.05 |
| Olpidiomycota | AP | 0.015455 | >=0.05 |
| Olpidiomycota | TK | -0.11948 | >=0.05 |
| Olpidiomycota | AK | -0.00131 | >=0.05 |
| Rozellomycota | pH | 0.231903 | 0.01-0.05 |
| Rozellomycota | OM | -0.08946 | >=0.05 |
| Rozellomycota | TN | -0.06359 | >=0.05 |
| Rozellomycota | AN | 0.049076 | >=0.05 |
| Rozellomycota | TP | -0.05278 | >=0.05 |
| Rozellomycota | AP | 0.308749 | 0.01-0.05 |
| Rozellomycota | TK | 0.169961 | 0.01-0.05 |
| Rozellomycota | AK | -0.04036 | >=0.05 |
| Unclassified | pH | 0.257344 | 0.01-0.05 |
| Unclassified | OM | -0.13314 | >=0.05 |
| Unclassified | TN | -0.09718 | >=0.05 |
| Unclassified | AN | -0.13196 | >=0.05 |
| Unclassified | TP | 0.166092 | >=0.05 |
| Unclassified | AP | 0.416339 | 0.001-0.01 |
| Unclassified | TK | 0.004545 | >=0.05 |
| Unclassified | AK | -0.13535 | >=0.05 |

OM, organic matter; TN, total nitrogen; AN, hydrolytic nitrogen; TP, total phosphorus; AP, available phosphorus; TK, total potassium; and AK, available potassium.

**Table S21. Mantel test of soil bacteria at the family level with soil physicochemical properties**.

| spec | env | r | p.value |
| --- | --- | --- | --- |
| Burkholderiaceae | pH | 0.15 | 0.04 |
| Burkholderiaceae | OM | -0.04 | 0.68 |
| Burkholderiaceae | TN | 0.05 | 0.22 |
| Burkholderiaceae | AN | -0.06 | 0.82 |
| Burkholderiaceae | TP | -0.07 | 0.83 |
| Burkholderiaceae | AP | 0.12 | 0.07 |
| Burkholderiaceae | TK | 0.16 | 0.03 |
| Burkholderiaceae | AK | -0.04 | 0.68 |
| Elsterales | pH | 0.34 | 0.00 |
| Elsterales | OM | 0.12 | 0.16 |
| Elsterales | TN | 0.41 | 0.00 |
| Elsterales | AN | -0.09 | 0.76 |
| Elsterales | TP | 0.06 | 0.28 |
| Elsterales | AP | 0.26 | 0.02 |
| Elsterales | TK | -0.01 | 0.50 |
| Elsterales | AK | -0.02 | 0.56 |
| Gaiellales | pH | 0.30 | 0.01 |
| Gaiellales | OM | 0.07 | 0.20 |
| Gaiellales | TN | 0.30 | 0.01 |
| Gaiellales | AN | 0.04 | 0.29 |
| Gaiellales | TP | -0.03 | 0.58 |
| Gaiellales | AP | 0.36 | 0.00 |
| Gaiellales | TK | 0.15 | 0.04 |
| Gaiellales | AK | 0.02 | 0.33 |
| Gemmatimonadaceae | pH | -0.07 | 0.76 |
| Gemmatimonadaceae | OM | 0.09 | 0.17 |
| Gemmatimonadaceae | TN | 0.12 | 0.09 |
| Gemmatimonadaceae | AN | 0.08 | 0.20 |
| Gemmatimonadaceae | TP | 0.01 | 0.44 |
| Gemmatimonadaceae | AP | -0.03 | 0.59 |
| Gemmatimonadaceae | TK | -0.04 | 0.67 |
| Gemmatimonadaceae | AK | -0.06 | 0.73 |
| Haliangiaceae | pH | -0.09 | 0.83 |
| Haliangiaceae | OM | 0.21 | 0.06 |
| Haliangiaceae | TN | 0.50 | 0.00 |
| Haliangiaceae | AN | 0.17 | 0.08 |
| Haliangiaceae | TP | -0.01 | 0.51 |
| Haliangiaceae | AP | -0.13 | 0.89 |
| Haliangiaceae | TK | -0.07 | 0.74 |
| Haliangiaceae | AK | 0.21 | 0.03 |
| Nitrosomonadaceae | pH | 0.22 | 0.03 |
| Nitrosomonadaceae | OM | 0.11 | 0.14 |
| Nitrosomonadaceae | TN | 0.31 | 0.01 |
| Nitrosomonadaceae | AN | 0.04 | 0.30 |
| Nitrosomonadaceae | TP | -0.15 | 0.95 |
| Nitrosomonadaceae | AP | 0.05 | 0.25 |
| Nitrosomonadaceae | TK | -0.02 | 0.49 |
| Nitrosomonadaceae | AK | -0.07 | 0.80 |
| Rhodanobacteraceae | pH | 0.38 | 0.00 |
| Rhodanobacteraceae | OM | -0.11 | 0.93 |
| Rhodanobacteraceae | TN | 0.20 | 0.03 |
| Rhodanobacteraceae | AN | -0.09 | 0.85 |
| Rhodanobacteraceae | TP | -0.06 | 0.74 |
| Rhodanobacteraceae | AP | 0.36 | 0.01 |
| Rhodanobacteraceae | TK | 0.24 | 0.01 |
| Rhodanobacteraceae | AK | -0.02 | 0.55 |
| Saccharimonadales | pH | 0.63 | 0.00 |
| Saccharimonadales | OM | -0.06 | 0.71 |
| Saccharimonadales | TN | 0.08 | 0.16 |
| Saccharimonadales | AN | 0.09 | 0.15 |
| Saccharimonadales | TP | -0.06 | 0.75 |
| Saccharimonadales | AP | 0.72 | 0.00 |
| Saccharimonadales | TK | 0.26 | 0.01 |
| Saccharimonadales | AK | 0.03 | 0.30 |
| Sphingomonadaceae | pH | -0.08 | 0.78 |
| Sphingomonadaceae | OM | 0.38 | 0.01 |
| Sphingomonadaceae | TN | 0.48 | 0.00 |
| Sphingomonadaceae | AN | 0.12 | 0.14 |
| Sphingomonadaceae | TP | -0.04 | 0.60 |
| Sphingomonadaceae | AP | -0.13 | 0.89 |
| Sphingomonadaceae | TK | -0.13 | 0.96 |
| Sphingomonadaceae | AK | 0.06 | 0.24 |
| Xanthobacteraceae | pH | 0.21 | 0.03 |
| Xanthobacteraceae | OM | 0.17 | 0.08 |
| Xanthobacteraceae | TN | 0.22 | 0.03 |
| Xanthobacteraceae | AN | 0.07 | 0.25 |
| Xanthobacteraceae | TP | 0.05 | 0.30 |
| Xanthobacteraceae | AP | 0.35 | 0.01 |
| Xanthobacteraceae | TK | 0.10 | 0.15 |
| Xanthobacteraceae | AK | 0.05 | 0.26 |

OM, organic matter; TN, total nitrogen; AN, hydrolytic nitrogen; TP, total phosphorus; AP, available phosphorus; TK, total potassium; and AK, available potassium.

**Table S22. Mantel test of soil fungi at the family level with soil physicochemical properties**.

| spec | env | r | p.value |
| --- | --- | --- | --- |
| Aspergillaceae | pH | 0.146362 | 0.071 |
| Aspergillaceae | OM | -0.08448 | 0.831 |
| Aspergillaceae | TN | -0.03262 | 0.58 |
| Aspergillaceae | AN | 0.048889 | 0.286 |
| Aspergillaceae | TP | -0.00334 | 0.445 |
| Aspergillaceae | AP | 0.309947 | 0.013 |
| Aspergillaceae | TK | -0.02279 | 0.529 |
| Aspergillaceae | AK | 0.048211 | 0.252 |
| Mortierellaceae | pH | 0.068788 | 0.251 |
| Mortierellaceae | OM | -0.09365 | 0.736 |
| Mortierellaceae | TN | 0.104121 | 0.21 |
| Mortierellaceae | AN | -0.13399 | 0.884 |
| Mortierellaceae | TP | -0.13801 | 0.891 |
| Mortierellaceae | AP | 0.098169 | 0.19 |
| Mortierellaceae | TK | 0.078027 | 0.184 |
| Mortierellaceae | AK | -0.16412 | 0.991 |
| Ophiocordycipitaceae | pH | -0.12008 | 0.905 |
| Ophiocordycipitaceae | OM | -0.09317 | 0.701 |
| Ophiocordycipitaceae | TN | 0.127884 | 0.18 |
| Ophiocordycipitaceae | AN | -0.12274 | 0.828 |
| Ophiocordycipitaceae | TP | -0.09034 | 0.705 |
| Ophiocordycipitaceae | AP | -0.10638 | 0.786 |
| Ophiocordycipitaceae | TK | 0.112747 | 0.167 |
| Ophiocordycipitaceae | AK | -0.12803 | 0.953 |
| Trichosporonaceae | pH | 0.006083 | 0.415 |
| Trichosporonaceae | OM | -0.10051 | 0.781 |
| Trichosporonaceae | TN | 0.068334 | 0.285 |
| Trichosporonaceae | AN | -0.14609 | 0.914 |
| Trichosporonaceae | TP | -0.11269 | 0.791 |
| Trichosporonaceae | AP | 0.095722 | 0.247 |
| Trichosporonaceae | TK | 0.049637 | 0.276 |
| Trichosporonaceae | AK | -0.04201 | 0.59 |
| Trimorphomycetaceae | pH | -0.10045 | 0.745 |
| Trimorphomycetaceae | OM | -0.09049 | 0.662 |
| Trimorphomycetaceae | TN | 0.060675 | 0.287 |
| Trimorphomycetaceae | AN | -0.19047 | 0.991 |
| Trimorphomycetaceae | TP | -0.0596 | 0.589 |
| Trimorphomycetaceae | AP | -0.06245 | 0.494 |
| Trimorphomycetaceae | TK | 0.138431 | 0.105 |
| Trimorphomycetaceae | AK | -0.11173 | 0.812 |

OM, organic matter; TN, total nitrogen; AN, hydrolytic nitrogen; TP, total phosphorus; AP, available phosphorus; TK, total potassium; and AK, available potassium

.
